# Multiplex T Cell Stimulation Assay Utilizing a T Cell Activation Reporter-Based Detection System

**DOI:** 10.3389/fimmu.2020.00633

**Published:** 2020-04-09

**Authors:** Sarah E. Mann, Zhicheng Zhou, Laurie G. Landry, Amanda M. Anderson, Aimon K. Alkanani, Jeremy Fischer, Mark Peakman, Roberto Mallone, Kristen Campbell, Aaron W. Michels, Maki Nakayama

**Affiliations:** ^1^Barbara Davis Center for Childhood Diabetes, University of Colorado School of Medicine, Aurora, CO, United States; ^2^CNRS, INSERM, Institut Cochin, Université de Paris, Paris, France; ^3^Department of Immunobiology, School of Immunology & Microbial Sciences, Faculty of Life Sciences & Medicine, King’s College London, London, United Kingdom; ^4^Assistance Publique - Hôpitaux de Paris, Service de Diabétologie et Immunologie Clinique, Cochin Hospital, Paris, France; ^5^Department of Pediatrics, University of Colorado School of Medicine, Aurora, CO, United States; ^6^Department of Immunology & Microbiology, University of Colorado School of Medicine, Aurora, CO, United States

**Keywords:** antigens, epitopes, T cell receptors, multiplex assay, reporter

## Abstract

Recent advancements in single cell sequencing technologies allow for identification of numerous immune-receptors expressed by T cells such as tumor-specific and autoimmune T cells. Determining antigen specificity of those cells holds immense therapeutic promise. Therefore, the purpose of this study was to develop a method that can efficiently test antigen reactivity of multiple T cell receptors (TCRs) with limited cost, time, and labor. Nuclear factor of activated T cells (NFAT) is a transcription factor involved in producing cytokines and is often utilized as a reporter system for T cell activation. Using a NFAT-based fluorescent reporter system, we generated T-hybridoma cell lines that express intensely fluorescent proteins in response to antigen stimulation and constitutively express additional fluorescent proteins, which serve as identifiers of each T-hybridoma expressing a unique TCR. This allows for the combination of multiple T-hybridoma lines within a single reaction. Sensitivity to stimulation is not decreased by adding fluorescent proteins or multiplexing T cells. In multiplexed reactions, response by one cell line does not induce response in others, thus preserving specificity. This multiplex assay system will be a useful tool for antigen discovery research in a variety of contexts, including using combinatorial peptide libraries to determine T cell epitopes.

## Introduction

Determining the preferred antigenic epitopes of T cells holds immense therapeutic promise with respect to infectious diseases, autoimmune diseases, and cancer. While T cells are important in the control and elimination of infections as well as the prevention of cancer, self-reactive T cells also play a role in the pathogenesis of autoimmune diseases (1–3). The discovery of T cell antigens can inform the development of personalized therapies for autoimmune diseases and cancer, especially in patients who do not respond to conventional treatments (4). For example, it is known that antigen-specific regulatory T cells are more effective in suppressing autoimmune diseases than polyclonal regulatory T cells (5). Antigen-bearing nanoparticles have also been shown to induce tolerance in autoimmune diseases and allergies (6–10). Furthermore, the determination of antigens along with MHC restriction has led to studies investigating the use of drugs and antibodies to block the expression or antigen presentation of disease-associated peptide-MHC complexes in autoimmune diseases (11, 12). Another context in which antigen discovery is particularly important is the emerging field of chimeric antigen receptor (CAR) T cell therapy, which has produced very favorable results in clinical trials (13). However, one of the challenges limiting the advancement of these therapies is the availability of techniques that facilitate rapid and cost-effective discovery of disease-specific antigens.

Many classical methods of determining disease-associated T cell antigens examine antigen reactivity within polyclonal T cell populations, which are often comprised of peripheral blood mononuclear cells (PBMCs) from both patients and disease-free subjects. Techniques include ELISA, ELISPOT, and proliferation assays that evaluate T cell responses to peptides added in culture. The ELISPOT assay, because of its simplicity and sensitivity, is particularly useful for monitoring clinical trials (14–16). Cell proliferation assays that estimate cell division by dilution of incorporated fluorescent dyes such as carboxyfluorescein succinimidyl ester (CFSE) to monitor cell response to antigen stimulation (17) allow for the isolation of antigen-specific cells stimulation using flow cytometry (18). These assays are therefore useful once *a priori* knowledge about candidate antigens is known, but are not ideal for screening a large number of peptides. Their application in the context of tissue-specific autoimmune diseases is also limited because self-reactive T cells are extremely rare in circulating blood (thus posing a sensitivity challenge), and their affinity to target epitopes is often low (thus resulting in specificity issues) (2, 19).

Monoclonal T cell populations, such as traditional T cell clones or hybridoma cells, are often used to study antigen specificity. Characterization of traditional T cell clones is especially preferred when characterizing phenotypes and functions of T cells. However, it is generally difficult to produce large numbers of cells repeatedly and stably without specific skills (19). T cell clones also decrease in responsiveness to antigen and become functionally unstable after long-term culture or multiple freeze-thaw cycles (20, 21), which limits the possibility for testing large panels of antigens and reduces options for different downstream applications. Hybridoma cells, on the other hand, are immortalized cells generated by fusing T cells with a tumor cell line (22). Advantages of T-hybridoma cells include their monoclonality, reproducibility, stability, and capacity to receive genetic manipulation (23). In the present study, we used mouse T cell-derived hybridomas called 5KC cells, which do not express endogenous T cell receptors (TCRs), to express human chimeric TCRs of interest (21, 22) along with an activation reporter and cell-hashing indicators for multiplexing. 5KC cells are derived from a mouse CD4 T cell (22), and therefore TCRs need to be assembled from human variable regions and mouse constant regions to allow for functional TCR signaling. Nevertheless, we use 5KC T-hybridoma cells to express TCRs of interest rather than human immortalized T cell lines such as Jurkat cells because we have observed that 5KC cells provide sensitive and robust response to antigen stimulation.

The NFAT family of transcription factors consists of five members and is expressed by a wide range of cell types. Upon T cell activation, NFAT is activated and translocated to the nucleus, where it regulates the production of cytokines, including IL-2 (24), and has been used as a reporter of T cell activation in a variety of studies (24–28). In the present study, 5KC T-hybridomas were transduced with viral vectors containing the NFAT binding sequences upstream of the gene for a fluorescent reporter protein. Thus, upon T cell activation, NFAT is produced and the accompanying fluorochrome is expressed. Advantages of the NFAT-reporter system include multiplexing, which allows for the screening of multiple T-hybridoma cells in a single reaction, and the ability to sort antigen-specific cells out of a polyclonal population without traditional cloning procedures. We have applied this NFAT-reporter system to 5KC T-hybridomas to establish a multiplex assay technique in which up to eight monoclonal TCRs can simultaneously be evaluated for response to antigen stimulation. Incorporation of additional fluorescent proteins as identifiers allows multiple T cell lines expressing different TCRs to be added together in a single well of a stimulation assay and distinguished via flow cytometry. This multiplex assay system allows for powerful screening of multiple clonotypic T cells for response to a large number of peptides at one time without sacrificing sensitivity and specificity, thus facilitating antigen discovery studies with limited time, cost, and labor.

## Materials and Methods

### Vectors

#### NFAT-Reporter Retroviral Vectors

**FIGURE 1:**
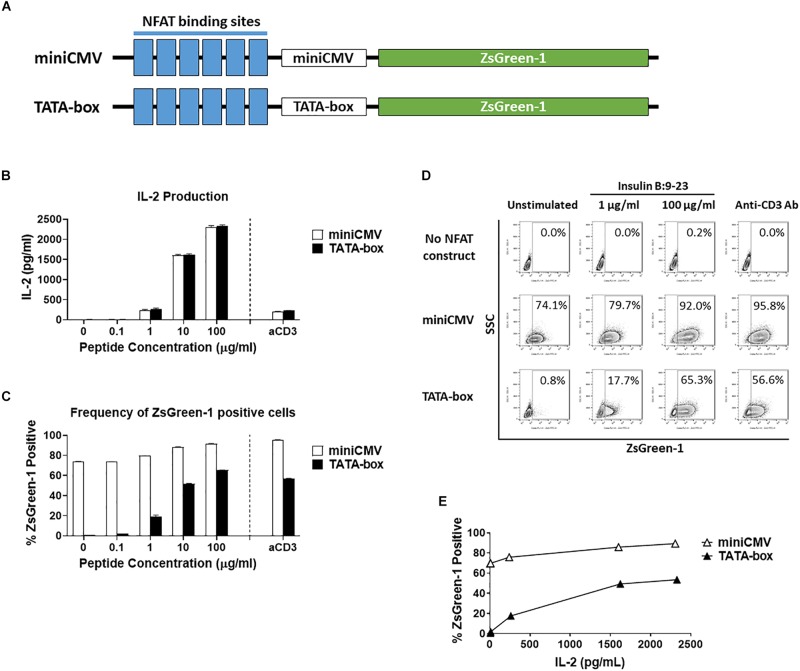
Comparison of assistant promoters. **(A)** Schematic showing the relative positions of NFAT binding sites, assisting promoters, and ZsGreen-1 gene used in the NFAT-reporter constructs. **(B–E)** GSE.6H9 TCR 5KC T-hybridoma cells transduced with an NFAT-reporter construct containing the either miniCMV promoter or TATA-box were cultured with anti-CD3 antibody or different concentrations of the cognate peptide, insulin B:9-23, in the presence of K562 cells expressing HLA-DQ8. IL-2 production in culture supernatant was measured by ELISA **(B)**, and frequency of ZsGreen-1-positive 5KC T-hybridoma cells was measured by flow cytometry using the gating strategy shown in Supplementary Figure S1 **(C)**. Mean values ± standard error of the mean (SEM) from experiments performed in duplicate are shown. **(D)** Representative flow cytometry contour plots showing percentage of ZsGreen-1-positive cells containing either the miniCMV promoter or the TATA-box. Cells without an NFAT reporter construct are included to show the baseline fluorescent intensity that was used to establish gate placement for ZsGreen-1 positive cells. **(E)** Correlation between ZsGreen-1 positivity and IL-2 production for both cell lines. All results are representative of three independent experiments.

**FIGURE 2:**
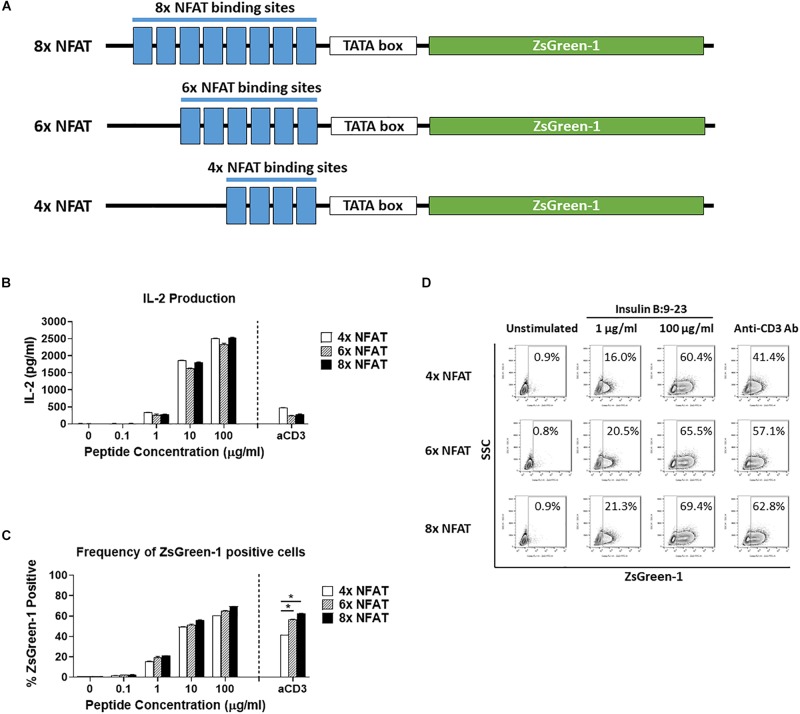
Comparison of number of NFAT binding site repeats. **(A)** Schematic showing the three NFAT-reporter constructs used in the experiments, containing either four, six, or eight copies of the NFAT binding site. **(B–D)** GSE.6H9 TCR 5KC T-hybridoma cells transduced with an NFAT-reporter construct containing four, six, or eight copies of the NFAT binding site were cultured with anti-CD3 antibody or different concentrations of the cognate peptide, insulin B:9-23, in the presence of K562 cells expressing HLA-DQ8. IL-2 production in culture supernatant was measured by ELISA **(B)**, and frequency of ZsGreen-1-positive 5KC T-hybridoma cells was measured by flow cytometry using the gating strategy shown in Supplementary Figure S1 **(C)**. Mean values ± SEM from experiments performed in duplicate are shown. *p-values calculated by unpaired *t*-test were < 0.01. **(D)** Representative flow cytometry contour plots showing percentage of ZsGreen-1-positive cells containing either the 4x NFAT, 6x NFAT, or 8x NFAT construct. Unstimulated cells containing each construct are included to show the baseline fluorescent intensity that was used to establish gate placement for ZsGreen-1 positive cells. All results are representative of three independent experiments.

**FIGURE 3:**
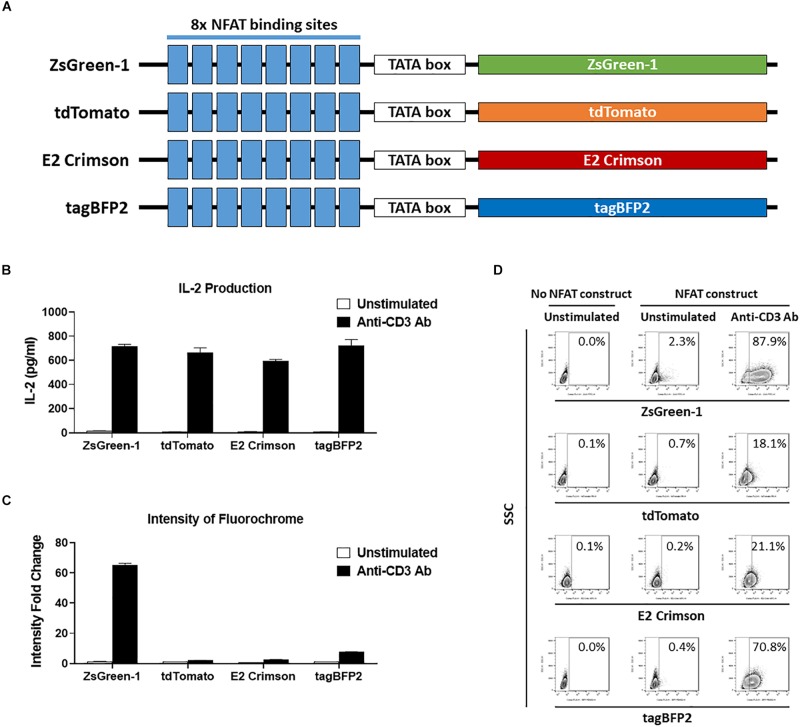
Comparison of reporter fluorochromes. **(A)** Schematic showing the constructs that contained one of four potential fluorescent reporter genes. All constructs contain eight repeats of the NFAT binding site followed by a TATA-box. **(B–D)** 1E6 TCR 5KC T-hybridoma cells transduced with an NFAT-reporter construct containing each fluorescent reporter protein gene were cultured with or without anti-CD3 antibody overnight in the presence of K562 cells expressing HLA-A2. Values in panels **(B)** and **(C)** represent mean ± SEM from experiments performed in duplicate. **(B)** IL-2 production in culture supernatant was measured by ELISA. **(C)** Cells harvested from each culture condition were assessed on flow cytometer with 405, 488, and 633 nm laser beams. Fluorescent intensity of 5KC T-hybridomas, selected by the gating strategy shown in Supplementary Figure S1, was determined. Fold change in mean fluorescent intensity of 5KC T-hybridomas transduced with an NFAT-reporter construct containing each fluorochrome over 5KC T-hybridomas without an NFAT-reporter construct is shown. **(D)** Representative flow cytometry contour plots showing proportions of stimulated and unstimulated fluorescent cells, each expressing a different fluorescent reporter protein. Cells without reporter construct are included to show baseline fluorescent intensity that was used to establish gate placement of fluorescence-positive cells. All results are representative of three independent experiments.

Nuclear factor of activated T cells-reporter genes were inserted in a retroviral vector that contains an inactivated 3′LTR. We generated a backbone retroviral vector in which the CMV 5′LTR and inactivated MoMuLV 3′LTR were replaced with the 5′LTR and 3′LTR regions of the murine stem cell virus (MSCV) vector (Clontech MSCV Retroviral Expression System). Mutated human CD4, with two amino acid substitutions at positions 40 (Q40Y) and 45 (T45W) to increase affinity between CD4 and MHC molecules (21, 29), or human CD8 (hCD8) genes were inserted downstream of the PGK promoter in between the 5′LTR and inactivated 3′LTR. The resulting two backbone retroviral vectors were named pSIREN-muhCD4 and pSIREN-hCD8 (Supplementary Table S1 and Supplementary Document 1). Using a Gibson assembly kit (New England Biolabs), each NFAT reporter construct depicted in Figures 1A, 2A, 3A was inserted into the cloning site of pSIREN-muhCD4 or pSIREN-hCD8 after digestion with BglII and EcoRI as designated in Supplementary Table S1. Vector maps and sequence information are available in Supplementary Document 1.

#### TCR and CD3 Complex Genes Retroviral Vectors

TCR retroviral vectors were generated as described previously (3, 21). Briefly, double-stranded DNA fragments encoding TCR alpha and beta chains were synthesized by TWIST (TWIST Bioscience, San Francisco, CA, United States) based on sequence information determined from a preproinsulin-reactive T cell clone (30, 31) for 1E6, the published sequence information for GSE.6H9 (3), and TCR sequences identified from preproinsulin-unresponsive CD8 T cells in the islets of organ donors (3). DNA fragments encoding the TCR alpha chain variable region, TCR alpha chain constant region followed by the porcine teschovirus-1 2A (P2A) peptide sequence (GSG-P2A: GGC TCT GGA GCC ACG AAC TTC TCT CTG TTA AAG CAA GCA GGA GAC GTG GAA GAA AAC CCC GGA CCT), TCR beta chain variable region, and TCR beta constant region were introduced via Gibson assembly (New England Biolabs) into a murine stem cell virus (MSCV)-based retroviral vector (32, 33). Note that TCR alpha and beta chain variable regions are derived from human T cells while sequences of TCR alpha and beta chain constant regions are murine. The resulting chimeric TCR genes are compatible with murine 5KC T-hybridoma cells to transmit TCR signaling provided by interaction between human TCR variable regions and peptide-MHC complexes.

The retrovirus plasmid containing the murine CD3 genes (CD3γ, CD3δ, CD3ε, and the ζ chain (CD247), linked by 2A peptides) along with the ametrine fluorescent protein gene was a kind gift from Dr. Dario Vignali (34), and the retroviral vector containing the CD3 genes along with the E2-Crimson gene was generated in the Nakayama laboratory by replacing the ametrine gene with the E2-Crimson gene.

#### HLA Retroviral and Lentiviral Vectors

A double-stranded DNA fragment encoding the HLA-A^∗^02:01 gene was synthesized by TWIST (TWIST Bioscience, San Francisco, CA) based on the sequence published in the IPD-IMGT/HLA Database^1^. An MSCV-based retroviral vector carrying the HLA-A^∗^02:01 gene and the human beta2 microglobulin gene (NCBI BC064910) was generated as described previously (3, 21). An MSCV-based retroviral vector carrying the HLA-DQ8 (DQA1^∗^03:01 and DQB1^∗^03:02) described in our previous report (3) was used to express HLA-DQ8. A lentiviral HLA-A2 vector was generated by digesting the vector pMH0001, a gift from Jonathan Weissman (Addgene plasmid # 85969; RRID: Addgene_85969)^2^ (35), with MluI and SbfI and inserting the HLA-A2 gene cassette (A^∗^02:01-P2A-human beta2 microglobulin).

#### Fluorescence Retroviral Vectors

One each of the following identifying fluorescent protein genes was inserted into an MSCV-based retroviral vector (32, 33): ametrine (EU024649)^3^, LSSmOrange (NCBI BAR40058)^4^, mTagBFP2 (BBB44440)^5^, tdTomato (AMO27245)^6^, E2-Crimson (AMO27251)^7^, mCherry (QBQ65831)^8^.

The retrovirus plasmid containing the murine CD3 genes (CD3γ, CD3δ, CD3ε, and the ζ chain (CD247) linked by 2A peptides) along with the ametrine fluorescent protein gene was kindly provided by Dr. Dario Vignali (34). To generate a retroviral vector carrying the murine CD3 genes along with LSSmOrange, the ametrine gene was replaced with the LSSmOrange gene. All vectors are available upon request.

### Cells

5KC T-hybridoma cells (5KC_73.8.20) (22) carrying an NFAT-reporter construct and TCR genes were generated by spin-infection with replication-incompetent retroviruses as described previously (3, 21). For multiplex assays, 5KC T-hybridoma cells were further transduced with two fluorescent protein genes as identifiers along with CD3 complex genes to stabilize expression of TCR-CD3 complexes. Transduced cells were selected according to the genes that were carried by the plasmids using either magnetic-activated cell sorting (Miltenyi Biotec) or flow cytometric sorting on a MoFlo Astrios EQ (Beckman Coulter). 5KC T-hybridoma cell lines used in each experiment are summarized in Supplementary Table S2.

K562 cells (ATCC CCL-243) transduced with cognate HLA genes were used as APCs. Retroviruses and lentiviruses were made by chemical transfection (Lipofectamine 2000, Thermo Fisher Scientific) of 293T cells (ATCC CRL-3216) with the packaging vectors pVSVG and pEQ-Pam3 (32) or 293FT cells with the packaging vectors pMD2.G and psPAX2 (University of Colorado Functional Genomics Shared Resource). K562 cells were transduced with viruses containing HLA genes with or without the LSSmOrange gene by spin-infection as described previously (3, 21). Transduced cells were selected according to the genes carried by the plasmids using flow cytometric sorting on a MoFlo Astrios EQ (Beckman Coulter).

K562 cells and 5KC T-hybridoma cells were cultured in IMDM culture media (Gibco 12440) supplemented with 10% fetal bovine serum, 100 units/ml penicillin / 100 μg/ml streptomycin (Sigma), and 2-Mercaptoethanol (Sigma) at 143 μM.

To generate immortalized autologous B cells lines, spleen cells of TCR donors were infected with Epstein-Barr virus that were harvested from supernatant of B95-8 cell culture. Autologous B cell lines were used as APCs for experiments shown in Figure 5A. Spleen cells of TCR donors were obtained from the JDRF nPOD consortium (36, 37) with approval as exempt human subjects research by the Colorado Multiple Institutional Review Board.

#### Peptides

The two cognate peptides for GSE.6H9 and 1E6 TCRs, (InsB:9-23, SHLVEALYLVCGERG) and (PPI:15-24, ALWGPDPAAA), were synthesized with purity greater than 95% by Genemed Synthesis (San Antonio, Texas). The truncated peptide pools derived from preproinsulin were generated by Mimotopes (Victoria, Australia). The truncated preproinsulin peptide panel is composed of peptide pools containing equimolar amounts of 8- to 11-mers ending at the same amino acid of the protein. Peptides in each pool are derived from a different portion of the protein of interest, thus all potential T cell epitopes can be assessed in one panel (Supplementary Tables S3). The combinatorial peptide library (CPL) (Pepscan, Lelystad, Netherlands) is composed of 200 decamer peptide pools. Each peptide pool contains decamer peptides with a fixed amino acid residue at one position and randomized L-amino acids, with the exception of cysteine, at the other positions, resulting in 3.2 × 10^11^ (=19^9^) peptides in each pool (Supplementary Table S4) (38, 39).

#### T Cell Stimulation Assays

**FIGURE 4:**
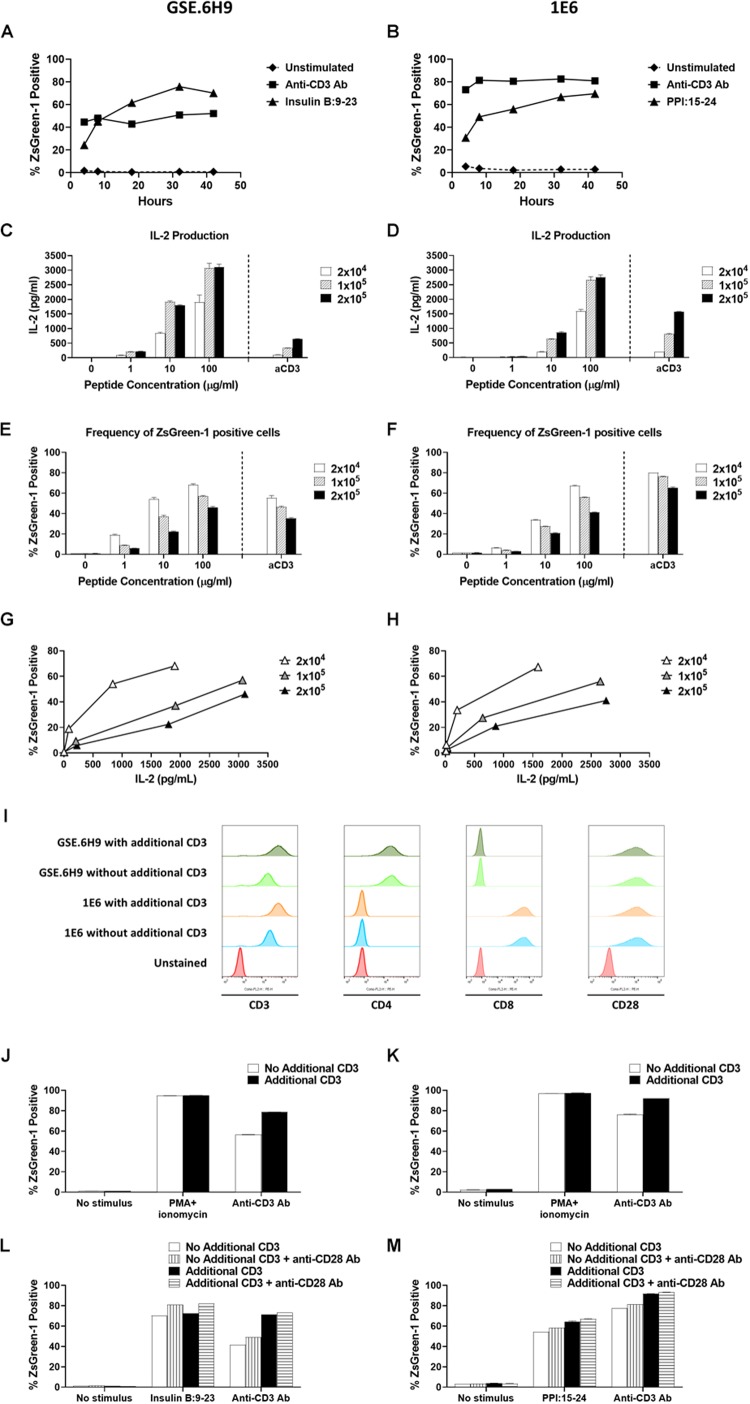
Establishment of assay conditions. **(A)** and **(B)** 5KC T-hybridoma cells expressing an insulin B:9-23-responsive CD4 TCR GSE.6H9 **(A)** or a preproinsulin 15-24-responsive CD8 TCR 1E6 **(B)** were unstimulated or were stimulated with anti-CD3 antibody or the cognate peptide in the presence of K562 APCs expressing HLA-DQ8 or HLA-A2, respectively. Percentage of ZsGreen-1-positive cells, determined using the gating strategy shown in Supplementary Figure S1, was assessed via flow cytometry at 4, 8, 18, 32, and 42 h post-stimulation. Mean values ± SEM from experiments performed in duplicate are shown. **(C–H)** Different numbers (2 × 10^4^, 1 × 10^5^, or 2 × 10^5^ cells/well) of 5KC T-hybridoma cells expressing the GSE.6H9 TCR **(C,E,G)** or 1E6 TCR **(D,F,H)** were unstimulated or were stimulated with anti-CD3 antibody or the cognate peptide in the presence of K562 APCs (5 × 10^4^ cells/well) expressing HLA-DQ8 or HLA-A2, respectively. IL-2 production was measured by ELISA **(C,D)** and frequency of ZsGreen-1 positive 5KC T-hybridoma cells was assessed by flow cytometry using the gating strategy shown in Supplementary Figure S1 **(E,F)**. Values in panels **(C–F)** represent mean ± SEM from experiments performed in duplicate. Panels **G** and **H** show correlation between ZsGreen-1 positivity and IL-2 production for each concentration of T-hybridoma cells expressing GSE.6H9 TCR **(G)** and 1E6 TCR **(H)**. **(I)** 5KC T-hybridoma cells expressing the GSE.6H9 TCR or the 1E6 TCR with or without additional CD3 genes were stained with anti-mouse CD3, human CD4, human CD8, or mouse CD28 antibodies and assessed by flow cytometry. **(J–M)** 5KC T-hybridomas expressing the GSE.6H9 TCR **(J,L)** or the 1E6 TCR **(K,M)** were further transduced with additional CD3 complex genes and stimulated with a stimulation cocktail containing PMA and ionomycin or anti-CD3 antibody for 6–8 h **(J,K)**, or cognate peptides or anti-CD3 antibody in the presence or absence of anti-CD28 antibody overnight **(L,M)**. Frequency of ZsGreen-1 positive T-hybridoma cells was assessed by flow cytometry. All results are representative of three independent experiments.

**FIGURE 5:**
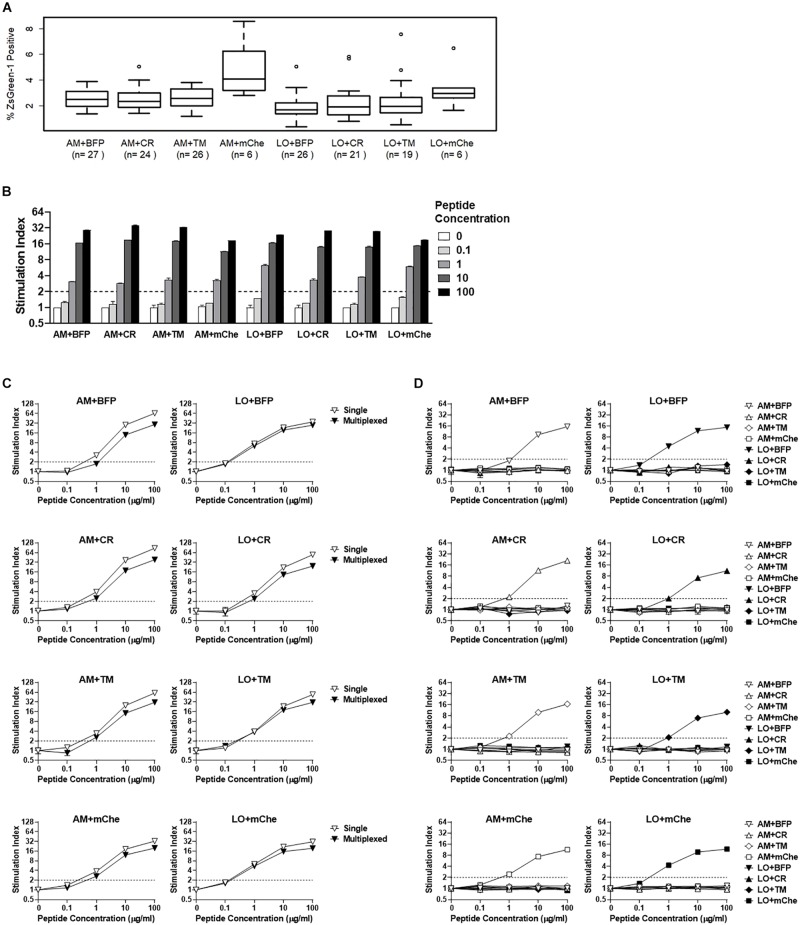
Addition of fluorochrome identifiers and multiplexing. **(A)** ZsGreen-1 positivity of unstimulated cells expressing TCRs with different fluorochrome combinations. Each 5KC T-hybridoma cell line expresses a single unique TCRs along with one of eight different fluorochrome combinations. 5KC T-hybridoma cells were cultured overnight in the presence of autologous B cells transformed with Epstein-Barr virus. Frequency of ZsGreen-1-positive 5KC T-hybridoma cells expressing eight different fluorochrome combinations was determined by flow cytometry using the gating strategy shown in Supplementary Figure S2 and were compared using one-way ANOVA and Tukey’s Honestly Significant Difference (Tukey’s HSD). Numbers of TCRs that are included for each fluorochrome combination are shown in parenthesis. T-hybridoma cells expressing ametrine and mCherry (AM+mChe) have significantly higher ZsGreen-1 positivity compared to T-hybridomas expressing all other fluorochrome combinations except LO+CR (Turkey’s *p* = 0.001 in AM+mChe vs. AM+BFP, AM+CR, AM+TM, and Turkey’s *p* < 0.001 in AM+mChe vs. LO+BFP, LO+CR, LO+TM). **(B)** 1E6 TCR T-hybridoma cells expressing eight different fluorochrome combinations were unstimulated or were stimulated with the cognate preproinsulin 15-24 peptide at 0.1, 1, 10, and 100 μg/ml in the presence of K562 APCs expressing HLA-A2. Frequency of ZsGreen-1-positive 5KC T-hybridoma cells were determined by flow cytometry using the gating strategy shown in Supplementary Figure S2. Stimulation indexes were calculated by dividing ZsGreen-1 positivity of stimulated cells by mean values of ZsGreen-1 positivity of unstimulated cells. **(C)** 1E6 TCR T-hybridoma cells expressing each fluorochrome combination were co-cultured with the cognate peptide and HLA-A2 K562 cells in the presence (multiplexed, black inverse triangles) or absence (single, white inverse triangles) of T-hybridomas expressing irrelevant TCRs expressing the other seven fluorochrome combinations. Stimulation indexes of 1E6 TCR T-hybridomas in response to the peptide stimulation were calculated as described above based on frequency of ZsGreen-1-positive 1E6 TCR T-hybridoma cells determined by flow cytometry using a gating strategy shown in Supplementary Figure S2. **(D)** 1E6 TCR T-hybridoma cells expressing each fluorochrome combination were co-cultured with the cognate peptide and HLA-A2 K562 cells in the presence of T-hybridomas expressing irrelevant TCRs and the other seven fluorochrome combinations. Frequencies of ZsGreen-1-positive cells for T-hybridomas expressing each fluorochrome combination in each culture condition were determined by flow cytometry, and stimulation indexes were calculated as described above. **(B–D)** Values represent mean ± SEM from three independent experiments performed in duplicate.

**FIGURE 6:**
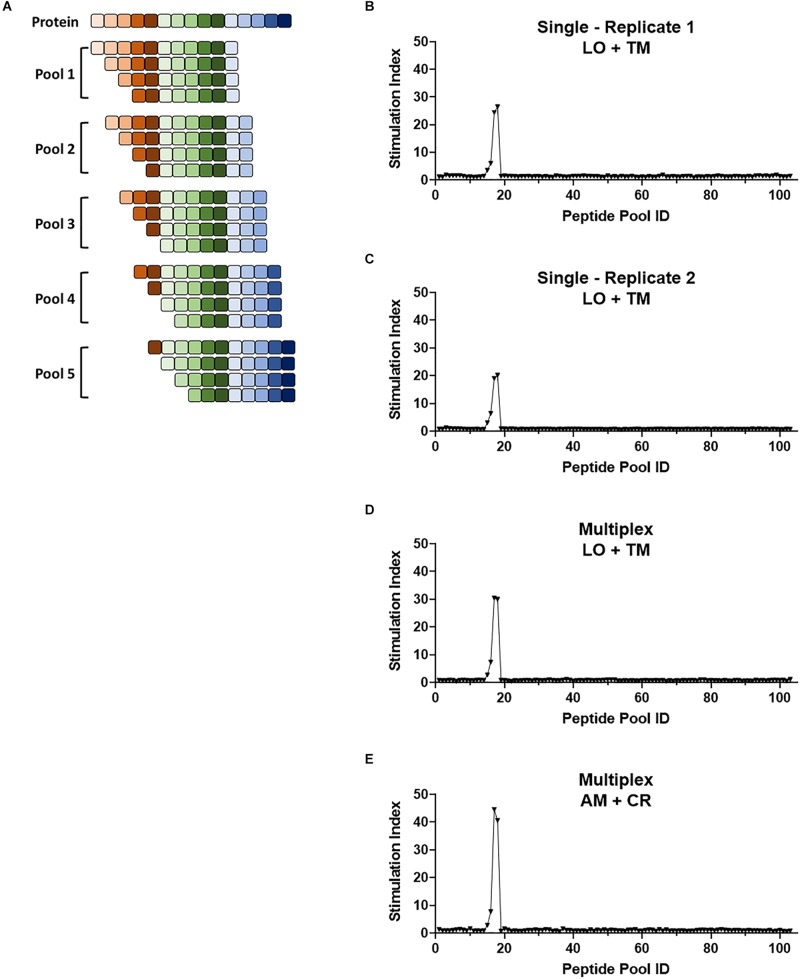
Multiplex screening for a large panel of peptide libraries. **(A)** Schematic showing the breakdown of a protein of interest into pools of 8- to 11-mers such that each potential epitope of the peptide is represented in the peptide panel. Pools contain equimolar amounts of each truncated peptide. **(B–E)** 1E6 TCR T-hybridoma cells expressing LSSmOrange and tdTomato **(B–D)** or ametrine and E2-Crimson **(E)** were co-cultured with 103 truncated peptide pools generated from preproinsulin and HLA-A2 K562 cells in the absence [**(B,C)**, single test] or presence [**(D,E)**, multiplex test] of T-hybridomas expressing irrelevant TCRs with the other seven fluorochrome combinations. Stimulation indexes of 1E6 TCR T-hybridomas in response to the peptide stimulation were calculated as described in Figure 5 based on frequency of ZsGreen-1-positive 1E6 TCR T-hybridoma cells determined by flow cytometry using a gating strategy shown in Supplementary Figure S2. Results from two independent experiments testing a single T-hybridoma cell line individually are shown in **(B,C)**. In all cell culture conditions testing 1E6 TCR T-hybridoma cell lines expressing different fluorescence identifiers, T-hybridomas responded to peptide pools 15–18 with a peak at 17 and 18. These pools contain the cognate peptide PPI:15-23 and PPI:15-24.

**FIGURE 7:**
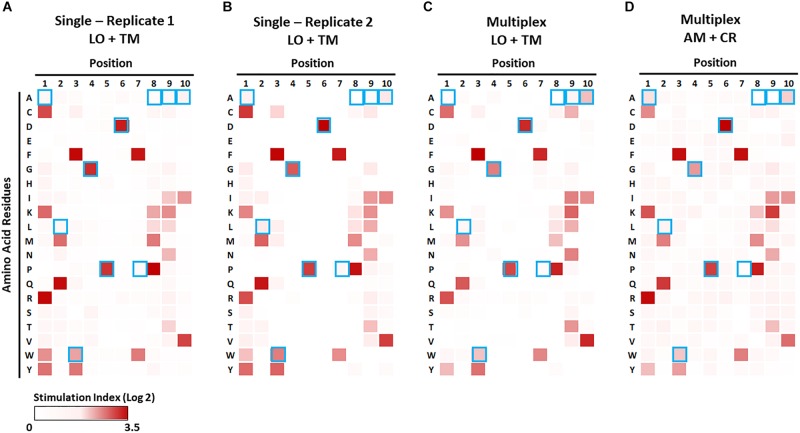
Identification of preferable amino acid residues in epitopes using a combinatorial peptide library (CPL). The CPL consists of 200 decamer peptide pools, each of which contains peptides with a specific amino acid residue at one position and randomized amino acids at the other nine positions (Supplementary Table S4). 1E6 TCR T-hybridoma cells expressing LSSmOrange and tdTomato **(A–C)** or ametrine and E2-Crimson **(D)** were co-cultured with each CPL peptide pool along with K562 APCs lentivirally expressing HLA-A2 in the absence [**(A,B)**, single test] or presence [**(C,D)**, multiplex test] of T-hybridomas expressing irrelevant TCRs and the other seven fluorochrome combinations. Stimulation indexes of 1E6 TCR T-hybridomas in response to the peptide stimulation were calculated as described in Figure 5 based on frequency of ZsGreen-1-positive 1E6 TCR T-hybridoma cells as determined by flow cytometry using the gating strategy shown in Supplementary Figure S2. Log2 fold changes of stimulation indexes in each culture condition were depicted as heat maps. Results from two independent experiments testing a single T-hybridoma cell line individually are shown in **(A,B)**. Blue boxes represent the amino acids of the nominal epitope ALWGPDPAAA at each position.

In round-bottom 96-well plates, 5KC T-hybridoma cells and APCs were cocultured with or without peptides or anti-mouse CD3ε antibody (BD Pharmigen, clone 145-2C11). K562 cells at 5 × 10^4^ cells/well were used as APCs in all experiments except for those depicted in Figures 4J,K, 5A. In Figures 4J,K, APCs were not added, and in Figure 5A, autologous B cells were added as APCs at 1 × 10^5^ cells/well. For experiments shown in Figures 1–3, 4A,B,J–M, 5KC T-hybridoma cells were added at 1 × 10^5^ cells/well. In the experiments presented in Figures 4C–H, 5KC T-hybridoma cells were added at 2 × 10^4^ cells/well, 1 × 10^5^ cells/well, and 2 × 10^5^ cells/well as designated in each panel. In Figures 5–7, 5KC T-hybridoma cells were added at 2 × 10^4^ cells/well when single 5KC lines were tested. When multiplexed, each 5KC T-hybridoma expressing different fluorochrome combinations was added at 2 × 10^4^ cells/well, resulting in a total of 1.6 × 10^5^ cells of all eight T-hybridoma lines per well. Insulin B:9-23 and PPI:15-24 were added at final concentrations of 0.1, 1, 10 or 100 μg/ml for experiments shown in Figures 1–5 except Figures 4A,B,L,M, in which cells were cocultured with peptides at 100 μg/ml only. In the experiments presented in Figures 4J,K, a cocktail of phorbol-12-myristate 13-acetate (PMA) at 81 nM and ionomycin at 1.34 μM (eBioscience) were added to 5KC T-hybridoma cells as designated in the figure without APCs. Truncated peptide pools, used in the experiments shown in Figure 6, were added at final concentrations between 120 μg/ml and 320 μg/ml. CPL peptide pools were added a final concentration of 100 μg/ml. The CPL assays also included 10 wells of cultures without peptides to be used as negative controls. Anti-CD3 antibody and anti-CD28 antibody (eBioscience, clone 37.51) were added at a final concentration of 5 μg/ml. Cells were cultured overnight prior to being analyzed for IL-2 production and reporter fluorescent protein expression in all assays except those presented in Figures 4A,B, in which cells were cultured as designated in the section “Results” and figures, and Figures 4J,K, in which cells were cultured for 6 to 8 h.

#### Measurement of IL-2 by ELISA

IL-2 production into the culture supernatant was measured by ELISA using the capture antibody (BD Pharmigen, 554424), secondary biotinylated antibody (BD Pharmigen, 554426), Europium-labeled Streptavidin (Perkin Elmer, 1244-360), and DELFIA enhancement solution (Perkin Elmer). Plates were read on a Wallac 1420 Victor2 Multilabel Counter (Perkin Elmer) using the time-resolved fluorescence-based Europium setting.

#### Flow Cytometry

Fluorescence of 5KC T-hybridoma cells was analyzed by flow cytometry on a Beckman Coulter Cytoflex containing 405, 488, and 633 nm lasers. Culture plates were pelleted by centrifugation at 671 *g* for 3 min. After removing supernatant, cells were resuspended in 150 μl RPMI 1640 without phenol red (Gibco 11835) supplemented with 1% fetal bovine serum (Atlanta Biologicals).

When only one line of 5KC T-hybridoma cells was included in the culture reaction (i.e., analysis of a single TCR), 30,000–50,000 cells were acquired during flow cytometry. For multiplex assays (i.e., analysis of multiple TCRs in a single well), 75,000 cells were acquired. Typically, 3,000–5,000 5KC T-hybridoma cells expressing each TCR were assessed for production of ZsGreen-1 using the gating strategy depicted in Supplementary Figures S1, S2 for experiments shown in Figures 1–7, respectively. Flow cytometric analysis was performed using FlowJo Version 10. To assess T cell activation after peptide stimulation for experiments shown in Figures 5, 6, a stimulation index (SI) was calculated according to the formula below.

$$SI\;\;\;\;\;\frac{\;\;\;\;\;\;\;\;\;\;\;\;\begin{array}{c} ZsGreen-1\;positivity\;\left(\%\right)\\ in\;stimulated\;5KC\;\text{T-hybridoma}\;cells \end{array}\;\;\;\;\;\;\;\;\;\;\;\;}{\begin{array}{c} ZsGreen-1\;positivity\;\left(\%\right)\\ in\;unstimulated\;5KC\;\text{T-hybridoma}\;cells \end{array}}$$

To assess T cell activation after CPL stimulation (Figure 7), a stimulation index was calculated using the formula below.

$$SI\;\;Log2(\frac{\begin{array}{c} ZsGreen-1\;positivity\;\left(\%\;\right)\;in\\ stimulated\;5KC\;\text{T-hybridoma}\;cells \end{array}\;}{\begin{array}{c} Mean\;ZsGreen-1\;positivity\;of\;5KC\;\text{T-hybridoma}\\ \;cells\;in\;10\;unstimulated\;wells \end{array}})$$

To assess CD3, CD28, CD4, and CD8 expression on 5KC cell surface, cells were stained with anti-mouse CD3ε antibody conjugated with phycoerythrin (PE) (BD Pharmigen, clone 145-2C11), anti-mouse CD28 antibody conjugated with PE (BioLegend, clone 37.51), anti-human CD4 antibody conjugated with PE (BioLegend, clone RPA-T4), and anti-human CD8 antibody conjugated with PE (BioLegend, clone SK1), followed by analysis of PE signals on a Beckman Coulter Cytoflex.

#### Statistics

To assess the correlation between IL-2 production and frequency of ZsGreen-1-positive cells, R-squared and *p*-values were calculated using a simple linear regression model. To assess the difference in response to anti-CD3 stimulation, *p*-values were calculated by unpaired *t*-test. Analysis was performed using GraphPad Prism 8.3.0.

To visually assess the difference in frequency of ZsGreen-1-positive 5KC T-hybridoma cells expressing eight individual fluorochrome combinations in the unstimulated condition, box and whisker plots by group were produced (Figure 5A). To compare the ZsGreen-1 frequency by fluorochrome combination, one-way Anova (ANOVA) was used. The overall *F*-test was reported to test for any differences in ZsGreen-1 frequency by fluorochrome combination, and Tukey’s Honestly Significant Difference (Tukey’s HSD) was used to perform pairwise comparisons while maintaining an alpha of 0.05. Analysis was performed using R version 3.6.0.

## Results

### Development of NFAT-Reporter Constructs

To generate an NFAT-reporter T cell line that sensitively and specifically expresses fluorescent proteins in response to antigen stimulation, we assessed three factors in NFAT-reporter constructs: assisting promoter sequences, the number of NFAT binding site repeats, and different fluorochromes as reporter proteins. We first evaluated assistant promoter sequences (40) that can maximize reporter gene transcription in 5KC T-hybridoma cells. The NFAT construct was generated with six NFAT binding site repeats followed by either a miniCMV promoter or a synthetic TATA-box sequence driving the production of the fluorescent reporter protein, ZsGreen-1 (Figure 1A). In order to determine which promoter provides a more sensitive and specific representation of cell activation, we compared ZsGreen-1 expression and IL-2 production in two 5KC T-hybridoma cell lines containing the NFAT construct with either the miniCMV promoter or TATA-box. These cells expressing a TCR that recognizes an insulin peptide presented by human leukocyte antigen (HLA) DQ8 molecule, were cultured overnight with an anti-CD3 monoclonal antibody or cognate peptide in the presence of antigen-presenting cells (APCs) expressing HLA-DQ8. IL-2 production was similar between both cell lines in all treatment conditions, confirming that cell conditions in response to stimulation were equivalent (Figure 1B). However, cells transduced with the miniCMV promoter produced high levels of ZsGreen-1 even in the absence of stimulation (Figures 1C,D). On the other hand, the TATA-box did not induce ZsGreen-1 expression without stimulation, and frequency of ZsGreen-1-producing cells in response to peptide stimulation was initiated and increased in a dose-dependent manner (Figures 1C,D), which resulted in a correlation between IL-2 production and frequency of ZsGreen-1-positive cells (Figure 1E, R squared = 0.94, *p* < 0.01). Based on these results, we selected the NFAT construct containing the TATA-box sequence for further use.

We next determined how the number of NFAT binding site repeats influenced expression of the reporter protein. We transduced 5KC T-hybridomas with NFAT-reporter constructs that contained four, six, or eight NFAT binding site repeats (Figure 2A) and assessed ZsGreen-1 expression and IL-2 production after culturing in the presence of the cognate insulin peptide or anti-CD3 antibody. The construct containing six NFAT binding site repeats is identical to the one with a synthetic TATA-box sequence described in Figure 1. The amount of IL-2 secreted into culture supernatant in all treatment conditions was similar between the three cell lines (Figure 2B). For all three constructs, only a limited portion of cells expressed ZsGreen-1 in the absence of stimulation, and frequency of cells expressing ZsGreen-1 in response to stimulation by the cognate peptide was similar between cell lines (Figures 2C,D). However, 8x NFAT and 6x NFAT constructs induced ZsGreen-1 expression more efficiently than 4x NFAT constructs upon anti-CD3 antibody stimulation (Figure 2C, *p* < 0.01). Due to the more robust response to stimulation by anti-CD3 antibody, we proceeded with the 8x NFAT constructs for subsequent experiments.

Finally, we evaluated four fluorochromes that can provide intense signals as reporter proteins. We transduced 5KC T-hybridomas with NFAT constructs containing ZsGreen-1 (AVT50845), tdTomato (AMO27245), E2-Crimson (AMO27251), or blue fluorescent protein (tagBFP-2) (BBB44440) downstream of the NFAT binding sites (Figure 3A) and assessed IL-2 production as well as fluorescence intensity in response to stimulation by anti-CD3 antibody on a flow cytometer with 405 nm, 488 nm, and 633 nm laser beams. Cells without an NFAT reporter construct were included in the assay to determine the baseline fluorescence intensity. Despite similar amounts of IL-2 production between the four cell lines (Figure 3B), intensity of ZsGreen-1 fluorescence was enhanced most drastically in response to anti-CD3 stimulation (Figure 3C). Frequency of ZsGeen-1-producing cells treated with anti-CD3 antibody was also of the highest among the four cell lines (Figure 3D). While frequency of positive cells determined by the baseline intensity were similar between cells transduced with the ZsGreen-1 and tagBFP-2 constructs, the increase in ZsGreen-1 fluorescence intensity was more dramatic than that of tagBFP-2, resulting in a clearer discrimination between activated and non-activated cells by ZsGreen-1 compared to tagBFP-2. Based on the intense fluorescence and higher percentage of fluorescent cells when stimulated, we elected to continue using ZsGreen-1 as our reporter gene.

### Establishment of Assay Conditions

To optimize the conditions under which our 5KC T-hybridoma cells best respond to antigen stimulation, we assessed the effect of culturing time and cell concentration on ZsGreen-1 production. We cultured 5KC T-hybridoma cells with or without targeted peptides in the presence of APCs and evaluated ZsGreen-1 expression at five different time points until 42 h. We chose two TCRs, GSE.6H9, and 1E6, derived from autoreactive CD4 and CD8 T cells responding to insulin B:9-23 presented by HLA-DQ8 and PPI:15-24 presented by HLA-A2, respectively. For both TCRs, we observed minimal activation from unstimulated cells throughout the culture time (Figures 4A,B). When stimulated by the cognate peptides, about a quarter of cells were expressing ZsGreen-1 at 4 h after stimulation. Frequency of ZsGreen-1-producing cells increased until 18 h (Figures 4A,B). Between 18 and 32 h, ZsGreen-1 positivity reached its peak and did not increase considerably. At 42 h, percentage of ZsGreen-1-positive cells had either slightly decreased or remained relatively constant, accompanied by a marked increase in the number of dead cells. When stimulated by anti-CD3 antibody (Figures 4A,B), ZsGreen-1 positivity reached maximum level earlier than peptide-stimulated cells, between 4 and 8 h post-stimulation. Given that levels of response for both peptide-treated and anti-CD3 antibody-treated cells plateaued after 18 h and that cell death increased after 32 h, we concluded that it was most appropriate to measure ZsGreen-1 expression between 18 and 32 h after stimulation.

In order to determine the optimal concentration of T cells in the assay, we cultured 5KC T-hybridoma cells expressing either GSE.6H9 or 1E6 at different concentrations (2 × 10^5^, 1 × 10^5^, and 2 × 10^4^ cells per well) with or without the cognate peptide. All concentrations of 5KC T-hybridoma cells were co-cultured with the cognate peptide in the presence of K562 cells expressing either HLA-DQ8 or HLA-A2, which served as APCs, at 5 × 10^4^ cells per well. Thus, the T cell/APC ratio was tested at 4, 2, and 0.4. Activation of cells was represented by both ZsGreen-1 expression and IL-2 production into culture supernatant. For both cell lines, 2 × 10^4^ cells per well produced the lowest amount of IL-2, but the highest frequency of ZsGreen-1-positive T cells (Figures 4C–F). As T cell numbers increased, IL-2 production also increased while ZsGreen-1 positivity decreased. Cultures containing 2 × 10^4^ cells per well also provided the highest frequency of ZsGreen-1 positive T-hybridoma cells in proportion to IL-2 production (Figures 4G,H). This is presumably because T cells have more opportunities to interact with peptide-HLA complexes as T cell concentration is reduced, and therefore can become more activated. Based on these results, we elected to use the concentration of 2 × 10^4^ 5KC cells per well.

There was a difference in the magnitude of CD3 responses between 5KC T-hybridoma cells expressing the GSE.6H9 and 1E6 TCRs (Figures 4E,F). We previously reported that T-hybridoma cells transduced with additional mouse CD3 complex genes expressed a higher amount of TCR/CD3 complexes, resulting in stronger response to anti-CD3 stimulation (21). Therefore, we introduced additional CD3 genes into T-hybridoma cells and tested reactivity to anti-CD3 stimulation. Of note, the GSE.6H9 and 1E6 cell lines expressed human CD4 and CD8, respectively, at similar levels between cells with and without additional CD3, and all four T-hybridoma lines expressed the equivalent levels of CD28 (Figure 4I). Both T-hybridoma lines expressing the GSE.6H9 and 1E6 TCRs exhibited the maximal activation in response to chemical stimulation using PMA and ionomycin whether or not they expressed additional CD3 molecules (Figures 4J,K), and thus both TCR cell lines retained adequate reporter function when receiving proper stimulus. On the other hand, responses to anti-CD3 stimulation were improved when cells were transduced with the additional CD3 genes (Figures 4J,K), which is compatible with the augmented CD3 expression in these cell lines (Figure 4I). These results suggest that addition of CD3 genes facilitates signaling through TCR/CD3 complexes, resulting in an efficient ZsGreen-1 production. Therefore, we used 5KC T-hybridoma cells transduced with CD3 complex genes in subsequent assays. Responses to antigen and anti-CD3 stimulations were further elevated in the presence of anti-CD28 antibody without elevation of ZsGreen-1 production from unstimulated cells (Figures 4L,M). While anti-CD28 antibody was not added in the following assays, the results suggest additional signaling through co-stimulatory molecules for the improvement of assay sensitivity.

### Addition of Fluorescent Identifiers for Multiplexing

In order to distinguish between multiple T cell lines that are cultured together in a single reaction, we transduced 5KC T-hybridoma cells with two additional fluorochrome genes as identifiers, resulting in a total of eight fluorochrome combinations to identify TCRs (Table 1). Each cell line received either Ametrine or LSSmOrange as well as one of the following: tagBFP-2, E2-Crimson, tdTomato, or mCherry. We first evaluated how the addition of these fluorochrome combinations affected ZsGreen-1 expression in unstimulated cells by comparing ZsGreen-1 positivity from 5KC T-hybridoma cells expressing various TCRs without antigen stimulation. TCR clonotypes analyzed in this experiment were identified in CD8 T cells from the pancreas of organ donors, and each TCR has been expressed in 5KC T-hybridoma cells expressing different fluorochrome combinations. We cultured 5KC T-hybridoma cells with autologous B cell lines established from spleen cells of TCR donors and evaluated ZsGreen-1 expression. Overall, there was a significant difference in frequency of ZsGreen-1 positive cells among cell lines expressing different fluorochrome combinations in the resting state (Figure 5A, *p* < 0.01). This difference was due to the cell line expressing ametrine and mCherry, which had significantly higher ZsGreen-1 positivity than those expressing all other fluorochrome combinations except LSSmOrange and mCherry (Tukey’s adjusted *p* < 0.01). This relatively high frequency of ZsGreen-1-positive cells expressing ametrine and mCherry (average ± standard deviation 4.8% ± 2.4%, median [interquartile range] 4.1% [3.2%–5.9%]) is, however, within the acceptable range to evaluate responses to antigen stimulation. To fairly quantify the intensity of response to stimulation, T cell responses are normalized by dividing ZsGreen-1 positivity of stimulated cells by ZsGreen-1 positivity of unstimulated cells, thereby creating a stimulation index.

**TABLE 1:** Fluorochrome combinations to distinguish between T cell lines that are multiplexed.

	**tagBFP-2 (BFP)**	**E2-Crimson (CR)**	**tdTomato (TM)**	**mCherry (mChe)**
Ametrine (AM)	AM+BFP	AM+CR	AM+TM	AM+mChe
LSSmOrange (LO)	LO+BFP	LO+CR	LO+TM	LO+mChe

We next assessed whether the additional fluorochromes influenced response to antigen stimulation. We expressed the PPI:15-24-reactive 1E6 TCR in 5KC T-hybridoma cells containing each fluorochrome combination and tested them for ZsGreen-1 production in response to the cognate peptide. Note that the minimal peptide concentration necessary to activate 5KC T-hybridoma cells expressing this TCR is approximately 1–10 μg/ml depending on assay conditions and readouts of activation (Figures 4D,F). In all cell lines expressing different fluorochromes, a significant increase of ZsGreen-1 positive cells (i.e., stimulation index >2) was detected once the peptide concentration reached 1 μg/ml, and ZsGreen-1 positivity increased in a dose-dependent manner (Figure 5B). Thus, the sensitivity to antigen stimulation is intact after the addition of fluorescent identifiers.

### Simultaneous Stimulation of T-Hybridoma Cells

We next addressed whether or not the multiplexing of T cells impacts ZsGreen-1 positivity. We analyzed 1E6 TCR-positive 5KC T-hybridoma cells expressing each fluorochrome combination for the response to the cognate peptide in the presence or absence of seven 5KC T-hybridoma cells expressing irrelevant TCRs along with other fluorochrome combinations. When cultured with other 5KC T-hybridoma cells expressing irrelevant TCRs, frequency of ZsGreen-1 positive 1E6 TCR T-hybridomas in response to peptide stimulation was slightly diminished for all color combinations (Figure 5C). However, ZsGreen-1 production was evoked at peptide concentrations as low as 1 μg/ml whether or not T cells were multiplexed (Figure 5C), implying that simultaneous stimulation of multiple cell lines in a single reaction does not influence the sensitivity of T-hybridoma activation in response to antigen stimulation.

5KC T-hybridoma cells expressing irrelevant TCRs might be activated when cultured with cells expressing TCRs reacting to assay peptides by receiving antigen-unspecific stimulation such as cytokine signaling. To address this possibility, we assessed ZsGreen-1 positivity of each cell line within each multiplex reaction to determine if the presence of activated cells results in the activation of other cell lines. In each mixture that contained the 1E6 TCR-positive 5KC T-hybridoma cell line expressing each fluorochrome combination along with irrelevant T cells expressing all other fluorochrome combinations, ZsGreen-1 production was observed in only the 1E6 TCR-positive cells, while the values for all other cell lines in each culture reaction were negative (Figure 5D). These results indicate that the presence of an activated T cell in a multiplex culture reaction does not cause the activation of other cells, thus preserving the specificity of the assay without increasing false positive detection by multiplexing.

### Screening of TCR Specificity to a Large Panel of Peptides

The multiplex assay system allows us to analyze reactivity to a large number of antigens. Truncated peptide libraries consist of peptide pools, each containing mixtures of several lengths of peptides ending at the same position on a protein (Figure 6A and Supplementary Table S3). They allow for testing of reactivity to all possible epitopes present in the protein, but require numerous examinations. Taking advantage of the multiplex assay system, we analyzed the 1E6 TCR T-hybridoma cells with truncated (i.e., 8- to 11-mers) peptide libraries generated from preproinsulin. Two independent experiments that examined 1E6 TCR T-hybridoma cells expressing LSSmOrange and tdTomato detected reactivity to four consecutive peptide pools containing peptides across preproinsulin positions 12 through 25 with a peak response to the pools containing the cognate peptide PPI:15-23/PPI:15-24 (Figures 6B,C). When multiplexed with seven other irrelevant TCR-positive T-hybridoma cells, the same four peptide pools elicited a response from the 1E6 TCR (Figure 6D). Furthermore, 1E6 TCR-positive T-hybridoma cells expressing a different fluorochrome combination, Ametrine and E2-Crimson, responded to the same four peptide pools with a peak response to the same pools (Figure 6E). These results demonstrate that the multiplexed assay system provides efficient screening ability with excellent reproducibility.

### Identification of Preferred Amino Acid Motifs Using Combinatorial Peptide Libraries

Given the evidence that individual TCRs are capable of recognizing over a million epitopes (41), comprehensive analyses to identify T cell antigen specificity in a high throughput manner is desirable. Analysis with CPLs allows a global examination to identify preferred amino acid motifs in peptides that are recognized by a given TCR. CPLs consist of hundreds of peptide pools, each containing billions of peptides with a fixed amino acid residue at each position and randomized amino acids in remaining positions (Supplementary Table S4). While CPL analysis is a powerful tool for TCR antigen discovery, the dilution of the cognate peptides within each pool may limit the T cell response, and thus sensitivity of T cells to peptide stimulation is essential for precise identification of preferable amino acids. We therefore assessed whether the multiplex assay using the NFAT-based reporter system is sufficient to identify preferred amino acid motifs using a CPL.

We first examined the 1E6 T-hybridoma cell line alone with the decamer CPL in the presence of APCs that solely express the cognate HLA, i.e., A^∗^02:01. Strong responses (i.e., high ZsGreen-1 positivity) to peptide pools with particular amino acids at particular positions were reproducibly detected in two independent experiments (Figures 7A,B). The preferred amino acid residues at each position determined by these assays were identical to those previously reported by Wooldridge et al., who tested the original 1E6 T cell clone (41) using a MIP-1b ELISA readout. This demonstrates that the NFAT-based reporter system is as sensitive as studying original T cell clones for CPL analysis and provides an excellent categorization of responses. As previously described (41), amino acid recognition in N- and C-terminal positions is promiscuous, whereas it is stringent in the central P4-P6 positions corresponding to the TCR contact region. Consistent with this observation, our CPL analyses identified glycine (G) at P4, proline (P) at P5, and aspartic acid (D) at P6, which were identical to the cognate PPI:15-24 peptide sequence (Figure 7, marked by blue boxes). Furthermore, two more independent CPL analyses of 1E6 TCR-positive T-hybridomas expressing two different fluorochrome combinations that were cultured with seven irrelevant TCR-positive T cells (Figures 7C,D) were able to replicate the results provided by the examinations that solely tested this TCR (Figures 7A,B). Thus, the NFAT-based multiplex assay system can be utilized for CPL analysis without sacrificing assay sensitivity, and thereby facilitating antigen discovery studies with limited time and costs for screening a number of TCRs.

## Discussion

Nuclear factor of activated T cells-based reporter systems have been quite useful for identification and isolation of T cells that are activated by a stimulus, including antigens (24–28). Taking advantage of the NFAT-based reporter system, in this report we established an assay method that allows for the testing of antigen reactivity of multiple TCRs concurrently by flow cytometry. After assessment of several NFAT-based reporter constructs, we selected the one containing the fluorescent reporter protein ZsGreen-1, the expression of which is driven by recognition of eight repeats of the NFAT binding motif with a TATA box. The optimized multiplex assay is as sensitive to TCR response to antigen stimulation as examination of each single TCR, and ZsGreen-1 expression by activated cells is limited to those expressing antigen-specific TCRs, thus preserving specificity of the assay. We demonstrated that this multiplex assay system can be utilized for reagent- and effort-demanding analyses such as examining responses to a large panel of peptides and determining preferred amino acid residues of antigenic peptides using a CPL without sacrificing sensitivity and specificity.

TCRs consist of alpha and beta chains, the genes of which are located in different chromosomes. Therefore, identification of paired alpha and beta chain sequences from each single cell is essential to study antigen specificity of TCRs. Recent advancements in sequencing technologies now allow for high-throughput analysis of gene expression in each cell and are thereby capable of identifying TCR paired alpha and beta chain sequences. This technology has made enormous progress in immune-receptor analysis (1, 42). In conjunction with this advancement, there is an urgent need to develop methods to identify antigen specificity of the TCR clonotypes determined by high-throughput single cell sequencing. One approach is to computationally predict peptide motifs recognized by a TCR using machine-learning of big data (43–46). The major advantage of in-silico analysis is the capability to analyze an astronomical number of TCRs in a short time period. Although accuracy in peptide motif prediction still needs to be improved, in-silico modeling analysis has now become a powerful tool in particular when physically co-analyzing reactivity of TCRs to peptide-MHC complexes by wet laboratory experiments. Thus, the need for functional antigen specificity analysis is increasing.

To ascertain antigen specificity of TCRs identified by single cell sequencing, T cells are engineered to express TCRs of interest by retroviral or lentiviral gene transduction systems (47–49) and are used to test reactivity to peptide-MHC complexes. Simultaneous examinations of multiple TCRs in a single well saves both time and reagents, which is beneficial when numerous TCRs and antigens are examined. Furthermore, a multiplexed approach is optimal when limited amounts of antigen, such as tissue lysates from patients, are available. Thus, multiplex assays are preferred if assay sensitivity and specificity are sufficiently preserved, and indeed a number of multiplex immunoassays have been developed to detect target molecules such as autoantibodies and cytokines (50–52). We used various fluorescent proteins to identify cell lines, and combinations of two fluorochromes clearly distinguish each cell line. Improvements and new developments in flow cytometry along with the discovery of additional fluorescent proteins will lead to increasing multiplexity in the future, and the studies in this report provide proof-of-principle for the feasibility of multiplexing. Importantly, production of the NFAT-driven reporter protein was tightly restricted to only cells that express antigen-responsive TCRs, and bystander activation of non-specific T cells possibly resulted from the co-culture with activated T cells was ruled out. Thus, assay specificity was well preserved by multiplexing.

T-hybridoma cells expressing a particular combination of fluorochromes (ametrine and mCherry) tended to have a higher proportion of cells expressing the NFAT-driven reporter protein (ZsGreen-1) in the unstimulated condition compared to cells expressing other fluorochromes. This could potentially result in impaired assay sensitivity due to small differences in frequencies of activated (i.e., NFAT-driven reporter protein-positive) cells between the stimulated and unstimulated conditions. However, the lowest peptide concentration that can induce a detectable level of response was unchanged across all cell lines expressing different fluorescence identifiers, and thus expression of fluorochrome identifiers did not reduce sensitivity to antigen stimulation of the TCR analyzed in this study. When multiplexed, frequency of reporter protein-positive cells in the stimulated condition were slightly reduced compared to that of cells that were examined separately. This could be due to the presence of T-hybridoma cells expressing non-specific TCRs that may physically interfere with the interaction between APCs and T cells expressing specific TCRs. However, the sensitivity of detecting activated cells was unchanged whether or not cells were multiplexed. Thus, assay sensitivity was preserved when T-hybridoma cells express additional fluorescent proteins as identifiers and are multiplexed to test for response to antigen stimulation. However, it should be noted that these multiplexed analyses were performed using TCRs derived from CD8 T cells, and it may be necessary to evaluate assay sensitivity for analysis of TCRs that are derived from CD4 T cells in the future.

Improving assay sensitivity is one mission that needs continuous efforts for the future. It is known that T-hybridomas and transductants are typically less sensitive than primary T cells and clones. Indeed, the 5KC fluorescent reporter cells described in this manuscript respond to antigen with nearly ten times less sensitivity than traditional T cell clones expressing the 1E6 TCR (30). While responsiveness detected in a number of previously reported NFAT-based reporter systems vary by individual cell lines (24, 27, 53, 54), our 5KC T-hybridoma cell lines show comparable responses to stimulation despite expressing autoreactive TCRs, which typically have low affinity to peptide-MHC complexes. In order to improve the response of our T hybridoma cells to antigens, we previously introduced human CD4 with two amino acid substitutions that augment binding affinity between CD4 and MHC class II molecules, which resulted in substantial increase of T-hybridoma response to antigen (21). Given the results in this report that addition of anti-CD28 antibody improved assay sensitivity without the elevation of reactivity by unstimulated T cells, manipulating T-hybridoma cells and APCs to increase expression of costimulatory molecules such as CD28 and CD80, respectively, may facilitate greater T cell response. Another strategy to improve sensitivity in this assay system could be to use brighter fluorescent proteins as a reporter. In this report, we chose ZsGreen-1 because it does not show obvious toxicity in 5KC T-hybridoma cells while retaining sufficient brightness (55, 56). However, the newer generation of green fluorescent proteins such as superfolder GFP (sfGFP), which is extremely bright and achieves rapid and precise folding, thereby decreasing cell toxicity (57, 58), is worth considering as a fluorescent reporter protein that could improve sensitivity.

In conclusion, we established an efficient multiplex assay to test antigen specificity of T cells using a fluorescent NFAT reporter-based system that allows for simultaneous analysis of up to eight TCRs for response to antigen stimulation without reducing specificity and sensitivity. The assay system will be particularly useful for comprehensive and broad antigen specificity analysis of TCR sequences determined from single cell sequencing technologies.

## Data Availability Statement

The datasets generated for this study are available on request to the corresponding author.

## Author Contributions

SM, AM, and MN designed assay development and experiments. SM, ZZ, LL, AMA, AKA, and JF conducted the experiments. SM, KC, AM, and MN analyzed the data. MP and RM provided the essential information and materials. SM, KC, and MN wrote the manuscript. All authors reviewed and edited the manuscript.

## Conflict of Interest

The authors declare that the research was conducted in the absence of any commercial or financial relationships that could be construed as a potential conflict of interest.

## Footnotes

1 http://www.ebi.ac.uk/ipd/imgt/hla/

2 http://n2t.net/addgene:85969

3 https://www.fpbase.org/protein/mametrine/

4 https://www.fpbase.org/protein/lssmorange/

5 https://www.fpbase.org/protein/mtagbfp2/

6 https://www.fpbase.org/protein/tdtomato/

7 https://www.fpbase.org/protein/e2-crimson/

8 https://www.fpbase.org/protein/mcherry/

## Abbreviations

CPL: combinatorial peptide library
Insulin B:9-23: insulin B chain 9-23
miniCMV: minimal CMV
MoMuLV: 3 ′ Moloney murine leukemia virus
muhCD4: mutated human CD4
NFAT: nuclear factor of activated T cells
PPI:15-24: preproinsulin 15-24
TCR: T cell receptor.

## References

[1] Guo XZ Dash P Calverley M Tomchuck S Dallas MH Thomas PG. Rapid cloning, expression, and functional characterization of paired alphabeta and gammadelta T-cell receptor chains from single-cell analysis. *Mol Ther Methods Clin Dev.* (2016) 3:15054. 10.1038/mtm.2015.54 PMC4729322 26858965

[2] Atkinson MA von Herrath M Powers AC Clare-Salzler M. Current concepts on the pathogenesis of type 1 diabetes–considerations for attempts to prevent and reverse the disease. *Diabetes Care.* (2015) 38:979–88. 10.2337/dc15-0144 25998290 PMC4439528

[3] Michels AW Landry LG McDaniel KA Yu L Campbell-Thompson M Kwok WW Islet-derived CD4 T cells targeting proinsulin in human autoimmune diabetes. *Diabetes.* (2017) 66:722–34. 10.2337/db16-1025 27920090 PMC5319719

[4] Bassani-Sternberg M Coukos G. Mass spectrometry-based antigen discovery for cancer immunotherapy. *Curr Opin Immunol.* (2016) 41:9–17. 10.1016/j.coi.2016.04.005 27155075

[5] Spence A Purtha W Tam J Dong S Kim Y Ju CH Revealing the specificity of regulatory T cells in murine autoimmune diabetes. *Proc Natl Acad Sci USA.* (2018) 115:5265–70. 10.1073/pnas.1715590115 29712852 PMC5960284

[6] Pearson RM Podojil JR Shea LD King NJC Miller SD Getts DR. Overcoming challenges in treating autoimmuntity: development of tolerogenic immune-modifying nanoparticles. *Nanomed Nanotechnol Biol Med.* (2019) 18:282–91. 10.1016/j.nano.2018.10.001 PMC6830541 30352312

[7] Umeshappa CS Singha S Blanco J Shao K Nanjundappa RH Yamanouchi J Suppression of a broad spectrum of liver autoimmune pathologies by single peptide-MHC-based nanomedicines. *Nat Commun.* (2019) 10:2150. 10.1038/s41467-019-09893-5 PMC6517389 31089130

[8] Singha S Shao K Yang Y Clemente-Casares X Sole P Clemente A Peptide-MHC-based nanomedicines for autoimmunity function as T-cell receptor microclustering devices. *Nat Nanotechnol.* (2017) 12:701–10. 10.1038/nnano.2017.56 28436959

[9] Jamison BL Neef T Goodspeed A Bradley B Baker RL Miller SD Nanoparticles containing an insulin-ChgA hybrid peptide protect from transfer of autoimmune diabetes by shifting the balance between effector T cells and regulatory T cells. *J Immunol.* (2019) 203:48–57. 10.4049/jimmunol.1900127 31109955 PMC6581587

[10] Smarr CB Yap WT Neef TP Pearson RM Hunter ZN Ifergan I Biodegradable antigen-associated PLG nanoparticles tolerize Th2-mediated allergic airway inflammation pre- and postsensitization. *Proc Natl Acad Sci USA.* (2016) 113:5059–64. 10.1073/pnas.1505782113 27091976 PMC4983813

[11] Zhang L Crawford F Yu L Michels A Nakayama M Davidson HW Monoclonal antibody blocking the recognition of an insulin peptide-MHC complex modulates type 1 diabetes. *Proc Natl Acad Sci USA.* (2014) 111:2656–61. 10.1073/pnas.1323436111 24550292 PMC3932899

[12] Ostrov DA Alkanani A McDaniel KA Case S Baschal EE Pyle L Methyldopa blocks MHC class II binding to disease-specific antigens in autoimmune diabetes. *J Clin Investigat.* (2018) 128:1888–902. 10.1172/JCI97739 PMC5919818 29438107

[13] Holzinger A Barden M Abken H. The growing world of CAR T cell trials: a systematic review. *Cancer Immunol Immunother CII.* (2016) 65:1433–50. 10.1007/s00262-016-1895-5 27613725 PMC11029082

[14] Bercovici N Duffour MT Agrawal S Salcedo M Abastado JP. New methods for assessing T-cell responses. *Clin Diagnos Lab Immunol.* (2000) 7(6):859–64. 10.1128/cdli.7.6.859-864.2000 PMC95974 11063487

[15] Karlsson AC Martin JN Younger SR Bredt BM Epling L Ronquillo R Comparison of the ELISPOT and cytokine flow cytometry assays for the enumeration of antigen-specific T cells. *J Immunol Methods.* (2003) 283:141–53. 10.1016/j.jim.2003.09.001 14659906

[16] Malyguine AM Strobl S Dunham K Shurin MR Sayers TJ. ELISPOT assay for monitoring cytotoxic T lymphocytes (CTL) activity in cancer vaccine clinical trials. *Cells.* (2012) 1:111–26. 10.3390/cells1020111 24710418 PMC3901085

[17] Parish CR Glidden MH Quah BJ Warren HS. Use of the intracellular fluorescent dye CFSE to monitor lymphocyte migration and proliferation. *Curr Prot Immunol.* (2009) Chapter 4:Unit4.9. 10.1002/0471142735.im0409s84 . 19235770

[18] Mannering SI Wong FS Durinovic-Bello I Brooks-Worrell B Tree TI Cilio CM Current approaches to measuring human islet-antigen specific T cell function in type 1 diabetes. *Clin Exp Immunol.* (2010) 162:197–209. 10.1111/j.1365-2249.2010.04237.x 20846160 PMC2996587

[19] Ciantar JP Mannering SI. An improved method for growing and analysing human antigen-specific CD4+ T-cell clones. *Diabet Metab Res Rev.* (2011) 27:906–12. 10.1002/dmrr.1271 22069283

[20] Fitch FW. T- cell clones and T-cell receptors. *Microbiol Rev.* (1986) 50:50–69. 10.1128/mmbr.50.1.50-69.1986 3083221 PMC373053

[21] Williams T Krovi HS Landry LG Crawford F Jin N Hohenstein A Development of T cell lines sensitive to antigen stimulation. *J Immunol Methods.* (2018) 462:65–73. 10.1016/j.jim.2018.08.011 30165064 PMC8006528

[22] White J Pullen A Choi K Marrack P Kappler JW. Antigen recognition properties of mutant V beta 3+ T cell receptors are consistent with an immunoglobulin-like structure for the receptor. *J Exp Med.* (1993) 177:119–25. 10.1084/jem.177.1.119 8380294 PMC2190864

[23] Canaday DH. Production of CD4(+) and CD8(+) T cell hybridomas. *Methods Mol Biol (Clifton N J).* (2013) 960:297–307. 10.1007/978-1-62703-218-6_22 PMC4552323 23329495

[24] Jutz S Leitner J Schmetterer K Doel-Perez I Majdic O Grabmeier-Pfistershammer K Assessment of costimulation and coinhibition in a triple parameter T cell reporter line: simultaneous measurement of NF-kappaB, NFAT and AP-1. *J Immunol Methods.* (2016) 430:10–20. 10.1016/j.jim.2016.01.007 26780292

[25] Ise W Kohyama M Nutsch KM Lee HM Suri A Unanue ER 4 suppresses the pathogenicity of self antigen-specific T cells by cell-intrinsic and cell-extrinsic mechanisms. *Nat Immunol.* (2010) 11:129–35. 10.1038/ni.1835 20037585 PMC3235641

[26] Zhu H Yang J Murphy TL Ouyang W Wagner F Saparov A Unexpected characteristics of the IFN-gamma reporters in nontransformed T cells. *J Immunol.* (2001) 167:855–65. 10.4049/jimmunol.167.2.855 11441092

[27] Ponomarev V Doubrovin M Lyddane C Beresten T Balatoni J Bornman W Imaging TCR-dependent NFAT-mediated T-cell activation with positron emission tomography in vivo. *Neoplasia (New York, NY).* (2001) 3:480–8. 10.1038/sj.neo.7900204 PMC1506564 11774030

[28] Hooijberg E Bakker AQ Ruizendaal JJ Spits H. NFAT- controlled expression of GFP permits visualization and isolation of antigen-stimulated primary human T cells. *Blood.* (2000) 96:459–66. 10.1182/blood.V96.2.459 10887106

[29] Wang XX Li Y Yin Y Mo M Wang Q Gao W Affinity maturation of human CD4 by yeast surface display and crystal structure of a CD4-HLA-DR1 complex. *Proc Natl Acad Sci USA.* (2011) 108:15960–5. 10.1073/pnas.1109438108 21900604 PMC3179091

[30] Skowera A Ellis RJ Varela-Calvino R Arif S Huang GC Van-Krinks C CTLs are targeted to kill beta cells in patients with type 1 diabetes through recognition of a glucose-regulated preproinsulin epitope. *J Clin Invest.* (2008) 118:3390–402. 10.1172/JCI35449 18802479 PMC2542849

[31] Scrima M De Marco C De Vita F Fabiani F Franco R Pirozzi G The nonreceptor-type tyrosine phosphatase PTPN13 is a tumor suppressor gene in non-small cell lung cancer. *Am J Pathol.* (2012) 180:1202–14. 10.1016/j.ajpath.2011.11.038 22245727

[32] Holst J Szymczak-Workman AL Vignali KM Burton AR Workman CJ Vignali DA. Generation of T-cell receptor retrogenic mice. *Nat Protoc.* (2006) 1:406–17. 10.1038/nprot.2006.61 17406263

[33] Bettini ML Bettini M Nakayama M Guy CS Vignali DA. Generation of T cell receptor-retrogenic mice: improved retroviral-mediated stem cell gene transfer. *Nat Protoc.* (2013) 8:1837–40. 10.1038/nprot.2013.111 24008379 PMC3832243

[34] Szymczak AL Workman CJ Wang Y Vignali KM Dilioglou S Vanin EF Correction of multi-gene deficiency in vivo using a single ‘self-cleaving’ 2A peptide-based retroviral vector. *Nat Biotechnol.* (2004) 22:589–94. 10.1038/nbt957 15064769

[35] Adamson B Norman TM Jost M Cho MY Nunez JK Chen Y A multiplexed single-cell CRISPR screening platform enables systematic dissection of the unfolded protein response. *Cell.* (2016) 167:1867–82.e21. 10.1016/j.cell.2016.11.048 27984733 PMC5315571

[36] Campbell-Thompson M Wasserfall C Kaddis J Albanese-O’Neill A Staeva T Nierras C Network for pancreatic organ donors with diabetes (nPOD): developing a tissue biobank for type 1 diabetes. *Diab Metab Res Rev.* (2012) 28:608–17. 10.1002/dmrr.2316 PMC3456997 22585677

[37] Pugliese A Yang M Kusmarteva I Heiple T Vendrame F Wasserfall C The juvenile diabetes research foundation network for pancreatic organ donors with diabetes (nPOD) program: goals, operational model and emerging findings. *Pediatric Diabetes.* (2014) 15:1–9. 10.1111/pedi.12097 PMC4282794 24325575

[38] Hemmer B Gran B Zhao Y Marques A Pascal J Tzou A Identification of candidate T-cell epitopes and molecular mimics in chronic Lyme disease. *Nat Med.* (1999) 5:1375–82. 10.1038/70946 10581079

[39] Zhao Y Gran B Pinilla C Markovic-Plese S Hemmer B Tzou A Combinatorial peptide libraries and biometric score matrices permit the quantitative analysis of specific and degenerate interactions between clonotypic TCR and MHC peptide ligands. *J Immunol.* (2001) 167:2130–41. 10.4049/jimmunol.167.4.2130 11489997

[40] Foecking MK Hofstetter H. Powerful and versatile enhancer-promoter unit for mammalian expression vectors. *Gene.* (1986) 45:101–5. 10.1016/0378-1119(86)90137-x 3023199

[41] Wooldridge L Ekeruche-Makinde J van den Berg HA Skowera A Miles JJ Tan MP Single autoimmune T cell receptor recognizes more than a million different peptides. *J Biol Chem.* (2012) 287:1168–77. 10.1074/jbc.M111.289488 22102287 PMC3256900

[42] Friedensohn S Khan TA Reddy ST. Advanced methodologies in high-throughput sequencing of immune repertoires. *Trends Biotechnol.* (2017) 35:203–14. 10.1016/j.tibtech.2016.09.010 28341036

[43] Dash P Fiore-Gartland AJ Hertz T Wang GC Sharma S Souquette A Quantifiable predictive features define epitope-specific T cell receptor repertoires. *Nature.* (2017) 547:89–93. 10.1038/nature22383 28636592 PMC5616171

[44] Glanville J Huang H Nau A Hatton O Wagar LE Rubelt F Identifying specificity groups in the T cell receptor repertoire. *Nature.* (2017) 547:94–8. 10.1038/nature22976 28636589 PMC5794212

[45] Pogorelyy MV Shugay MA. Framework for annotation of antigen specificities in high-throughput T-cell repertoire sequencing studies. *Front Immunol.* (2019) 10:2159. 10.3389/fimmu.2019.02159 PMC6775185 31616409

[46] Jacobsen LM Posgai A Seay HR Haller MJ Brusko TM. T- cell receptor profiling in type 1 diabetes. *Curr Diab Rep.* (2017) 17:118. 10.1007/s11892-017-0946-4 PMC5636870 29022222

[47] Yeh WI Seay HR Newby B Posgai AL Moniz FB Michels A Avidity and bystander suppressive capacity of human regulatory T cells expressing de novo autoreactive T-cell receptors in type 1 diabetes. *Front Immunol.* (2017) 8:1313. 10.3389/fimmu.2017.01313 PMC5662552 29123516

[48] Perro M Tsang J Xue SA Escors D Cesco-Gaspere M Pospori C Generation of multi-functional antigen-specific human T-cells by lentiviral TCR gene transfer. *Gene Ther.* (2010) 17:721–32. 10.1038/gt.2010.4 20164855

[49] Jones S Peng PD Yang S Hsu C Cohen CJ Zhao Y Lentiviral vector design for optimal T cell receptor gene expression in the transduction of peripheral blood lymphocytes and tumor-infiltrating lymphocytes. *Hum Gene Ther.* (2009) 20:630–40. 10.1089/hum.2008.048 19265475 PMC2828626

[50] Zhao Z Miao D Michels A Steck A Dong F Rewers M Multiplex assay combining insulin, GAD, IA-2 and transglutaminase autoantibodies to facilitate screening for pre-type 1 diabetes and celiac disease. *J Immunol Methods.* (2016) 430:28–32. 10.1016/j.jim.2016.01.011 26809048 PMC5851776

[51] Gu Y Zhao Z Waugh K Miao D Jia X Cheng J High-throughput multiplexed autoantibody detection to screen type 1 diabetes and multiple autoimmune diseases simultaneously. *EBio Medicine.* (2019) 47:365–72. 10.1016/j.ebiom.2019.08.036 PMC6796526 31447394

[52] Paquet J Goebel JC Delaunay C Pinzano A Grossin L Cournil-Henrionnet C Cytokines profiling by multiplex analysis in experimental arthritis: which pathophysiological relevance for articular versus systemic mediators? *Arthr Res Ther.* (2012) 14:R60. 10.1186/ar3774 PMC3446427 22414623

[53] Albrecht B D’Souza CD Ding W Tridandapani S Coggeshall KM Lairmore MD. Activation of nuclear factor of activated T cells by human T-lymphotropic virus type 1 accessory protein p12(I). *J Virol.* (2002) 76:3493–501. 10.1128/jvi.76.7.3493-3501.2002 11884573 PMC136046

[54] Rosskopf S Leitner J Paster W Morton LT Hagedoorn RS Steinberger P Jurkat 76 based triple parameter reporter system to evaluate TCR functions and adoptive T cell strategies. *Oncotarget.* (2018) 9:17608–19. 10.18632/oncotarget.24807 29707134 PMC5915142

[55] Bell P Vandenberghe LH Wu D Johnston J Limberis M Wilson JMA. comparative analysis of novel fluorescent proteins as reporters for gene transfer studies. *J Histochem Cytochem Offic J Histochem Soc.* (2007) 55:931–9. 10.1369/jhc.7A7180.2007 17510373

[56] Ilagan RP Rhoades E Gruber DF Kao HT Pieribone VA Regan L. A new bright green-emitting fluorescent protein–engineered monomeric and dimeric forms. *FEBS J.* (2010) 277:1967–78. 10.1111/j.1742-4658.2010.07618.x 20345907 PMC2855763

[57] Pedelacq JD Cabantous S Tran T Terwilliger TC Waldo GS. Engineering and characterization of a superfolder green fluorescent protein. *Nat Biotechnol.* (2006) 24:79–88. 10.1038/nbt1172 16369541

[58] Lambert TJ. FPbase: a community-editable fluorescent protein database. *Nat Methods.* (2019) 16:277–8. 10.1038/s41592-019-0352-8 30886412

