# Supplementary material for: Multiplex T Cell Stimulation Assay Utilizing a T Cell Activation Reporter-Based Detection System
Source: Front Immunol. 2020 Apr 9;11:633. doi: 10.3389/fimmu.2020.00633 (PMC7160884; doi:10.3389/fimmu.2020.00633)
Supplement: Supplementary file 1 [file Data_Sheet_1.zip › Supplementary Document.DOCX]

**Supplementary Document 1**

**V51-mu4 (pSIREN-muhCD4)**

Sequence

gatatcccaacattaccgccatgttgacattgattattgactagttattaatagtaatcaattacggggtcattagttcatagcccatatatggagttccgcgttacataacttacggtaaatggcccgcctggctgaccgcccaacgacccccgcccattgacgtcaataatgacgtatgttcccatagtaacgccaatagggactttccattgacgtcaatgggtggagtatttacggtaaactgcccacttggcagtacatcaagtgtatcatatgccaagtacgccccctattgacgtcaatgacggtaaatggcccgcctggcattatgcccagtacatgaccttatgggactttcctacttggcagtacatctacgtattagtcatcgctattaccatggtgatgcggttttggcagtacatcaatgggcgtggatagcggtttgactcacggggatttccaagtctccaccccattgacgtcaatgggagtttgttttggcaccaaaatcaacgggactttccaaaatgtcgtaacaactccgccccattgacgcaaatgggcggtaggcgtgtacggtgggaggtctatataagcagagctcaataaaagagcccacaacccctcactcggcgcgccagtcttccgatagactgcgtcgcccgggtacccgtattcccaataaagcctcttgctgtttgcatccgaatcgtggtctcgctgttccttgggagggtctcctctgagtgattgactacccacgacgggggtctttcatttgggggctcgtccgggatttggagacccctgcccagggaccaccgacccaccaccgggaggtaagctggccagcaacttatctgtgtctgtccgattgtctagtgtctatgtttgatgttatgcgcctgcgtctgtactagttagctaactagctctgtatctggcggacccgtggtggaactgacgagttctgaacacccggccgcaaccctgggagacgtcccagggactttgggggccgtttttgtggcccgacctgaggaagggagtcgatgtggaatccgaccccgtcaggatatgtggttctggtaggagacgagaacctaaaacagttcccgcctccgtctgaatttttgctttcggtttggaaccgaagccgcgcgtcttgtctgctgcagcgctgcagcatcgttctgtgttgtctctgtctgactgtgtttctgtatttgtctgaaaattagggccagactgttaccactcccttaagtttgaccttaggtcactggaaagatgtcgagcggatcgctcacaaccagtcggtagatgtcaagaagagacgttgggttaccttctgctctgcagaatggccaacctttaacgtcggatggccgcgagacggcacctttaaccgagacctcatcacccaggttaagatcaaggtcttttcacctggcccgcatggacacccagaccaggtcccctacatcgtgacctgggaagccttggcttttgacccccctccctgggtcaagccctttgtacaccctaagcctccgcctcctcttcctccatccgccccgtctctcccccttgaacctcctcgttcgaccccgcctcgatcctccctttatccagccctcactccttctctaggcgccggaattgaagatctctcgaggttaacgaattctaccgggtaggggaggcgcttttcccaaggcagtctggagcatgcgctttagcagccccgctgggcacttggcgctacacaagtggcctctggcctcgcacacattccacatccaccggtaggcgccaaccggctccgttctttggtggccccttcgcgccaccttctactcctcccctagtcaggaagttcccccccgccccgcagctcgcgtcgtgcaggacgtgacaaatggaagtagcacgtctcactagtctcgtgcagatggacagcaccgctgagcaatggaagcgggtaggcctttggggcagcggccaatagcagctttgctccttcgctttctgggctcagaggctgggaaggggtgggtccgggggcgggctcaggggcgggctcaggggcggggcgggcgcccgaaggtcctccggaggcccggcattctgcacgcttcaaaagcgcacgtctgccgcgctgttctcctcttcctcatctccgggcctttcgacctgcatcccgccaccATGAACCGGGGAGTCCCTTTTAGGCACTTGCTTCTGGTGCTGCAACTGGCGCTCCTCCCAGCAGCCACTCAGGGAAAGAAAGTGGTGCTGGGCAAAAAAGGGGATACAGTGGAACTGACCTGTACAGCTTCCCAGAAGAAGAGCATACAATTCCACTGGAAAAACTCCAACCAGATAAAGATTCTGGGAAATTACGGCTCCTTCTTATGGAAAGGTCCATCCAAGCTGAATGATCGCGCTGACTCAAGAAGAAGCCTTTGGGACCAAGGAAACTTCCCCCTGATCATCAAGAATCTTAAGATAGAAGACTCAGATACTTACATCTGTGAAGTGGAGGACCAGAAGGAGGAGGTGCAATTGCTAGTGTTCGGATTGACTGCCAACTCTGACACCCACCTGCTTCAGGGGCAGAGCCTGACCCTGACCTTGGAGAGCCCCCCTGGTAGTAGCCCCTCAGTGCAATGTAGGAGTCCAAGGGGTAAAAACATACAGGGGGGGAAGACCCTCTCCGTGTCTCAGCTGGAGCTCCAGGATAGTGGCACCTGGACATGCACTGTCTTGCAGAACCAGAAGAAGGTGGAGTTCAAAATAGACATCGTGGTGCTAGCTTTCCAGAAGGCCTCCAGCATAGTCTATAAGAAAGAGGGGGAACAGGTGGAGTTCTCCTTCCCACTCGCCTTTACAGTTGAAAAGCTGACGGGCAGTGGCGAGCTGTGGTGGCAGGCGGAGAGGGCTTCCTCCTCCAAGTCTTGGATCACCTTTGACCTGAAGAACAAGGAAGTGTCTGTAAAACGGGTTACCCAGGACCCTAAGCTCCAGATGGGCAAGAAGCTCCCGCTCCACCTCACCCTGCCCCAGGCCTTGCCTCAGTATGCTGGCTCTGGAAACCTCACCCTGGCCCTTGAAGCGAAAACAGGAAAGTTGCACCAGGAAGTGAACCTGGTGGTGATGAGAGCCACTCAGCTCCAGAAAAATTTGACCTGTGAGGTGTGGGGACCCACCTCCCCTAAGCTGATGCTGAGCTTGAAACTGGAGAACAAGGAGGCAAAGGTCTCGAAGCGGGAGAAGGCGGTGTGGGTGCTGAACCCTGAGGCGGGGATGTGGCAGTGTCTGCTGAGTGACTCGGGACAGGTCCTGCTGGAATCCAACATCAAGGTTCTGCCCACATGGTCGACCCCGGTGCAGCCAATGGCCCTGATTGTGCTGGGGGGCGTCGCCGGCCTCCTGCTGTTCATTGGGCTAGGCATCTTCTTCTGTGTCAGGTGCCGGCACCGAAGGCGCCAAGCAGAGCGGATGTCTCAGATCAAGAGACTCCTCAGTGAGAAGAAGACCTGCCAGTGCCCTCACCGGTTTCAGAAGACATGTAGCCCCATTTGAaagcttcaccagctgaagcctatagagtacgagccatagataaaataaaagattttatttagtctccagaaaaaggggggaatgaaagaccccacctgtaggtttggcaagctagcttaagtaacgccattttgcaaggcatggaaaaatacataactgagaatagagaagttcagatcaaggtcaggaacagatggaacagggtcgaccctagagaaccatcagatgtttccagggtgccccaaggacctgaaatgaccctgtgccttatttgaactaaccaatcagttcgcttctcgcttctgttcgcgcgcttctgctccccgagctcaataaaagagcccacaacccctcactcggggcgccagtcctccgattgactgagtcgcccgggtacccgtgtatccaataaaccctcttgcagttgcatccgacttgtggtctcgctgttccttgggagggtctcctctgagtgattgactacccgtcagcgggggtctttcatttgggggctcgtccgggatcgggagacccctgcccagggaccaccgacccaccaccgggaggtaagctggctgcctcgcgcgtttcggtgatgacggtgaaaacctctgacatgtgagcaaaaggccagcaaaaggccaggaaccgtaaaaaggccgcgttgctggcgtttttccataggctccgcccccctgacgagcatcacaaaaatcgacgctcaagtcagaggtggcgaaacccgacaggactataaagataccaggcgtttccccctggaagctccctcgtgcgctctcctgttccgaccctgccgcttaccggatacctgtccgcctttctcccttcgggaagcgtggcgctttctcatagctcacgctgtaggtatctcagttcggtgtaggtcgttcgctccaagctgggctgtgtgcacgaaccccccgttcagcccgaccgctgcgccttatccggtaactatcgtcttgagtccaacccggtaagacacgacttatcgccactggcagcagccactggtaacaggattagcagagcgaggtatgtaggcggtgctacagagttcttgaagtggtggcctaactacggctacactagaagaacagtatttggtatctgcgctctgctgaagccagttaccttcggaaaaagagttggtagctcttgatccggcaaacaaaccaccgctggtagcggtggtttttttgtttgcaagcagcagattacgcgcagaaaaaaaggatctcaagaagatcctttgatcttttctacggggtctgacgctcagtggaacgaaaactcacgttaagggattttggtcatgagattatcaaaaaggatcttcacctagatccttttaaattaaaaatgaagttttaaatcaatctaaagtatatatgagtaaacttggtctgacagttaccaatgcttaatcagtgaggcacctatctcagcgatctgtctatttcgttcatccatagttgcctgactccccgtcgtgtagataactacgatacgggagggcttaccatctggccccagtgctgcaatgataccgcgagacccacgctcaccggctccagatttatcagcaataaaccagccagccggaagggccgagcgcagaagtggtcctgcaactttatccgcctccatccagtctattaattgttgccgggaagctagagtaagtagttcgccagttaatagtttgcgcaacgttgttgccattgctacaggcatcgtggtgtcacgctcgtcgtttggtatggcttcattcagctccggttcccaacgatcaaggcgagttacatgatcccccatgttgtgcaaaaaagcggttagctccttcggtcctccgatcgttgtcagaagtaagttggccgcagtgttatcactcatggttatggcagcactgcataattctcttactgtcatgccatccgtaagatgcttttctgtgactggtgagtactcaaccaagtcattctgagaatagtgtatgcggcgaccgagttgctcttgcccggcgtcaatacgggataataccgcgccacatagcagaactttaaaagtgctcatcattggaaaacgttcttcggggcgaaaactctcaaggatcttaccgctgttgagatccagttcgatgtaacccactcgtgcacccaactgatcttcagcatcttttactttcaccagcgtttctgggtgagcaaaaacaggaaggcaaaatgccgcaaaaaagggaataagggcgacacggaaatgttgaatactcatactcttcctttttcaatattattgaagcatttatcagggttattgtctcatgagcggatacatatttgaatgtatttagaaaaataaacaaataggggttccgcgcacatttccccgaaaagtgccacctgacgtctaagaaaccattattatcatgacattaacctataaaaataggcgtatcacgaggccctttcgtctcgcgcgtttcggtgatgacggtgaaaacctctgacacatgcagctcccggagacggtcacagcttgtctgtaagcggatgccgggagcagacaagcccgtcagggcgcgtcagcgggtgttggcgggtgtcggggctggcttaactatgcggcatcagagcagattgtactgagagtgcaccatatgcggtgtgaaataccgcacagatgcgtaaggagaaaataccgcatcaggcgccattcgccattcaggctgcgcaactgttgggaagggcgatcggtgcgggcctcttcgctattacgccagctggcgaaagggggatgtgctgcaaggcgattaagttgggtaacgccagggttttcccagtcacgacgttgtaaaacgacggcgcaaggaatggtgcatgcaaggagatggcgcccaacagtcccccggccacggggcctgccaccatacccacgccgaaacaagcgctcatgagcccgaagtggcgagcccgatcttccccatcggtgatgtcggcgatataggcgccagcaaccgcacctgtggcgccggtgatgccggccacgatgcgtccggcgtagaggcgattagtccaatttgttaaagacag

**V51-8 (pSIREN-hCD8)**

Sequence

gatatcccaacattaccgccatgttgacattgattattgactagttattaatagtaatcaattacggggtcattagttcatagcccatatatggagttccgcgttacataacttacggtaaatggcccgcctggctgaccgcccaacgacccccgcccattgacgtcaataatgacgtatgttcccatagtaacgccaatagggactttccattgacgtcaatgggtggagtatttacggtaaactgcccacttggcagtacatcaagtgtatcatatgccaagtacgccccctattgacgtcaatgacggtaaatggcccgcctggcattatgcccagtacatgaccttatgggactttcctacttggcagtacatctacgtattagtcatcgctattaccatggtgatgcggttttggcagtacatcaatgggcgtggatagcggtttgactcacggggatttccaagtctccaccccattgacgtcaatgggagtttgttttggcaccaaaatcaacgggactttccaaaatgtcgtaacaactccgccccattgacgcaaatgggcggtaggcgtgtacggtgggaggtctatataagcagagctcaataaaagagcccacaacccctcactcggcgcgccagtcttccgatagactgcgtcgcccgggtacccgtattcccaataaagcctcttgctgtttgcatccgaatcgtggtctcgctgttccttgggagggtctcctctgagtgattgactacccacgacgggggtctttcatttgggggctcgtccgggatttggagacccctgcccagggaccaccgacccaccaccgggaggtaagctggccagcaacttatctgtgtctgtccgattgtctagtgtctatgtttgatgttatgcgcctgcgtctgtactagttagctaactagctctgtatctggcggacccgtggtggaactgacgagttctgaacacccggccgcaaccctgggagacgtcccagggactttgggggccgtttttgtggcccgacctgaggaagggagtcgatgtggaatccgaccccgtcaggatatgtggttctggtaggagacgagaacctaaaacagttcccgcctccgtctgaatttttgctttcggtttggaaccgaagccgcgcgtcttgtctgctgcagcgctgcagcatcgttctgtgttgtctctgtctgactgtgtttctgtatttgtctgaaaattagggccagactgttaccactcccttaagtttgaccttaggtcactggaaagatgtcgagcggatcgctcacaaccagtcggtagatgtcaagaagagacgttgggttaccttctgctctgcagaatggccaacctttaacgtcggatggccgcgagacggcacctttaaccgagacctcatcacccaggttaagatcaaggtcttttcacctggcccgcatggacacccagaccaggtcccctacatcgtgacctgggaagccttggcttttgacccccctccctgggtcaagccctttgtacaccctaagcctccgcctcctcttcctccatccgccccgtctctcccccttgaacctcctcgttcgaccccgcctcgatcctccctttatccagccctcactccttctctaggcgccggaattgaagatctctcgaggttaacgaattctaccgggtaggggaggcgcttttcccaaggcagtctggagcatgcgctttagcagccccgctgggcacttggcgctacacaagtggcctctggcctcgcacacattccacatccaccggtaggcgccaaccggctccgttctttggtggccccttcgcgccaccttctactcctcccctagtcaggaagttcccccccgccccgcagctcgcgtcgtgcaggacgtgacaaatggaagtagcacgtctcactagtctcgtgcagatggacagcaccgctgagcaatggaagcgggtaggcctttggggcagcggccaatagcagctttgctccttcgctttctgggctcagaggctgggaaggggtgggtccgggggcgggctcaggggcgggctcaggggcggggcgggcgcccgaaggtcctccggaggcccggcattctgcacgcttcaaaagcgcacgtctgccgcgctgttctcctcttcctcatctccgggcctttcgacctgcatcccgccaccATGGCCTTACCAGTGACCGCCTTGCTCCTGCCGCTGGCCTTGCTGCTCCACGCCGCCAGGCCGAGCCAGTTCCGGGTGTCGCCGCTGGATCGGACCTGGAACCTGGGCGAGACAGTGGAGCTGAAGTGCCAGGTGCTGCTGTCCAACCCGACGTCGGGCTGCTCGTGGCTCTTCCAGCCGCGCGGCGCCGCCGCCAGTCCCACCTTCCTCCTATACCTCTCCCAAAACAAGCCCAAGGCGGCCGAGGGGCTGGACACCCAGCGGTTCTCGGGCAAGAGGTTGGGGGACACCTTCGTCCTCACCCTGAGCGACTTCCGCCGAGAGAACGAGGGCTACTATTTCTGCTCGGCCCTGAGCAACTCCATCATGTACTTCAGCCACTTCGTGCCGGTCTTCCTGCCAGCGAAGCCCACCACGACGCCAGCGCCGCGACCACCAACACCGGCGCCCACCATCGCGTCGCAGCCCCTGTCCCTGCGCCCAGAGGCGTGCCGGCCAGCGGCGGGGGGCGCAGTGCACACGAGGGGGCTGGACTTCGCCTGTGATATCTACATCTGGGCGCCCTTGGCCGGGACTTGTGGGGTCCTTCTCCTGTCACTGGTTATCACCCTTTACTGCAACCACAGGAACCGAAGACGTGTTTGCAAATGTCCCCGGCCTGTGGTCAAATCGGGAGACAAGCCCAGCCTTTCGGCGAGATACGTCGGCTCCGGAGCCACGAACTTCTCTCTGTTAAAGCAAGCAGGAGACGTGGAAGAAAACCCCGGTCCCATGCGGCCGCGGCTGTGGCTCCTCCTGGCCGCGCAGCTGACAGTTCTCCATGGCAACTCAGTCCTCCAGCAGACCCCTGCATACATAAAGGTGCAAACCAACAAGATGGTGATGCTGTCCTGCGAAGCTAAAATCTCCCTCAGTAACATGCGCATCTACTGGCTGAGACAGCGCCAGGCACCGAGCAGTGACAGTCACCACGAGTTCCTGGCCCTCTGGGATTCCGCAAAAGGGACTATCCACGGTGAAGAGGTGGAACAGGAGAAGATAGCTGTGTTTCGGGATGCAAGCCGGTTCATTCTCAATCTCACAAGCGTGAAGCCGGAAGACAGTGGCATCTACTTCTGCATGATCGTCGGGAGCCCCGAGCTGACCTTCGGGAAGGGAACTCAGCTGAGTGTGGTTGATTTCCTTCCCACCACTGCCCAGCCCACCAAGAAGTCCACCCTCAAGAAGAGAGTGTGCCGGTTACCCAGGCCAGAGACCCAGAAGGGCCCACTTTGTAGCCCCATCACCCTTGGCCTGCTGGTGGCTGGCATCCTGGTTCTGCTGGTTTCCCTGGGAGTGGCCATCCACCTGTGCTGCCGGCGGAGGAGAGCCCGGCTTCGTTTCATGAAACAGCCTCAAGGGGAAGGTATATCAGGAACCTTTGTCCCCCAATGCCTGCATGGATACTACAGCAATACTACAACCTCACAGAAGCTGCTTAACCCATGGATCCTGAAAACATAGaagcttcaccagctgaagcctatagagtacgagccatagataaaataaaagattttatttagtctccagaaaaaggggggaatgaaagaccccacctgtaggtttggcaagctagcttaagtaacgccattttgcaaggcatggaaaaatacataactgagaatagagaagttcagatcaaggtcaggaacagatggaacagggtcgaccctagagaaccatcagatgtttccagggtgccccaaggacctgaaatgaccctgtgccttatttgaactaaccaatcagttcgcttctcgcttctgttcgcgcgcttctgctccccgagctcaataaaagagcccacaacccctcactcggggcgccagtcctccgattgactgagtcgcccgggtacccgtgtatccaataaaccctcttgcagttgcatccgacttgtggtctcgctgttccttgggagggtctcctctgagtgattgactacccgtcagcgggggtctttcatttgggggctcgtccgggatcgggagacccctgcccagggaccaccgacccaccaccgggaggtaagctggctgcctcgcgcgtttcggtgatgacggtgaaaacctctgacatgtgagcaaaaggccagcaaaaggccaggaaccgtaaaaaggccgcgttgctggcgtttttccataggctccgcccccctgacgagcatcacaaaaatcgacgctcaagtcagaggtggcgaaacccgacaggactataaagataccaggcgtttccccctggaagctccctcgtgcgctctcctgttccgaccctgccgcttaccggatacctgtccgcctttctcccttcgggaagcgtggcgctttctcatagctcacgctgtaggtatctcagttcggtgtaggtcgttcgctccaagctgggctgtgtgcacgaaccccccgttcagcccgaccgctgcgccttatccggtaactatcgtcttgagtccaacccggtaagacacgacttatcgccactggcagcagccactggtaacaggattagcagagcgaggtatgtaggcggtgctacagagttcttgaagtggtggcctaactacggctacactagaagaacagtatttggtatctgcgctctgctgaagccagttaccttcggaaaaagagttggtagctcttgatccggcaaacaaaccaccgctggtagcggtggtttttttgtttgcaagcagcagattacgcgcagaaaaaaaggatctcaagaagatcctttgatcttttctacggggtctgacgctcagtggaacgaaaactcacgttaagggattttggtcatgagattatcaaaaaggatcttcacctagatccttttaaattaaaaatgaagttttaaatcaatctaaagtatatatgagtaaacttggtctgacagttaccaatgcttaatcagtgaggcacctatctcagcgatctgtctatttcgttcatccatagttgcctgactccccgtcgtgtagataactacgatacgggagggcttaccatctggccccagtgctgcaatgataccgcgagacccacgctcaccggctccagatttatcagcaataaaccagccagccggaagggccgagcgcagaagtggtcctgcaactttatccgcctccatccagtctattaattgttgccgggaagctagagtaagtagttcgccagttaatagtttgcgcaacgttgttgccattgctacaggcatcgtggtgtcacgctcgtcgtttggtatggcttcattcagctccggttcccaacgatcaaggcgagttacatgatcccccatgttgtgcaaaaaagcggttagctccttcggtcctccgatcgttgtcagaagtaagttggccgcagtgttatcactcatggttatggcagcactgcataattctcttactgtcatgccatccgtaagatgcttttctgtgactggtgagtactcaaccaagtcattctgagaatagtgtatgcggcgaccgagttgctcttgcccggcgtcaatacgggataataccgcgccacatagcagaactttaaaagtgctcatcattggaaaacgttcttcggggcgaaaactctcaaggatcttaccgctgttgagatccagttcgatgtaacccactcgtgcacccaactgatcttcagcatcttttactttcaccagcgtttctgggtgagcaaaaacaggaaggcaaaatgccgcaaaaaagggaataagggcgacacggaaatgttgaatactcatactcttcctttttcaatattattgaagcatttatcagggttattgtctcatgagcggatacatatttgaatgtatttagaaaaataaacaaataggggttccgcgcacatttccccgaaaagtgccacctgacgtctaagaaaccattattatcatgacattaacctataaaaataggcgtatcacgaggccctttcgtctcgcgcgtttcggtgatgacggtgaaaacctctgacacatgcagctcccggagacggtcacagcttgtctgtaagcggatgccgggagcagacaagcccgtcagggcgcgtcagcgggtgttggcgggtgtcggggctggcttaactatgcggcatcagagcagattgtactgagagtgcaccatatgcggtgtgaaataccgcacagatgcgtaaggagaaaataccgcatcaggcgccattcgccattcaggctgcgcaactgttgggaagggcgatcggtgcgggcctcttcgctattacgccagctggcgaaagggggatgtgctgcaaggcgattaagttgggtaacgccagggttttcccagtcacgacgttgtaaaacgacggcgcaaggaatggtgcatgcaaggagatggcgcccaacagtcccccggccacggggcctgccaccatacccacgccgaaacaagcgctcatgagcccgaagtggcgagcccgatcttccccatcggtgatgtcggcgatataggcgccagcaaccgcacctgtggcgccggtgatgccggccacgatgcgtccggcgtagaggcgattagtccaatttgttaaagacag

**V64 (6xNFAT-miniCMV-ZsG-muhCD4)**

Sequence

GATATCCCAACATTACCGCCATGTTGACATTGATTATTGACTAGTTATTAATAGTAATCAATTACGGGGTCATTAGTTCATAGCCCATATATGGAGTTCCGCGTTACATAACTTACGGTAAATGGCCCGCCTGGCTGACCGCCCAACGACCCCCGCCCATTGACGTCAATAATGACGTATGTTCCCATAGTAACGCCAATAGGGACTTTCCATTGACGTCAATGGGTGGAGTATTTACGGTAAACTGCCCACTTGGCAGTACATCAAGTGTATCATATGCCAAGTACGCCCCCTATTGACGTCAATGACGGTAAATGGCCCGCCTGGCATTATGCCCAGTACATGACCTTATGGGACTTTCCTACTTGGCAGTACATCTACGTATTAGTCATCGCTATTACCATGGTGATGCGGTTTTGGCAGTACATCAATGGGCGTGGATAGCGGTTTGACTCACGGGGATTTCCAAGTCTCCACCCCATTGACGTCAATGGGAGTTTGTTTTGGCACCAAAATCAACGGGACTTTCCAAAATGTCGTAACAACTCCGCCCCATTGACGCAAATGGGCGGTAGGCGTGTACGGTGGGAGGTCTATATAAGCAGAGCTCAATAAAAGAGCCCACAACCCCTCACTCGGCGCGCCAGTCTTCCGATAGACTGCGTCGCCCGGGTACCCGTATTCCCAATAAAGCCTCTTGCTGTTTGCATCCGAATCGTGGTCTCGCTGTTCCTTGGGAGGGTCTCCTCTGAGTGATTGACTACCCACGACGGGGGTCTTTCATTTGGGGGCTCGTCCGGGATTTGGAGACCCCTGCCCAGGGACCACCGACCCACCACCGGGAGGTAAGCTGGCCAGCAACTTATCTGTGTCTGTCCGATTGTCTAGTGTCTATGTTTGATGTTATGCGCCTGCGTCTGTACTAGTTAGCTAACTAGCTCTGTATCTGGCGGACCCGTGGTGGAACTGACGAGTTCTGAACACCCGGCCGCAACCCTGGGAGACGTCCCAGGGACTTTGGGGGCCGTTTTTGTGGCCCGACCTGAGGAAGGGAGTCGATGTGGAATCCGACCCCGTCAGGATATGTGGTTCTGGTAGGAGACGAGAACCTAAAACAGTTCCCGCCTCCGTCTGAATTTTTGCTTTCGGTTTGGAACCGAAGCCGCGCGTCTTGTCTGCTGCAGCGCTGCAGCATCGTTCTGTGTTGTCTCTGTCTGACTGTGTTTCTGTATTTGTCTGAAAATTAGGGCCAGACTGTTACCACTCCCTTAAGTTTGACCTTAGGTCACTGGAAAGATGTCGAGCGGATCGCTCACAACCAGTCGGTAGATGTCAAGAAGAGACGTTGGGTTACCTTCTGCTCTGCAGAATGGCCAACCTTTAACGTCGGATGGCCGCGAGACGGCACCTTTAACCGAGACCTCATCACCCAGGTTAAGATCAAGGTCTTTTCACCTGGCCCGCATGGACACCCAGACCAGGTCCCCTACATCGTGACCTGGGAAGCCTTGGCTTTTGACCCCCCTCCCTGGGTCAAGCCCTTTGTACACCCTAAGCCTCCGCCTCCTCTTCCTCCATCCGCCCCGTCTCTCCCCCTTGAACCTCCTCGTTCGACCCCGCCTCGATCCTCCCTTTATCCAGCCCTCACTCCTTCTCTAGGCGCCGGAATTGAAGATCTTCATGTCTGGATCCAAGCTGGAGGAAAAACTGTTTCATACAGAAGGCGTGGAGGAAAAACTGTTTCATACAGAAGGCGTGGAGGAAAAACTGTTTCATACAGAAGGCGTGGAGGAAAAACTGTTTCATACAGAAGGCGTGGAGGAAAAACTGTTTCATACAGAAGGCGTGGAGGAAAAACTGTTTCATACAGAAGGCGTTAGGCGTGTACGGTGGGAGGCCTATATAAGCAGAGCTCGTTTAGTGAACCGTCAGATCGCCTGGAGACGCCATCCACGCTGTTTTGACCTCCATAGAAGACACCGGGACCGATCCAGCCTCCGCGGCCCGGTACCGAGCTCGAATTCACCATGGCCCAGTCCAAGCACGGCCTGACCAAGGAGATGACCATGAAGTACCGCATGGAGGGCTGCGTGGACGGCCACAAGTTCGTGATCACCGGCGAGGGCATCGGCTACCCCTTCAAGGGCAAGCAGGCCATCAACCTGTGCGTGGTGGAGGGCGGCCCCTTGCCCTTCGCCGAGGACATCTTGTCCGCCGCCTTCATGTACGGCAACCGCGTGTTCACCGAGTACCCCCAGGACATCGTCGACTACTTCAAGAACTCCTGCCCCGCCGGCTACACCTGGGACCGCTCCTTCCTGTTCGAGGACGGCGCCGTGTGCATCTGCAACGCCGACATCACCGTGAGCGTGGAGGAGAACTGCATGTACCACGAGTCCAAGTTCTACGGCGTGAACTTCCCCGCCGACGGCCCCGTGATGAAGAAGATGACCGACAACTGGGAGCCCTCCTGCGAGAAGATCATCCCCGTGCCCAAGCAGGGCATCTTGAAGGGCGACGTGAGCATGTACCTGCTGCTGAAGGACGGTGGCCGCTTGCGCTGCCAGTTCGACACCGTGTACAAGGCCAAGTCCGTGCCCCGCAAGATGCCCGACTGGCACTTCATCCAGCACAAGCTGACCCGCGAGGACCGCAGCGACGCCAAGAACCAGAAGTGGCACCTGACCGAGCACGCCATCGCCTCCGGCTCCGCCTTGCCCTGACTCGAGGTTAACGAATTCTACCGGGTAGGGGAGGCGCTTTTCCCAAGGCAGTCTGGAGCATGCGCTTTAGCAGCCCCGCTGGGCACTTGGCGCTACACAAGTGGCCTCTGGCCTCGCACACATTCCACATCCACCGGTAGGCGCCAACCGGCTCCGTTCTTTGGTGGCCCCTTCGCGCCACCTTCTACTCCTCCCCTAGTCAGGAAGTTCCCCCCCGCCCCGCAGCTCGCGTCGTGCAGGACGTGACAAATGGAAGTAGCACGTCTCACTAGTCTCGTGCAGATGGACAGCACCGCTGAGCAATGGAAGCGGGTAGGCCTTTGGGGCAGCGGCCAATAGCAGCTTTGCTCCTTCGCTTTCTGGGCTCAGAGGCTGGGAAGGGGTGGGTCCGGGGGCGGGCTCAGGGGCGGGCTCAGGGGCGGGGCGGGCGCCCGAAGGTCCTCCGGAGGCCCGGCATTCTGCACGCTTCAAAAGCGCACGTCTGCCGCGCTGTTCTCCTCTTCCTCATCTCCGGGCCTTTCGacctgcatcccgccaccATGAACCGGGGAGTCCCTTTTAGGCACTTGCTTCTGGTGCTGCAACTGGCGCTCCTCCCAGCAGCCACTCAGGGAAAGAAAGTGGTGCTGGGCAAAAAAGGGGATACAGTGGAACTGACCTGTACAGCTTCCCAGAAGAAGAGCATACAATTCCACTGGAAAAACTCCAACCAGATAAAGATTCTGGGAAATTACGGCTCCTTCTTATGGAAAGGTCCATCCAAGCTGAATGATCGCGCTGACTCAAGAAGAAGCCTTTGGGACCAAGGAAACTTCCCCCTGATCATCAAGAATCTTAAGATAGAAGACTCAGATACTTACATCTGTGAAGTGGAGGACCAGAAGGAGGAGGTGCAATTGCTAGTGTTCGGATTGACTGCCAACTCTGACACCCACCTGCTTCAGGGGCAGAGCCTGACCCTGACCTTGGAGAGCCCCCCTGGTAGTAGCCCCTCAGTGCAATGTAGGAGTCCAAGGGGTAAAAACATACAGGGGGGGAAGACCCTCTCCGTGTCTCAGCTGGAGCTCCAGGATAGTGGCACCTGGACATGCACTGTCTTGCAGAACCAGAAGAAGGTGGAGTTCAAAATAGACATCGTGGTGCTAGCTTTCCAGAAGGCCTCCAGCATAGTCTATAAGAAAGAGGGGGAACAGGTGGAGTTCTCCTTCCCACTCGCCTTTACAGTTGAAAAGCTGACGGGCAGTGGCGAGCTGTGGTGGCAGGCGGAGAGGGCTTCCTCCTCCAAGTCTTGGATCACCTTTGACCTGAAGAACAAGGAAGTGTCTGTAAAACGGGTTACCCAGGACCCTAAGCTCCAGATGGGCAAGAAGCTCCCGCTCCACCTCACCCTGCCCCAGGCCTTGCCTCAGTATGCTGGCTCTGGAAACCTCACCCTGGCCCTTGAAGCGAAAACAGGAAAGTTGCACCAGGAAGTGAACCTGGTGGTGATGAGAGCCACTCAGCTCCAGAAAAATTTGACCTGTGAGGTGTGGGGACCCACCTCCCCTAAGCTGATGCTGAGCTTGAAACTGGAGAACAAGGAGGCAAAGGTCTCGAAGCGGGAGAAGGCGGTGTGGGTGCTGAACCCTGAGGCGGGGATGTGGCAGTGTCTGCTGAGTGACTCGGGACAGGTCCTGCTGGAATCCAACATCAAGGTTCTGCCCACATGGTCGACCCCGGTGCAGCCAATGGCCCTGATTGTGCTGGGGGGCGTCGCCGGCCTCCTGCTGTTCATTGGGCTAGGCATCTTCTTCTGTGTCAGGTGCCGGCACCGAAGGCGCCAAGCAGAGCGGATGTCTCAGATCAAGAGACTCCTCAGTGAGAAGAAGACCTGCCAGTGCCCTCACCGGTTTCAGAAGACATGTAGCCCCATTTGAAAGCTTCACCAGCTGAAGCCTATAGAGTACGAGCCATAGATAAAATAAAAGATTTTATTTAGTCTCCAGAAAAAGGGGGGAATGAAAGACCCCACCTGTAGGTTTGGCAAGCTAGCTTAAGTAACGCCATTTTGCAAGGCATGGAAAAATACATAACTGAGAATAGAGAAGTTCAGATCAAGGTCAGGAACAGATGGAACAGGGTCGACCCTAGAGAACCATCAGATGTTTCCAGGGTGCCCCAAGGACCTGAAATGACCCTGTGCCTTATTTGAACTAACCAATCAGTTCGCTTCTCGCTTCTGTTCGCGCGCTTCTGCTCCCCGAGCTCAATAAAAGAGCCCACAACCCCTCACTCGGGGCGCCAGTCCTCCGATTGACTGAGTCGCCCGGGTACCCGTGTATCCAATAAACCCTCTTGCAGTTGCATCCGACTTGTGGTCTCGCTGTTCCTTGGGAGGGTCTCCTCTGAGTGATTGACTACCCGTCAGCGGGGGTCTTTCATTTGGGGGCTCGTCCGGGATCGGGAGACCCCTGCCCAGGGACCACCGACCCACCACCGGGAGGTAAGCTGGCTGCCTCGCGCGTTTCGGTGATGACGGTGAAAACCTCTGACATGTGAGCAAAAGGCCAGCAAAAGGCCAGGAACCGTAAAAAGGCCGCGTTGCTGGCGTTTTTCCATAGGCTCCGCCCCCCTGACGAGCATCACAAAAATCGACGCTCAAGTCAGAGGTGGCGAAACCCGACAGGACTATAAAGATACCAGGCGTTTCCCCCTGGAAGCTCCCTCGTGCGCTCTCCTGTTCCGACCCTGCCGCTTACCGGATACCTGTCCGCCTTTCTCCCTTCGGGAAGCGTGGCGCTTTCTCATAGCTCACGCTGTAGGTATCTCAGTTCGGTGTAGGTCGTTCGCTCCAAGCTGGGCTGTGTGCACGAACCCCCCGTTCAGCCCGACCGCTGCGCCTTATCCGGTAACTATCGTCTTGAGTCCAACCCGGTAAGACACGACTTATCGCCACTGGCAGCAGCCACTGGTAACAGGATTAGCAGAGCGAGGTATGTAGGCGGTGCTACAGAGTTCTTGAAGTGGTGGCCTAACTACGGCTACACTAGAAGAACAGTATTTGGTATCTGCGCTCTGCTGAAGCCAGTTACCTTCGGAAAAAGAGTTGGTAGCTCTTGATCCGGCAAACAAACCACCGCTGGTAGCGGTGGTTTTTTTGTTTGCAAGCAGCAGATTACGCGCAGAAAAAAAGGATCTCAAGAAGATCCTTTGATCTTTTCTACGGGGTCTGACGCTCAGTGGAACGAAAACTCACGTTAAGGGATTTTGGTCATGAGATTATCAAAAAGGATCTTCACCTAGATCCTTTTAAATTAAAAATGAAGTTTTAAATCAATCTAAAGTATATATGAGTAAACTTGGTCTGACAGTTACCAATGCTTAATCAGTGAGGCACCTATCTCAGCGATCTGTCTATTTCGTTCATCCATAGTTGCCTGACTCCCCGTCGTGTAGATAACTACGATACGGGAGGGCTTACCATCTGGCCCCAGTGCTGCAATGATACCGCGAGACCCACGCTCACCGGCTCCAGATTTATCAGCAATAAACCAGCCAGCCGGAAGGGCCGAGCGCAGAAGTGGTCCTGCAACTTTATCCGCCTCCATCCAGTCTATTAATTGTTGCCGGGAAGCTAGAGTAAGTAGTTCGCCAGTTAATAGTTTGCGCAACGTTGTTGCCATTGCTACAGGCATCGTGGTGTCACGCTCGTCGTTTGGTATGGCTTCATTCAGCTCCGGTTCCCAACGATCAAGGCGAGTTACATGATCCCCCATGTTGTGCAAAAAAGCGGTTAGCTCCTTCGGTCCTCCGATCGTTGTCAGAAGTAAGTTGGCCGCAGTGTTATCACTCATGGTTATGGCAGCACTGCATAATTCTCTTACTGTCATGCCATCCGTAAGATGCTTTTCTGTGACTGGTGAGTACTCAACCAAGTCATTCTGAGAATAGTGTATGCGGCGACCGAGTTGCTCTTGCCCGGCGTCAATACGGGATAATACCGCGCCACATAGCAGAACTTTAAAAGTGCTCATCATTGGAAAACGTTCTTCGGGGCGAAAACTCTCAAGGATCTTACCGCTGTTGAGATCCAGTTCGATGTAACCCACTCGTGCACCCAACTGATCTTCAGCATCTTTTACTTTCACCAGCGTTTCTGGGTGAGCAAAAACAGGAAGGCAAAATGCCGCAAAAAAGGGAATAAGGGCGACACGGAAATGTTGAATACTCATACTCTTCCTTTTTCAATATTATTGAAGCATTTATCAGGGTTATTGTCTCATGAGCGGATACATATTTGAATGTATTTAGAAAAATAAACAAATAGGGGTTCCGCGCACATTTCCCCGAAAAGTGCCACCTGACGTCTAAGAAACCATTATTATCATGACATTAACCTATAAAAATAGGCGTATCACGAGGCCCTTTCGTCTCGCGCGTTTCGGTGATGACGGTGAAAACCTCTGACACATGCAGCTCCCGGAGACGGTCACAGCTTGTCTGTAAGCGGATGCCGGGAGCAGACAAGCCCGTCAGGGCGCGTCAGCGGGTGTTGGCGGGTGTCGGGGCTGGCTTAACTATGCGGCATCAGAGCAGATTGTACTGAGAGTGCACCATATGCGGTGTGAAATACCGCACAGATGCGTAAGGAGAAAATACCGCATCAGGCGCCATTCGCCATTCAGGCTGCGCAACTGTTGGGAAGGGCGATCGGTGCGGGCCTCTTCGCTATTACGCCAGCTGGCGAAAGGGGGATGTGCTGCAAGGCGATTAAGTTGGGTAACGCCAGGGTTTTCCCAGTCACGACGTTGTAAAACGACGGCGCAAGGAATGGTGCATGCAAGGAGATGGCGCCCAACAGTCCCCCGGCCACGGGGCCTGCCACCATACCCACGCCGAAACAAGCGCTCATGAGCCCGAAGTGGCGAGCCCGATCTTCCCCATCGGTGATGTCGGCGATATAGGCGCCAGCAACCGCACCTGTGGCGCCGGTGATGCCGGCCACGATGCGTCCGGCGTAGAGGCGATTAGTCCAATTTGTTAAAGACAG

**V65 (6xNFAT-TATA-ZsG-muhCD4)**

Sequence

GATATCCCAACATTACCGCCATGTTGACATTGATTATTGACTAGTTATTAATAGTAATCAATTACGGGGTCATTAGTTCATAGCCCATATATGGAGTTCCGCGTTACATAACTTACGGTAAATGGCCCGCCTGGCTGACCGCCCAACGACCCCCGCCCATTGACGTCAATAATGACGTATGTTCCCATAGTAACGCCAATAGGGACTTTCCATTGACGTCAATGGGTGGAGTATTTACGGTAAACTGCCCACTTGGCAGTACATCAAGTGTATCATATGCCAAGTACGCCCCCTATTGACGTCAATGACGGTAAATGGCCCGCCTGGCATTATGCCCAGTACATGACCTTATGGGACTTTCCTACTTGGCAGTACATCTACGTATTAGTCATCGCTATTACCATGGTGATGCGGTTTTGGCAGTACATCAATGGGCGTGGATAGCGGTTTGACTCACGGGGATTTCCAAGTCTCCACCCCATTGACGTCAATGGGAGTTTGTTTTGGCACCAAAATCAACGGGACTTTCCAAAATGTCGTAACAACTCCGCCCCATTGACGCAAATGGGCGGTAGGCGTGTACGGTGGGAGGTCTATATAAGCAGAGCTCAATAAAAGAGCCCACAACCCCTCACTCGGCGCGCCAGTCTTCCGATAGACTGCGTCGCCCGGGTACCCGTATTCCCAATAAAGCCTCTTGCTGTTTGCATCCGAATCGTGGTCTCGCTGTTCCTTGGGAGGGTCTCCTCTGAGTGATTGACTACCCACGACGGGGGTCTTTCATTTGGGGGCTCGTCCGGGATTTGGAGACCCCTGCCCAGGGACCACCGACCCACCACCGGGAGGTAAGCTGGCCAGCAACTTATCTGTGTCTGTCCGATTGTCTAGTGTCTATGTTTGATGTTATGCGCCTGCGTCTGTACTAGTTAGCTAACTAGCTCTGTATCTGGCGGACCCGTGGTGGAACTGACGAGTTCTGAACACCCGGCCGCAACCCTGGGAGACGTCCCAGGGACTTTGGGGGCCGTTTTTGTGGCCCGACCTGAGGAAGGGAGTCGATGTGGAATCCGACCCCGTCAGGATATGTGGTTCTGGTAGGAGACGAGAACCTAAAACAGTTCCCGCCTCCGTCTGAATTTTTGCTTTCGGTTTGGAACCGAAGCCGCGCGTCTTGTCTGCTGCAGCGCTGCAGCATCGTTCTGTGTTGTCTCTGTCTGACTGTGTTTCTGTATTTGTCTGAAAATTAGGGCCAGACTGTTACCACTCCCTTAAGTTTGACCTTAGGTCACTGGAAAGATGTCGAGCGGATCGCTCACAACCAGTCGGTAGATGTCAAGAAGAGACGTTGGGTTACCTTCTGCTCTGCAGAATGGCCAACCTTTAACGTCGGATGGCCGCGAGACGGCACCTTTAACCGAGACCTCATCACCCAGGTTAAGATCAAGGTCTTTTCACCTGGCCCGCATGGACACCCAGACCAGGTCCCCTACATCGTGACCTGGGAAGCCTTGGCTTTTGACCCCCCTCCCTGGGTCAAGCCCTTTGTACACCCTAAGCCTCCGCCTCCTCTTCCTCCATCCGCCCCGTCTCTCCCCCTTGAACCTCCTCGTTCGACCCCGCCTCGATCCTCCCTTTATCCAGCCCTCACTCCTTCTCTAGGCGCCGGAATTGAAGATCTTCATGTCTGGATCCAAGCTGGAGGAAAAACTGTTTCATACAGAAGGCGTGGAGGAAAAACTGTTTCATACAGAAGGCGTGGAGGAAAAACTGTTTCATACAGAAGGCGTGGAGGAAAAACTGTTTCATACAGAAGGCGTGGAGGAAAAACTGTTTCATACAGAAGGCGTGGAGGAAAAACTGTTTCATACAGAAGGCGTCGCGGAGACTCTAGAGGGTATATAATGGATCCCCGGGTACCGAGCTCGAATTCACCATGGCCCAGTCCAAGCACGGCCTGACCAAGGAGATGACCATGAAGTACCGCATGGAGGGCTGCGTGGACGGCCACAAGTTCGTGATCACCGGCGAGGGCATCGGCTACCCCTTCAAGGGCAAGCAGGCCATCAACCTGTGCGTGGTGGAGGGCGGCCCCTTGCCCTTCGCCGAGGACATCTTGTCCGCCGCCTTCATGTACGGCAACCGCGTGTTCACCGAGTACCCCCAGGACATCGTCGACTACTTCAAGAACTCCTGCCCCGCCGGCTACACCTGGGACCGCTCCTTCCTGTTCGAGGACGGCGCCGTGTGCATCTGCAACGCCGACATCACCGTGAGCGTGGAGGAGAACTGCATGTACCACGAGTCCAAGTTCTACGGCGTGAACTTCCCCGCCGACGGCCCCGTGATGAAGAAGATGACCGACAACTGGGAGCCCTCCTGCGAGAAGATCATCCCCGTGCCCAAGCAGGGCATCTTGAAGGGCGACGTGAGCATGTACCTGCTGCTGAAGGACGGTGGCCGCTTGCGCTGCCAGTTCGACACCGTGTACAAGGCCAAGTCCGTGCCCCGCAAGATGCCCGACTGGCACTTCATCCAGCACAAGCTGACCCGCGAGGACCGCAGCGACGCCAAGAACCAGAAGTGGCACCTGACCGAGCACGCCATCGCCTCCGGCTCCGCCTTGCCCTGACTCGAGGTTAACGAATTCTACCGGGTAGGGGAGGCGCTTTTCCCAAGGCAGTCTGGAGCATGCGCTTTAGCAGCCCCGCTGGGCACTTGGCGCTACACAAGTGGCCTCTGGCCTCGCACACATTCCACATCCACCGGTAGGCGCCAACCGGCTCCGTTCTTTGGTGGCCCCTTCGCGCCACCTTCTACTCCTCCCCTAGTCAGGAAGTTCCCCCCCGCCCCGCAGCTCGCGTCGTGCAGGACGTGACAAATGGAAGTAGCACGTCTCACTAGTCTCGTGCAGATGGACAGCACCGCTGAGCAATGGAAGCGGGTAGGCCTTTGGGGCAGCGGCCAATAGCAGCTTTGCTCCTTCGCTTTCTGGGCTCAGAGGCTGGGAAGGGGTGGGTCCGGGGGCGGGCTCAGGGGCGGGCTCAGGGGCGGGGCGGGCGCCCGAAGGTCCTCCGGAGGCCCGGCATTCTGCACGCTTCAAAAGCGCACGTCTGCCGCGCTGTTCTCCTCTTCCTCATCTCCGGGCCTTTCGacctgcatcccgccaccATGAACCGGGGAGTCCCTTTTAGGCACTTGCTTCTGGTGCTGCAACTGGCGCTCCTCCCAGCAGCCACTCAGGGAAAGAAAGTGGTGCTGGGCAAAAAAGGGGATACAGTGGAACTGACCTGTACAGCTTCCCAGAAGAAGAGCATACAATTCCACTGGAAAAACTCCAACCAGATAAAGATTCTGGGAAATTACGGCTCCTTCTTATGGAAAGGTCCATCCAAGCTGAATGATCGCGCTGACTCAAGAAGAAGCCTTTGGGACCAAGGAAACTTCCCCCTGATCATCAAGAATCTTAAGATAGAAGACTCAGATACTTACATCTGTGAAGTGGAGGACCAGAAGGAGGAGGTGCAATTGCTAGTGTTCGGATTGACTGCCAACTCTGACACCCACCTGCTTCAGGGGCAGAGCCTGACCCTGACCTTGGAGAGCCCCCCTGGTAGTAGCCCCTCAGTGCAATGTAGGAGTCCAAGGGGTAAAAACATACAGGGGGGGAAGACCCTCTCCGTGTCTCAGCTGGAGCTCCAGGATAGTGGCACCTGGACATGCACTGTCTTGCAGAACCAGAAGAAGGTGGAGTTCAAAATAGACATCGTGGTGCTAGCTTTCCAGAAGGCCTCCAGCATAGTCTATAAGAAAGAGGGGGAACAGGTGGAGTTCTCCTTCCCACTCGCCTTTACAGTTGAAAAGCTGACGGGCAGTGGCGAGCTGTGGTGGCAGGCGGAGAGGGCTTCCTCCTCCAAGTCTTGGATCACCTTTGACCTGAAGAACAAGGAAGTGTCTGTAAAACGGGTTACCCAGGACCCTAAGCTCCAGATGGGCAAGAAGCTCCCGCTCCACCTCACCCTGCCCCAGGCCTTGCCTCAGTATGCTGGCTCTGGAAACCTCACCCTGGCCCTTGAAGCGAAAACAGGAAAGTTGCACCAGGAAGTGAACCTGGTGGTGATGAGAGCCACTCAGCTCCAGAAAAATTTGACCTGTGAGGTGTGGGGACCCACCTCCCCTAAGCTGATGCTGAGCTTGAAACTGGAGAACAAGGAGGCAAAGGTCTCGAAGCGGGAGAAGGCGGTGTGGGTGCTGAACCCTGAGGCGGGGATGTGGCAGTGTCTGCTGAGTGACTCGGGACAGGTCCTGCTGGAATCCAACATCAAGGTTCTGCCCACATGGTCGACCCCGGTGCAGCCAATGGCCCTGATTGTGCTGGGGGGCGTCGCCGGCCTCCTGCTGTTCATTGGGCTAGGCATCTTCTTCTGTGTCAGGTGCCGGCACCGAAGGCGCCAAGCAGAGCGGATGTCTCAGATCAAGAGACTCCTCAGTGAGAAGAAGACCTGCCAGTGCCCTCACCGGTTTCAGAAGACATGTAGCCCCATTTGAaagcttcaccagctgaagcctatagagtacgagccatagataaaataaaagattttatttagtctccagaaaaaggggggaatgaaagaccccacctgtaggtttggcaagctagcttaagtaacgccattttgcaaggcatggaaaaatacataactgagaatagagaagttcagatcaaggtcaggaacagatggaacagggtcgaccctagagaaccatcagatgtttccagggtgccccaaggacctgaaatgaccctgtgccttatttgaactaaccaatcagttcgcttctcgcttctgttcgcgcgcttctgctccccgagctcaataaaagagcccacaacccctcactcggggcgccagtcctccgattgactgagtcgcccgggtacccgtgtatccaataaaccctcttgcagttgcatccgacttgtggtctcgctgttccttgggagggtctcctctgagtgattgactacccgtcagcgggggtctttcatttgggggctcgtccgggatcgggagacccctgcccagggaccaccgacccaccaccgggaggtaagctggctgcctcgcgcgtttcggtgatgacggtgaaaacctctgacatgtgagcaaaaggccagcaaaaggccaggaaccgtaaaaaggccgcgttgctggcgtttttccataggctccgcccccctgacgagcatcacaaaaatcgacgctcaagtcagaggtggcgaaacccgacaggactataaagataccaggcgtttccccctggaagctccctcgtgcgctctcctgttccgaccctgccgcttaccggatacctgtccgcctttctcccttcgggaagcgtggcgctttctcatagctcacgctgtaggtatctcagttcggtgtaggtcgttcgctccaagctgggctgtgtgcacgaaccccccgttcagcccgaccgctgcgccttatccggtaactatcgtcttgagtccaacccggtaagacacgacttatcgccactggcagcagccactggtaacaggattagcagagcgaggtatgtaggcggtgctacagagttcttgaagtggtggcctaactacggctacactagaagaacagtatttggtatctgcgctctgctgaagccagttaccttcggaaaaagagttggtagctcttgatccggcaaacaaaccaccgctggtagcggtggtttttttgtttgcaagcagcagattacgcgcagaaaaaaaggatctcaagaagatcctttgatcttttctacggggtctgacgctcagtggaacgaaaactcacgttaagggattttggtcatgagattatcaaaaaggatcttcacctagatccttttaaattaaaaatgaagttttaaatcaatctaaagtatatatgagtaaacttggtctgacagttaccaatgcttaatcagtgaggcacctatctcagcgatctgtctatttcgttcatccatagttgcctgactccccgtcgtgtagataactacgatacgggagggcttaccatctggccccagtgctgcaatgataccgcgagacccacgctcaccggctccagatttatcagcaataaaccagccagccggaagggccgagcgcagaagtggtcctgcaactttatccgcctccatccagtctattaattgttgccgggaagctagagtaagtagttcgccagttaatagtttgcgcaacgttgttgccattgctacaggcatcgtggtgtcacgctcgtcgtttggtatggcttcattcagctccggttcccaacgatcaaggcgagttacatgatcccccatgttgtgcaaaaaagcggttagctccttcggtcctccgatcgttgtcagaagtaagttggccgcagtgttatcactcatggttatggcagcactgcataattctcttactgtcatgccatccgtaagatgcttttctgtgactggtgagtactcaaccaagtcattctgagaatagtgtatgcggcgaccgagttgctcttgcccggcgtcaatacgggataataccgcgccacatagcagaactttaaaagtgctcatcattggaaaacgttcttcggggcgaaaactctcaaggatcttaccgctgttgagatccagttcgatgtaacccactcgtgcacccaactgatcttcagcatcttttactttcaccagcgtttctgggtgagcaaaaacaggaaggcaaaatgccgcaaaaaagggaataagggcgacacggaaatgttgaatactcatactcttcctttttcaatattattgaagcatttatcagggttattgtctcatgagcggatacatatttgaatgtatttagaaaaataaacaaataggggttccgcgcacatttccccgaaaagtgccacctgacgtctaagaaaccattattatcatgacattaacctataaaaataggcgtatcacgaggccctttcgtctcgcgcgtttcggtgatgacggtgaaaacctctgacacatgcagctcccggagacggtcacagcttgtctgtaagcggatgccgggagcagacaagcccgtcagggcgcgtcagcgggtgttggcgggtgtcggggctggcttaactatgcggcatcagagcagattgtactgagagtgcaccatatgcggtgtgaaataccgcacagatgcgtaaggagaaaataccgcatcaggcgccattcgccattcaggctgcgcaactgttgggaagggcgatcggtgcgggcctcttcgctattacgccagctggcgaaagggggatgtgctgcaaggcgattaagttgggtaacgccagggttttcccagtcacgacgttgtaaaacgacggcgcaaggaatggtgcatgcaaggagatggcgcccaacagtcccccggccacggggcctgccaccatacccacgccgaaacaagcgctcatgagcccgaagtggcgagcccgatcttccccatcggtgatgtcggcgatataggcgccagcaaccgcacctgtggcgccggtgatgccggccacgatgcgtccggcgtagaggcgattagtccaatttgttaaagacag

**V152 (4xNFAT-TATA-ZsG-muhCD4)**

Sequence

GATATCCCAACATTACCGCCATGTTGACATTGATTATTGACTAGTTATTAATAGTAATCAATTACGGGGTCATTAGTTCATAGCCCATATATGGAGTTCCGCGTTACATAACTTACGGTAAATGGCCCGCCTGGCTGACCGCCCAACGACCCCCGCCCATTGACGTCAATAATGACGTATGTTCCCATAGTAACGCCAATAGGGACTTTCCATTGACGTCAATGGGTGGAGTATTTACGGTAAACTGCCCACTTGGCAGTACATCAAGTGTATCATATGCCAAGTACGCCCCCTATTGACGTCAATGACGGTAAATGGCCCGCCTGGCATTATGCCCAGTACATGACCTTATGGGACTTTCCTACTTGGCAGTACATCTACGTATTAGTCATCGCTATTACCATGGTGATGCGGTTTTGGCAGTACATCAATGGGCGTGGATAGCGGTTTGACTCACGGGGATTTCCAAGTCTCCACCCCATTGACGTCAATGGGAGTTTGTTTTGGCACCAAAATCAACGGGACTTTCCAAAATGTCGTAACAACTCCGCCCCATTGACGCAAATGGGCGGTAGGCGTGTACGGTGGGAGGTCTATATAAGCAGAGCTCAATAAAAGAGCCCACAACCCCTCACTCGGCGCGCCAGTCTTCCGATAGACTGCGTCGCCCGGGTACCCGTATTCCCAATAAAGCCTCTTGCTGTTTGCATCCGAATCGTGGTCTCGCTGTTCCTTGGGAGGGTCTCCTCTGAGTGATTGACTACCCACGACGGGGGTCTTTCATTTGGGGGCTCGTCCGGGATTTGGAGACCCCTGCCCAGGGACCACCGACCCACCACCGGGAGGTAAGCTGGCCAGCAACTTATCTGTGTCTGTCCGATTGTCTAGTGTCTATGTTTGATGTTATGCGCCTGCGTCTGTACTAGTTAGCTAACTAGCTCTGTATCTGGCGGACCCGTGGTGGAACTGACGAGTTCTGAACACCCGGCCGCAACCCTGGGAGACGTCCCAGGGACTTTGGGGGCCGTTTTTGTGGCCCGACCTGAGGAAGGGAGTCGATGTGGAATCCGACCCCGTCAGGATATGTGGTTCTGGTAGGAGACGAGAACCTAAAACAGTTCCCGCCTCCGTCTGAATTTTTGCTTTCGGTTTGGAACCGAAGCCGCGCGTCTTGTCTGCTGCAGCGCTGCAGCATCGTTCTGTGTTGTCTCTGTCTGACTGTGTTTCTGTATTTGTCTGAAAATTAGGGCCAGACTGTTACCACTCCCTTAAGTTTGACCTTAGGTCACTGGAAAGATGTCGAGCGGATCGCTCACAACCAGTCGGTAGATGTCAAGAAGAGACGTTGGGTTACCTTCTGCTCTGCAGAATGGCCAACCTTTAACGTCGGATGGCCGCGAGACGGCACCTTTAACCGAGACCTCATCACCCAGGTTAAGATCAAGGTCTTTTCACCTGGCCCGCATGGACACCCAGACCAGGTCCCCTACATCGTGACCTGGGAAGCCTTGGCTTTTGACCCCCCTCCCTGGGTCAAGCCCTTTGTACACCCTAAGCCTCCGCCTCCTCTTCCTCCATCCGCCCCGTCTCTCCCCCTTGAACCTCCTCGTTCGACCCCGCCTCGATCCTCCCTTTATCCAGCCCTCACTCCTTCTCTAGGCGCCGGAATTGAAGATCTTCATGTCTGGATCCAAGCTGGAGGAAAAACTGTTTCATACAGAAGGCGTGGAGGAAAAACTGTTTCATACAGAAGGCGTGGAGGAAAAACTGTTTCATACAGAAGGCGTGGAGGAAAAACTGTTTCATACAGAAGGCGTCGCGGAGACTCTAGAGGGTATATAATGGAAGCTCGAATTCCAGTCCCCGAGATCTCGCCACCATGGCCCAGTCCAAGCACGGCCTGACCAAGGAGATGACCATGAAGTACCGCATGGAGGGCTGCGTGGACGGCCACAAGTTCGTGATCACCGGCGAGGGCATCGGCTACCCCTTCAAGGGCAAGCAGGCCATCAACCTGTGCGTGGTGGAGGGCGGCCCCTTGCCCTTCGCCGAGGACATCTTGTCCGCCGCCTTCATGTACGGCAACCGCGTGTTCACCGAGTACCCCCAGGACATCGTCGACTACTTCAAGAACTCCTGCCCCGCCGGCTACACCTGGGACCGCTCCTTCCTGTTCGAGGACGGCGCCGTGTGCATCTGCAACGCCGACATCACCGTGAGCGTGGAGGAGAACTGCATGTACCACGAGTCCAAGTTCTACGGCGTGAACTTCCCCGCCGACGGCCCCGTGATGAAGAAGATGACCGACAACTGGGAGCCCTCCTGCGAGAAGATCATCCCCGTGCCCAAGCAGGGCATCTTGAAGGGCGACGTGAGCATGTACCTGCTGCTGAAGGACGGTGGCCGCTTGCGCTGCCAGTTCGACACCGTGTACAAGGCCAAGTCCGTGCCCCGCAAGATGCCCGACTGGCACTTCATCCAGCACAAGCTGACCCGCGAGGACCGCAGCGACGCCAAGAACCAGAAGTGGCACCTGACCGAGCACGCCATCGCCTCCGGCTCCGCCTTGCCCTGACTCGAGGTTAACGAATTCTACCGGGTAGGGGAGGCGCTTTTCCCAAGGCAGTCTGGAGCATGCGCTTTAGCAGCCCCGCTGGGCACTTGGCGCTACACAAGTGGCCTCTGGCCTCGCACACATTCCACATCCACCGGTAGGCGCCAACCGGCTCCGTTCTTTGGTGGCCCCTTCGCGCCACCTTCTACTCCTCCCCTAGTCAGGAAGTTCCCCCCCGCCCCGCAGCTCGCGTCGTGCAGGACGTGACAAATGGAAGTAGCACGTCTCACTAGTCTCGTGCAGATGGACAGCACCGCTGAGCAATGGAAGCGGGTAGGCCTTTGGGGCAGCGGCCAATAGCAGCTTTGCTCCTTCGCTTTCTGGGCTCAGAGGCTGGGAAGGGGTGGGTCCGGGGGCGGGCTCAGGGGCGGGCTCAGGGGCGGGGCGGGCGCCCGAAGGTCCTCCGGAGGCCCGGCATTCTGCACGCTTCAAAAGCGCACGTCTGCCGCGCTGTTCTCCTCTTCCTCATCTCCGGGCCTTTCGacctgcatcccgccaccATGAACCGGGGAGTCCCTTTTAGGCACTTGCTTCTGGTGCTGCAACTGGCGCTCCTCCCAGCAGCCACTCAGGGAAAGAAAGTGGTGCTGGGCAAAAAAGGGGATACAGTGGAACTGACCTGTACAGCTTCCCAGAAGAAGAGCATACAATTCCACTGGAAAAACTCCAACCAGATAAAGATTCTGGGAAATTACGGCTCCTTCTTATGGAAAGGTCCATCCAAGCTGAATGATCGCGCTGACTCAAGAAGAAGCCTTTGGGACCAAGGAAACTTCCCCCTGATCATCAAGAATCTTAAGATAGAAGACTCAGATACTTACATCTGTGAAGTGGAGGACCAGAAGGAGGAGGTGCAATTGCTAGTGTTCGGATTGACTGCCAACTCTGACACCCACCTGCTTCAGGGGCAGAGCCTGACCCTGACCTTGGAGAGCCCCCCTGGTAGTAGCCCCTCAGTGCAATGTAGGAGTCCAAGGGGTAAAAACATACAGGGGGGGAAGACCCTCTCCGTGTCTCAGCTGGAGCTCCAGGATAGTGGCACCTGGACATGCACTGTCTTGCAGAACCAGAAGAAGGTGGAGTTCAAAATAGACATCGTGGTGCTAGCTTTCCAGAAGGCCTCCAGCATAGTCTATAAGAAAGAGGGGGAACAGGTGGAGTTCTCCTTCCCACTCGCCTTTACAGTTGAAAAGCTGACGGGCAGTGGCGAGCTGTGGTGGCAGGCGGAGAGGGCTTCCTCCTCCAAGTCTTGGATCACCTTTGACCTGAAGAACAAGGAAGTGTCTGTAAAACGGGTTACCCAGGACCCTAAGCTCCAGATGGGCAAGAAGCTCCCGCTCCACCTCACCCTGCCCCAGGCCTTGCCTCAGTATGCTGGCTCTGGAAACCTCACCCTGGCCCTTGAAGCGAAAACAGGAAAGTTGCACCAGGAAGTGAACCTGGTGGTGATGAGAGCCACTCAGCTCCAGAAAAATTTGACCTGTGAGGTGTGGGGACCCACCTCCCCTAAGCTGATGCTGAGCTTGAAACTGGAGAACAAGGAGGCAAAGGTCTCGAAGCGGGAGAAGGCGGTGTGGGTGCTGAACCCTGAGGCGGGGATGTGGCAGTGTCTGCTGAGTGACTCGGGACAGGTCCTGCTGGAATCCAACATCAAGGTTCTGCCCACATGGTCGACCCCGGTGCAGCCAATGGCCCTGATTGTGCTGGGGGGCGTCGCCGGCCTCCTGCTGTTCATTGGGCTAGGCATCTTCTTCTGTGTCAGGTGCCGGCACCGAAGGCGCCAAGCAGAGCGGATGTCTCAGATCAAGAGACTCCTCAGTGAGAAGAAGACCTGCCAGTGCCCTCACCGGTTTCAGAAGACATGTAGCCCCATTTGAaagcttcaccagctgaagcctatagagtacgagccatagataaaataaaagattttatttagtctccagaaaaaggggggaatgaaagaccccacctgtaggtttggcaagctagcttaagtaacgccattttgcaaggcatggaaaaatacataactgagaatagagaagttcagatcaaggtcaggaacagatggaacagggtcgaccctagagaaccatcagatgtttccagggtgccccaaggacctgaaatgaccctgtgccttatttgaactaaccaatcagttcgcttctcgcttctgttcgcgcgcttctgctccccgagctcaataaaagagcccacaacccctcactcggggcgccagtcctccgattgactgagtcgcccgggtacccgtgtatccaataaaccctcttgcagttgcatccgacttgtggtctcgctgttccttgggagggtctcctctgagtgattgactacccgtcagcgggggtctttcatttgggggctcgtccgggatcgggagacccctgcccagggaccaccgacccaccaccgggaggtaagctggctgcctcgcgcgtttcggtgatgacggtgaaaacctctgacatgtgagcaaaaggccagcaaaaggccaggaaccgtaaaaaggccgcgttgctggcgtttttccataggctccgcccccctgacgagcatcacaaaaatcgacgctcaagtcagaggtggcgaaacccgacaggactataaagataccaggcgtttccccctggaagctccctcgtgcgctctcctgttccgaccctgccgcttaccggatacctgtccgcctttctcccttcgggaagcgtggcgctttctcatagctcacgctgtaggtatctcagttcggtgtaggtcgttcgctccaagctgggctgtgtgcacgaaccccccgttcagcccgaccgctgcgccttatccggtaactatcgtcttgagtccaacccggtaagacacgacttatcgccactggcagcagccactggtaacaggattagcagagcgaggtatgtaggcggtgctacagagttcttgaagtggtggcctaactacggctacactagaagaacagtatttggtatctgcgctctgctgaagccagttaccttcggaaaaagagttggtagctcttgatccggcaaacaaaccaccgctggtagcggtggtttttttgtttgcaagcagcagattacgcgcagaaaaaaaggatctcaagaagatcctttgatcttttctacggggtctgacgctcagtggaacgaaaactcacgttaagggattttggtcatgagattatcaaaaaggatcttcacctagatccttttaaattaaaaatgaagttttaaatcaatctaaagtatatatgagtaaacttggtctgacagttaccaatgcttaatcagtgaggcacctatctcagcgatctgtctatttcgttcatccatagttgcctgactccccgtcgtgtagataactacgatacgggagggcttaccatctggccccagtgctgcaatgataccgcgagacccacgctcaccggctccagatttatcagcaataaaccagccagccggaagggccgagcgcagaagtggtcctgcaactttatccgcctccatccagtctattaattgttgccgggaagctagagtaagtagttcgccagttaatagtttgcgcaacgttgttgccattgctacaggcatcgtggtgtcacgctcgtcgtttggtatggcttcattcagctccggttcccaacgatcaaggcgagttacatgatcccccatgttgtgcaaaaaagcggttagctccttcggtcctccgatcgttgtcagaagtaagttggccgcagtgttatcactcatggttatggcagcactgcataattctcttactgtcatgccatccgtaagatgcttttctgtgactggtgagtactcaaccaagtcattctgagaatagtgtatgcggcgaccgagttgctcttgcccggcgtcaatacgggataataccgcgccacatagcagaactttaaaagtgctcatcattggaaaacgttcttcggggcgaaaactctcaaggatcttaccgctgttgagatccagttcgatgtaacccactcgtgcacccaactgatcttcagcatcttttactttcaccagcgtttctgggtgagcaaaaacaggaaggcaaaatgccgcaaaaaagggaataagggcgacacggaaatgttgaatactcatactcttcctttttcaatattattgaagcatttatcagggttattgtctcatgagcggatacatatttgaatgtatttagaaaaataaacaaataggggttccgcgcacatttccccgaaaagtgccacctgacgtctaagaaaccattattatcatgacattaacctataaaaataggcgtatcacgaggccctttcgtctcgcgcgtttcggtgatgacggtgaaaacctctgacacatgcagctcccggagacggtcacagcttgtctgtaagcggatgccgggagcagacaagcccgtcagggcgcgtcagcgggtgttggcgggtgtcggggctggcttaactatgcggcatcagagcagattgtactgagagtgcaccatatgcggtgtgaaataccgcacagatgcgtaaggagaaaataccgcatcaggcgccattcgccattcaggctgcgcaactgttgggaagggcgatcggtgcgggcctcttcgctattacgccagctggcgaaagggggatgtgctgcaaggcgattaagttgggtaacgccagggttttcccagtcacgacgttgtaaaacgacggcgcaaggaatggtgcatgcaaggagatggcgcccaacagtcccccggccacggggcctgccaccatacccacgccgaaacaagcgctcatgagcccgaagtggcgagcccgatcttccccatcggtgatgtcggcgatataggcgccagcaaccgcacctgtggcgccggtgatgccggccacgatgcgtccggcgtagaggcgattagtccaatttgttaaagacag

**V74 (8xNFAT-TATA-ZsG-muhCD4)**

Sequence

GATATCCCAACATTACCGCCATGTTGACATTGATTATTGACTAGTTATTAATAGTAATCAATTACGGGGTCATTAGTTCATAGCCCATATATGGAGTTCCGCGTTACATAACTTACGGTAAATGGCCCGCCTGGCTGACCGCCCAACGACCCCCGCCCATTGACGTCAATAATGACGTATGTTCCCATAGTAACGCCAATAGGGACTTTCCATTGACGTCAATGGGTGGAGTATTTACGGTAAACTGCCCACTTGGCAGTACATCAAGTGTATCATATGCCAAGTACGCCCCCTATTGACGTCAATGACGGTAAATGGCCCGCCTGGCATTATGCCCAGTACATGACCTTATGGGACTTTCCTACTTGGCAGTACATCTACGTATTAGTCATCGCTATTACCATGGTGATGCGGTTTTGGCAGTACATCAATGGGCGTGGATAGCGGTTTGACTCACGGGGATTTCCAAGTCTCCACCCCATTGACGTCAATGGGAGTTTGTTTTGGCACCAAAATCAACGGGACTTTCCAAAATGTCGTAACAACTCCGCCCCATTGACGCAAATGGGCGGTAGGCGTGTACGGTGGGAGGTCTATATAAGCAGAGCTCAATAAAAGAGCCCACAACCCCTCACTCGGCGCGCCAGTCTTCCGATAGACTGCGTCGCCCGGGTACCCGTATTCCCAATAAAGCCTCTTGCTGTTTGCATCCGAATCGTGGTCTCGCTGTTCCTTGGGAGGGTCTCCTCTGAGTGATTGACTACCCACGACGGGGGTCTTTCATTTGGGGGCTCGTCCGGGATTTGGAGACCCCTGCCCAGGGACCACCGACCCACCACCGGGAGGTAAGCTGGCCAGCAACTTATCTGTGTCTGTCCGATTGTCTAGTGTCTATGTTTGATGTTATGCGCCTGCGTCTGTACTAGTTAGCTAACTAGCTCTGTATCTGGCGGACCCGTGGTGGAACTGACGAGTTCTGAACACCCGGCCGCAACCCTGGGAGACGTCCCAGGGACTTTGGGGGCCGTTTTTGTGGCCCGACCTGAGGAAGGGAGTCGATGTGGAATCCGACCCCGTCAGGATATGTGGTTCTGGTAGGAGACGAGAACCTAAAACAGTTCCCGCCTCCGTCTGAATTTTTGCTTTCGGTTTGGAACCGAAGCCGCGCGTCTTGTCTGCTGCAGCGCTGCAGCATCGTTCTGTGTTGTCTCTGTCTGACTGTGTTTCTGTATTTGTCTGAAAATTAGGGCCAGACTGTTACCACTCCCTTAAGTTTGACCTTAGGTCACTGGAAAGATGTCGAGCGGATCGCTCACAACCAGTCGGTAGATGTCAAGAAGAGACGTTGGGTTACCTTCTGCTCTGCAGAATGGCCAACCTTTAACGTCGGATGGCCGCGAGACGGCACCTTTAACCGAGACCTCATCACCCAGGTTAAGATCAAGGTCTTTTCACCTGGCCCGCATGGACACCCAGACCAGGTCCCCTACATCGTGACCTGGGAAGCCTTGGCTTTTGACCCCCCTCCCTGGGTCAAGCCCTTTGTACACCCTAAGCCTCCGCCTCCTCTTCCTCCATCCGCCCCGTCTCTCCCCCTTGAACCTCCTCGTTCGACCCCGCCTCGATCCTCCCTTTATCCAGCCCTCACTCCTTCTCTAGGCGCCGGAATTGAAGATCATCATGTCTGGATCCAAGCTGGAGGAAAAACTGTTTCATACAGAAGGCGTGGAGGAAAAACTGTTTCATACAGAAGGCGTGGAGGAAAAACTGTTTCATACAGAAGGCGTGGAGGAAAAACTGTTTCATACAGAAGGCGTGGAGGAAAAACTGTTTCATACAGAAGGCGTGGAGGAAAAACTGTTTCATACAGAAGGCGTGGAGGAAAAACTGTTTCATACAGAAGGCGTGGAGGAAAAACTGTTTCATACAGAAGGCGTCGCGGAGACTCTAGAGGGTATATAATGGAAGCTCGAATTCCAGTCCCCGAGATCTCGCCACCATGGCCCAGTCCAAGCACGGCCTGACCAAGGAGATGACCATGAAGTACCGCATGGAGGGCTGCGTGGACGGCCACAAGTTCGTGATCACCGGCGAGGGCATCGGCTACCCCTTCAAGGGCAAGCAGGCCATCAACCTGTGCGTGGTGGAGGGCGGCCCCTTGCCCTTCGCCGAGGACATCTTGTCCGCCGCCTTCATGTACGGCAACCGCGTGTTCACCGAGTACCCCCAGGACATCGTCGACTACTTCAAGAACTCCTGCCCCGCCGGCTACACCTGGGACCGCTCCTTCCTGTTCGAGGACGGCGCCGTGTGCATCTGCAACGCCGACATCACCGTGAGCGTGGAGGAGAACTGCATGTACCACGAGTCCAAGTTCTACGGCGTGAACTTCCCCGCCGACGGCCCCGTGATGAAGAAGATGACCGACAACTGGGAGCCCTCCTGCGAGAAGATCATCCCCGTGCCCAAGCAGGGCATCTTGAAGGGCGACGTGAGCATGTACCTGCTGCTGAAGGACGGTGGCCGCTTGCGCTGCCAGTTCGACACCGTGTACAAGGCCAAGTCCGTGCCCCGCAAGATGCCCGACTGGCACTTCATCCAGCACAAGCTGACCCGCGAGGACCGCAGCGACGCCAAGAACCAGAAGTGGCACCTGACCGAGCACGCCATCGCCTCCGGCTCCGCCTTGCCCTGACTCGAGGTTAACGAATTCTACCGGGTAGGGGAGGCGCTTTTCCCAAGGCAGTCTGGAGCATGCGCTTTAGCAGCCCCGCTGGGCACTTGGCGCTACACAAGTGGCCTCTGGCCTCGCACACATTCCACATCCACCGGTAGGCGCCAACCGGCTCCGTTCTTTGGTGGCCCCTTCGCGCCACCTTCTACTCCTCCCCTAGTCAGGAAGTTCCCCCCCGCCCCGCAGCTCGCGTCGTGCAGGACGTGACAAATGGAAGTAGCACGTCTCACTAGTCTCGTGCAGATGGACAGCACCGCTGAGCAATGGAAGCGGGTAGGCCTTTGGGGCAGCGGCCAATAGCAGCTTTGCTCCTTCGCTTTCTGGGCTCAGAGGCTGGGAAGGGGTGGGTCCGGGGGCGGGCTCAGGGGCGGGCTCAGGGGCGGGGCGGGCGCCCGAAGGTCCTCCGGAGGCCCGGCATTCTGCACGCTTCAAAAGCGCACGTCTGCCGCGCTGTTCTCCTCTTCCTCATCTCCGGGCCTTTCGacctgcatcccgccaccATGAACCGGGGAGTCCCTTTTAGGCACTTGCTTCTGGTGCTGCAACTGGCGCTCCTCCCAGCAGCCACTCAGGGAAAGAAAGTGGTGCTGGGCAAAAAAGGGGATACAGTGGAACTGACCTGTACAGCTTCCCAGAAGAAGAGCATACAATTCCACTGGAAAAACTCCAACCAGATAAAGATTCTGGGAAATTACGGCTCCTTCTTATGGAAAGGTCCATCCAAGCTGAATGATCGCGCTGACTCAAGAAGAAGCCTTTGGGACCAAGGAAACTTCCCCCTGATCATCAAGAATCTTAAGATAGAAGACTCAGATACTTACATCTGTGAAGTGGAGGACCAGAAGGAGGAGGTGCAATTGCTAGTGTTCGGATTGACTGCCAACTCTGACACCCACCTGCTTCAGGGGCAGAGCCTGACCCTGACCTTGGAGAGCCCCCCTGGTAGTAGCCCCTCAGTGCAATGTAGGAGTCCAAGGGGTAAAAACATACAGGGGGGGAAGACCCTCTCCGTGTCTCAGCTGGAGCTCCAGGATAGTGGCACCTGGACATGCACTGTCTTGCAGAACCAGAAGAAGGTGGAGTTCAAAATAGACATCGTGGTGCTAGCTTTCCAGAAGGCCTCCAGCATAGTCTATAAGAAAGAGGGGGAACAGGTGGAGTTCTCCTTCCCACTCGCCTTTACAGTTGAAAAGCTGACGGGCAGTGGCGAGCTGTGGTGGCAGGCGGAGAGGGCTTCCTCCTCCAAGTCTTGGATCACCTTTGACCTGAAGAACAAGGAAGTGTCTGTAAAACGGGTTACCCAGGACCCTAAGCTCCAGATGGGCAAGAAGCTCCCGCTCCACCTCACCCTGCCCCAGGCCTTGCCTCAGTATGCTGGCTCTGGAAACCTCACCCTGGCCCTTGAAGCGAAAACAGGAAAGTTGCACCAGGAAGTGAACCTGGTGGTGATGAGAGCCACTCAGCTCCAGAAAAATTTGACCTGTGAGGTGTGGGGACCCACCTCCCCTAAGCTGATGCTGAGCTTGAAACTGGAGAACAAGGAGGCAAAGGTCTCGAAGCGGGAGAAGGCGGTGTGGGTGCTGAACCCTGAGGCGGGGATGTGGCAGTGTCTGCTGAGTGACTCGGGACAGGTCCTGCTGGAATCCAACATCAAGGTTCTGCCCACATGGTCGACCCCGGTGCAGCCAATGGCCCTGATTGTGCTGGGGGGCGTCGCCGGCCTCCTGCTGTTCATTGGGCTAGGCATCTTCTTCTGTGTCAGGTGCCGGCACCGAAGGCGCCAAGCAGAGCGGATGTCTCAGATCAAGAGACTCCTCAGTGAGAAGAAGACCTGCCAGTGCCCTCACCGGTTTCAGAAGACATGTAGCCCCATTTGAaagcttcaccagctgaagcctatagagtacgagccatagataaaataaaagattttatttagtctccagaaaaaggggggaatgaaagaccccacctgtaggtttggcaagctagcttaagtaacgccattttgcaaggcatggaaaaatacataactgagaatagagaagttcagatcaaggtcaggaacagatggaacagggtcgaccctagagaaccatcagatgtttccagggtgccccaaggacctgaaatgaccctgtgccttatttgaactaaccaatcagttcgcttctcgcttctgttcgcgcgcttctgctccccgagctcaataaaagagcccacaacccctcactcggggcgccagtcctccgattgactgagtcgcccgggtacccgtgtatccaataaaccctcttgcagttgcatccgacttgtggtctcgctgttccttgggagggtctcctctgagtgattgactacccgtcagcgggggtctttcatttgggggctcgtccgggatcgggagacccctgcccagggaccaccgacccaccaccgggaggtaagctggctgcctcgcgcgtttcggtgatgacggtgaaaacctctgacatgtgagcaaaaggccagcaaaaggccaggaaccgtaaaaaggccgcgttgctggcgtttttccataggctccgcccccctgacgagcatcacaaaaatcgacgctcaagtcagaggtggcgaaacccgacaggactataaagataccaggcgtttccccctggaagctccctcgtgcgctctcctgttccgaccctgccgcttaccggatacctgtccgcctttctcccttcgggaagcgtggcgctttctcatagctcacgctgtaggtatctcagttcggtgtaggtcgttcgctccaagctgggctgtgtgcacgaaccccccgttcagcccgaccgctgcgccttatccggtaactatcgtcttgagtccaacccggtaagacacgacttatcgccactggcagcagccactggtaacaggattagcagagcgaggtatgtaggcggtgctacagagttcttgaagtggtggcctaactacggctacactagaagaacagtatttggtatctgcgctctgctgaagccagttaccttcggaaaaagagttggtagctcttgatccggcaaacaaaccaccgctggtagcggtggtttttttgtttgcaagcagcagattacgcgcagaaaaaaaggatctcaagaagatcctttgatcttttctacggggtctgacgctcagtggaacgaaaactcacgttaagggattttggtcatgagattatcaaaaaggatcttcacctagatccttttaaattaaaaatgaagttttaaatcaatctaaagtatatatgagtaaacttggtctgacagttaccaatgcttaatcagtgaggcacctatctcagcgatctgtctatttcgttcatccatagttgcctgactccccgtcgtgtagataactacgatacgggagggcttaccatctggccccagtgctgcaatgataccgcgagacccacgctcaccggctccagatttatcagcaataaaccagccagccggaagggccgagcgcagaagtggtcctgcaactttatccgcctccatccagtctattaattgttgccgggaagctagagtaagtagttcgccagttaatagtttgcgcaacgttgttgccattgctacaggcatcgtggtgtcacgctcgtcgtttggtatggcttcattcagctccggttcccaacgatcaaggcgagttacatgatcccccatgttgtgcaaaaaagcggttagctccttcggtcctccgatcgttgtcagaagtaagttggccgcagtgttatcactcatggttatggcagcactgcataattctcttactgtcatgccatccgtaagatgcttttctgtgactggtgagtactcaaccaagtcattctgagaatagtgtatgcggcgaccgagttgctcttgcccggcgtcaatacgggataataccgcgccacatagcagaactttaaaagtgctcatcattggaaaacgttcttcggggcgaaaactctcaaggatcttaccgctgttgagatccagttcgatgtaacccactcgtgcacccaactgatcttcagcatcttttactttcaccagcgtttctgggtgagcaaaaacaggaaggcaaaatgccgcaaaaaagggaataagggcgacacggaaatgttgaatactcatactcttcctttttcaatattattgaagcatttatcagggttattgtctcatgagcggatacatatttgaatgtatttagaaaaataaacaaataggggttccgcgcacatttccccgaaaagtgccacctgacgtctaagaaaccattattatcatgacattaacctataaaaataggcgtatcacgaggccctttcgtctcgcgcgtttcggtgatgacggtgaaaacctctgacacatgcagctcccggagacggtcacagcttgtctgtaagcggatgccgggagcagacaagcccgtcagggcgcgtcagcgggtgttggcgggtgtcggggctggcttaactatgcggcatcagagcagattgtactgagagtgcaccatatgcggtgtgaaataccgcacagatgcgtaaggagaaaataccgcatcaggcgccattcgccattcaggctgcgcaactgttgggaagggcgatcggtgcgggcctcttcgctattacgccagctggcgaaagggggatgtgctgcaaggcgattaagttgggtaacgccagggttttcccagtcacgacgttgtaaaacgacggcgcaaggaatggtgcatgcaaggagatggcgcccaacagtcccccggccacggggcctgccaccatacccacgccgaaacaagcgctcatgagcccgaagtggcgagcccgatcttccccatcggtgatgtcggcgatataggcgccagcaaccgcacctgtggcgccggtgatgccggccacgatgcgtccggcgtagaggcgattagtccaatttgttaaagacag

**V75 (8xNFAT-TATA-ZsG-hCD8)**

Sequence

GATATCCCAACATTACCGCCATGTTGACATTGATTATTGACTAGTTATTAATAGTAATCAATTACGGGGTCATTAGTTCATAGCCCATATATGGAGTTCCGCGTTACATAACTTACGGTAAATGGCCCGCCTGGCTGACCGCCCAACGACCCCCGCCCATTGACGTCAATAATGACGTATGTTCCCATAGTAACGCCAATAGGGACTTTCCATTGACGTCAATGGGTGGAGTATTTACGGTAAACTGCCCACTTGGCAGTACATCAAGTGTATCATATGCCAAGTACGCCCCCTATTGACGTCAATGACGGTAAATGGCCCGCCTGGCATTATGCCCAGTACATGACCTTATGGGACTTTCCTACTTGGCAGTACATCTACGTATTAGTCATCGCTATTACCATGGTGATGCGGTTTTGGCAGTACATCAATGGGCGTGGATAGCGGTTTGACTCACGGGGATTTCCAAGTCTCCACCCCATTGACGTCAATGGGAGTTTGTTTTGGCACCAAAATCAACGGGACTTTCCAAAATGTCGTAACAACTCCGCCCCATTGACGCAAATGGGCGGTAGGCGTGTACGGTGGGAGGTCTATATAAGCAGAGCTCAATAAAAGAGCCCACAACCCCTCACTCGGCGCGCCAGTCTTCCGATAGACTGCGTCGCCCGGGTACCCGTATTCCCAATAAAGCCTCTTGCTGTTTGCATCCGAATCGTGGTCTCGCTGTTCCTTGGGAGGGTCTCCTCTGAGTGATTGACTACCCACGACGGGGGTCTTTCATTTGGGGGCTCGTCCGGGATTTGGAGACCCCTGCCCAGGGACCACCGACCCACCACCGGGAGGTAAGCTGGCCAGCAACTTATCTGTGTCTGTCCGATTGTCTAGTGTCTATGTTTGATGTTATGCGCCTGCGTCTGTACTAGTTAGCTAACTAGCTCTGTATCTGGCGGACCCGTGGTGGAACTGACGAGTTCTGAACACCCGGCCGCAACCCTGGGAGACGTCCCAGGGACTTTGGGGGCCGTTTTTGTGGCCCGACCTGAGGAAGGGAGTCGATGTGGAATCCGACCCCGTCAGGATATGTGGTTCTGGTAGGAGACGAGAACCTAAAACAGTTCCCGCCTCCGTCTGAATTTTTGCTTTCGGTTTGGAACCGAAGCCGCGCGTCTTGTCTGCTGCAGCGCTGCAGCATCGTTCTGTGTTGTCTCTGTCTGACTGTGTTTCTGTATTTGTCTGAAAATTAGGGCCAGACTGTTACCACTCCCTTAAGTTTGACCTTAGGTCACTGGAAAGATGTCGAGCGGATCGCTCACAACCAGTCGGTAGATGTCAAGAAGAGACGTTGGGTTACCTTCTGCTCTGCAGAATGGCCAACCTTTAACGTCGGATGGCCGCGAGACGGCACCTTTAACCGAGACCTCATCACCCAGGTTAAGATCAAGGTCTTTTCACCTGGCCCGCATGGACACCCAGACCAGGTCCCCTACATCGTGACCTGGGAAGCCTTGGCTTTTGACCCCCCTCCCTGGGTCAAGCCCTTTGTACACCCTAAGCCTCCGCCTCCTCTTCCTCCATCCGCCCCGTCTCTCCCCCTTGAACCTCCTCGTTCGACCCCGCCTCGATCCTCCCTTTATCCAGCCCTCACTCCTTCTCTAGGCGCCGGAATTGAAGATCATCATGTCTGGATCCAAGCTGGAGGAAAAACTGTTTCATACAGAAGGCGTGGAGGAAAAACTGTTTCATACAGAAGGCGTGGAGGAAAAACTGTTTCATACAGAAGGCGTGGAGGAAAAACTGTTTCATACAGAAGGCGTGGAGGAAAAACTGTTTCATACAGAAGGCGTGGAGGAAAAACTGTTTCATACAGAAGGCGTGGAGGAAAAACTGTTTCATACAGAAGGCGTGGAGGAAAAACTGTTTCATACAGAAGGCGTCGCGGAGACTCTAGAGGGTATATAATGGAAGCTCGAATTCCAGTCCCCGAGATCTCGCCACCATGGCCCAGTCCAAGCACGGCCTGACCAAGGAGATGACCATGAAGTACCGCATGGAGGGCTGCGTGGACGGCCACAAGTTCGTGATCACCGGCGAGGGCATCGGCTACCCCTTCAAGGGCAAGCAGGCCATCAACCTGTGCGTGGTGGAGGGCGGCCCCTTGCCCTTCGCCGAGGACATCTTGTCCGCCGCCTTCATGTACGGCAACCGCGTGTTCACCGAGTACCCCCAGGACATCGTCGACTACTTCAAGAACTCCTGCCCCGCCGGCTACACCTGGGACCGCTCCTTCCTGTTCGAGGACGGCGCCGTGTGCATCTGCAACGCCGACATCACCGTGAGCGTGGAGGAGAACTGCATGTACCACGAGTCCAAGTTCTACGGCGTGAACTTCCCCGCCGACGGCCCCGTGATGAAGAAGATGACCGACAACTGGGAGCCCTCCTGCGAGAAGATCATCCCCGTGCCCAAGCAGGGCATCTTGAAGGGCGACGTGAGCATGTACCTGCTGCTGAAGGACGGTGGCCGCTTGCGCTGCCAGTTCGACACCGTGTACAAGGCCAAGTCCGTGCCCCGCAAGATGCCCGACTGGCACTTCATCCAGCACAAGCTGACCCGCGAGGACCGCAGCGACGCCAAGAACCAGAAGTGGCACCTGACCGAGCACGCCATCGCCTCCGGCTCCGCCTTGCCCTGACTCGAGGTTAACGAATTCTACCGGGTAGGGGAGGCGCTTTTCCCAAGGCAGTCTGGAGCATGCGCTTTAGCAGCCCCGCTGGGCACTTGGCGCTACACAAGTGGCCTCTGGCCTCGCACACATTCCACATCCACCGGTAGGCGCCAACCGGCTCCGTTCTTTGGTGGCCCCTTCGCGCCACCTTCTACTCCTCCCCTAGTCAGGAAGTTCCCCCCCGCCCCGCAGCTCGCGTCGTGCAGGACGTGACAAATGGAAGTAGCACGTCTCACTAGTCTCGTGCAGATGGACAGCACCGCTGAGCAATGGAAGCGGGTAGGCCTTTGGGGCAGCGGCCAATAGCAGCTTTGCTCCTTCGCTTTCTGGGCTCAGAGGCTGGGAAGGGGTGGGTCCGGGGGCGGGCTCAGGGGCGGGCTCAGGGGCGGGGCGGGCGCCCGAAGGTCCTCCGGAGGCCCGGCATTCTGCACGCTTCAAAAGCGCACGTCTGCCGCGCTGTTCTCCTCTTCCTCATCTCCGGGCCTTTCGacctgcatcccgccaccATGGCCTTACCAGTGACCGCCTTGCTCCTGCCGCTGGCCTTGCTGCTCCACGCCGCCAGGCCGAGCCAGTTCCGGGTGTCGCCGCTGGATCGGACCTGGAACCTGGGCGAGACAGTGGAGCTGAAGTGCCAGGTGCTGCTGTCCAACCCGACGTCGGGCTGCTCGTGGCTCTTCCAGCCGCGCGGCGCCGCCGCCAGTCCCACCTTCCTCCTATACCTCTCCCAAAACAAGCCCAAGGCGGCCGAGGGGCTGGACACCCAGCGGTTCTCGGGCAAGAGGTTGGGGGACACCTTCGTCCTCACCCTGAGCGACTTCCGCCGAGAGAACGAGGGCTACTATTTCTGCTCGGCCCTGAGCAACTCCATCATGTACTTCAGCCACTTCGTGCCGGTCTTCCTGCCAGCGAAGCCCACCACGACGCCAGCGCCGCGACCACCAACACCGGCGCCCACCATCGCGTCGCAGCCCCTGTCCCTGCGCCCAGAGGCGTGCCGGCCAGCGGCGGGGGGCGCAGTGCACACGAGGGGGCTGGACTTCGCCTGTGATATCTACATCTGGGCGCCCTTGGCCGGGACTTGTGGGGTCCTTCTCCTGTCACTGGTTATCACCCTTTACTGCAACCACAGGAACCGAAGACGTGTTTGCAAATGTCCCCGGCCTGTGGTCAAATCGGGAGACAAGCCCAGCCTTTCGGCGAGATACGTCGGCTCCGGAGCCACGAACTTCTCTCTGTTAAAGCAAGCAGGAGACGTGGAAGAAAACCCCGGTCCCATGCGGCCGCGGCTGTGGCTCCTCCTGGCCGCGCAGCTGACAGTTCTCCATGGCAACTCAGTCCTCCAGCAGACCCCTGCATACATAAAGGTGCAAACCAACAAGATGGTGATGCTGTCCTGCGAAGCTAAAATCTCCCTCAGTAACATGCGCATCTACTGGCTGAGACAGCGCCAGGCACCGAGCAGTGACAGTCACCACGAGTTCCTGGCCCTCTGGGATTCCGCAAAAGGGACTATCCACGGTGAAGAGGTGGAACAGGAGAAGATAGCTGTGTTTCGGGATGCAAGCCGGTTCATTCTCAATCTCACAAGCGTGAAGCCGGAAGACAGTGGCATCTACTTCTGCATGATCGTCGGGAGCCCCGAGCTGACCTTCGGGAAGGGAACTCAGCTGAGTGTGGTTGATTTCCTTCCCACCACTGCCCAGCCCACCAAGAAGTCCACCCTCAAGAAGAGAGTGTGCCGGTTACCCAGGCCAGAGACCCAGAAGGGCCCACTTTGTAGCCCCATCACCCTTGGCCTGCTGGTGGCTGGCATCCTGGTTCTGCTGGTTTCCCTGGGAGTGGCCATCCACCTGTGCTGCCGGCGGAGGAGAGCCCGGCTTCGTTTCATGAAACAGCCTCAAGGGGAAGGTATATCAGGAACCTTTGTCCCCCAATGCCTGCATGGATACTACAGCAATACTACAACCTCACAGAAGCTGCTTAACCCATGGATCCTGAAAACATAGaagcttcaccagctgaagcctatagagtacgagccatagataaaataaaagattttatttagtctccagaaaaaggggggaatgaaagaccccacctgtaggtttggcaagctagcttaagtaacgccattttgcaaggcatggaaaaatacataactgagaatagagaagttcagatcaaggtcaggaacagatggaacagggtcgaccctagagaaccatcagatgtttccagggtgccccaaggacctgaaatgaccctgtgccttatttgaactaaccaatcagttcgcttctcgcttctgttcgcgcgcttctgctccccgagctcaataaaagagcccacaacccctcactcggggcgccagtcctccgattgactgagtcgcccgggtacccgtgtatccaataaaccctcttgcagttgcatccgacttgtggtctcgctgttccttgggagggtctcctctgagtgattgactacccgtcagcgggggtctttcatttgggggctcgtccgggatcgggagacccctgcccagggaccaccgacccaccaccgggaggtaagctggctgcctcgcgcgtttcggtgatgacggtgaaaacctctgacatgtgagcaaaaggccagcaaaaggccaggaaccgtaaaaaggccgcgttgctggcgtttttccataggctccgcccccctgacgagcatcacaaaaatcgacgctcaagtcagaggtggcgaaacccgacaggactataaagataccaggcgtttccccctggaagctccctcgtgcgctctcctgttccgaccctgccgcttaccggatacctgtccgcctttctcccttcgggaagcgtggcgctttctcatagctcacgctgtaggtatctcagttcggtgtaggtcgttcgctccaagctgggctgtgtgcacgaaccccccgttcagcccgaccgctgcgccttatccggtaactatcgtcttgagtccaacccggtaagacacgacttatcgccactggcagcagccactggtaacaggattagcagagcgaggtatgtaggcggtgctacagagttcttgaagtggtggcctaactacggctacactagaagaacagtatttggtatctgcgctctgctgaagccagttaccttcggaaaaagagttggtagctcttgatccggcaaacaaaccaccgctggtagcggtggtttttttgtttgcaagcagcagattacgcgcagaaaaaaaggatctcaagaagatcctttgatcttttctacggggtctgacgctcagtggaacgaaaactcacgttaagggattttggtcatgagattatcaaaaaggatcttcacctagatccttttaaattaaaaatgaagttttaaatcaatctaaagtatatatgagtaaacttggtctgacagttaccaatgcttaatcagtgaggcacctatctcagcgatctgtctatttcgttcatccatagttgcctgactccccgtcgtgtagataactacgatacgggagggcttaccatctggccccagtgctgcaatgataccgcgagacccacgctcaccggctccagatttatcagcaataaaccagccagccggaagggccgagcgcagaagtggtcctgcaactttatccgcctccatccagtctattaattgttgccgggaagctagagtaagtagttcgccagttaatagtttgcgcaacgttgttgccattgctacaggcatcgtggtgtcacgctcgtcgtttggtatggcttcattcagctccggttcccaacgatcaaggcgagttacatgatcccccatgttgtgcaaaaaagcggttagctccttcggtcctccgatcgttgtcagaagtaagttggccgcagtgttatcactcatggttatggcagcactgcataattctcttactgtcatgccatccgtaagatgcttttctgtgactggtgagtactcaaccaagtcattctgagaatagtgtatgcggcgaccgagttgctcttgcccggcgtcaatacgggataataccgcgccacatagcagaactttaaaagtgctcatcattggaaaacgttcttcggggcgaaaactctcaaggatcttaccgctgttgagatccagttcgatgtaacccactcgtgcacccaactgatcttcagcatcttttactttcaccagcgtttctgggtgagcaaaaacaggaaggcaaaatgccgcaaaaaagggaataagggcgacacggaaatgttgaatactcatactcttcctttttcaatattattgaagcatttatcagggttattgtctcatgagcggatacatatttgaatgtatttagaaaaataaacaaataggggttccgcgcacatttccccgaaaagtgccacctgacgtctaagaaaccattattatcatgacattaacctataaaaataggcgtatcacgaggccctttcgtctcgcgcgtttcggtgatgacggtgaaaacctctgacacatgcagctcccggagacggtcacagcttgtctgtaagcggatgccgggagcagacaagcccgtcagggcgcgtcagcgggtgttggcgggtgtcggggctggcttaactatgcggcatcagagcagattgtactgagagtgcaccatatgcggtgtgaaataccgcacagatgcgtaaggagaaaataccgcatcaggcgccattcgccattcaggctgcgcaactgttgggaagggcgatcggtgcgggcctcttcgctattacgccagctggcgaaagggggatgtgctgcaaggcgattaagttgggtaacgccagggttttcccagtcacgacgttgtaaaacgacggcgcaaggaatggtgcatgcaaggagatggcgcccaacagtcccccggccacggggcctgccaccatacccacgccgaaacaagcgctcatgagcccgaagtggcgagcccgatcttccccatcggtgatgtcggcgatataggcgccagcaaccgcacctgtggcgccggtgatgccggccacgatgcgtccggcgtagaggcgattagtccaatttgttaaagacag

**V77 (8xNFAT-TATA-Tom-hCD8)**

Sequence

GATATCCCAACATTACCGCCATGTTGACATTGATTATTGACTAGTTATTAATAGTAATCAATTACGGGGTCATTAGTTCATAGCCCATATATGGAGTTCCGCGTTACATAACTTACGGTAAATGGCCCGCCTGGCTGACCGCCCAACGACCCCCGCCCATTGACGTCAATAATGACGTATGTTCCCATAGTAACGCCAATAGGGACTTTCCATTGACGTCAATGGGTGGAGTATTTACGGTAAACTGCCCACTTGGCAGTACATCAAGTGTATCATATGCCAAGTACGCCCCCTATTGACGTCAATGACGGTAAATGGCCCGCCTGGCATTATGCCCAGTACATGACCTTATGGGACTTTCCTACTTGGCAGTACATCTACGTATTAGTCATCGCTATTACCATGGTGATGCGGTTTTGGCAGTACATCAATGGGCGTGGATAGCGGTTTGACTCACGGGGATTTCCAAGTCTCCACCCCATTGACGTCAATGGGAGTTTGTTTTGGCACCAAAATCAACGGGACTTTCCAAAATGTCGTAACAACTCCGCCCCATTGACGCAAATGGGCGGTAGGCGTGTACGGTGGGAGGTCTATATAAGCAGAGCTCAATAAAAGAGCCCACAACCCCTCACTCGGCGCGCCAGTCTTCCGATAGACTGCGTCGCCCGGGTACCCGTATTCCCAATAAAGCCTCTTGCTGTTTGCATCCGAATCGTGGTCTCGCTGTTCCTTGGGAGGGTCTCCTCTGAGTGATTGACTACCCACGACGGGGGTCTTTCATTTGGGGGCTCGTCCGGGATTTGGAGACCCCTGCCCAGGGACCACCGACCCACCACCGGGAGGTAAGCTGGCCAGCAACTTATCTGTGTCTGTCCGATTGTCTAGTGTCTATGTTTGATGTTATGCGCCTGCGTCTGTACTAGTTAGCTAACTAGCTCTGTATCTGGCGGACCCGTGGTGGAACTGACGAGTTCTGAACACCCGGCCGCAACCCTGGGAGACGTCCCAGGGACTTTGGGGGCCGTTTTTGTGGCCCGACCTGAGGAAGGGAGTCGATGTGGAATCCGACCCCGTCAGGATATGTGGTTCTGGTAGGAGACGAGAACCTAAAACAGTTCCCGCCTCCGTCTGAATTTTTGCTTTCGGTTTGGAACCGAAGCCGCGCGTCTTGTCTGCTGCAGCGCTGCAGCATCGTTCTGTGTTGTCTCTGTCTGACTGTGTTTCTGTATTTGTCTGAAAATTAGGGCCAGACTGTTACCACTCCCTTAAGTTTGACCTTAGGTCACTGGAAAGATGTCGAGCGGATCGCTCACAACCAGTCGGTAGATGTCAAGAAGAGACGTTGGGTTACCTTCTGCTCTGCAGAATGGCCAACCTTTAACGTCGGATGGCCGCGAGACGGCACCTTTAACCGAGACCTCATCACCCAGGTTAAGATCAAGGTCTTTTCACCTGGCCCGCATGGACACCCAGACCAGGTCCCCTACATCGTGACCTGGGAAGCCTTGGCTTTTGACCCCCCTCCCTGGGTCAAGCCCTTTGTACACCCTAAGCCTCCGCCTCCTCTTCCTCCATCCGCCCCGTCTCTCCCCCTTGAACCTCCTCGTTCGACCCCGCCTCGATCCTCCCTTTATCCAGCCCTCACTCCTTCTCTAGGCGCCGGAATTGAAGATCATCATGTCTGGATCCAAGCTGGAGGAAAAACTGTTTCATACAGAAGGCGTGGAGGAAAAACTGTTTCATACAGAAGGCGTGGAGGAAAAACTGTTTCATACAGAAGGCGTGGAGGAAAAACTGTTTCATACAGAAGGCGTGGAGGAAAAACTGTTTCATACAGAAGGCGTGGAGGAAAAACTGTTTCATACAGAAGGCGTGGAGGAAAAACTGTTTCATACAGAAGGCGTGGAGGAAAAACTGTTTCATACAGAAGGCGTCGCGGAGACTCTAGAGGGTATATAATGGAAGCTCGAATTCCAGTCCCCGAGATCTAGCCACCATGGTGAGCAAGGGCGAGGAGGTCATCAAAGAGTTCATGCGCTTCAAGGTGCGCATGGAGGGCTCCATGAACGGCCACGAGTTCGAGATCGAGGGCGAGGGCGAGGGCCGCCCCTACGAGGGCACCCAGACCGCCAAGCTGAAGGTGACCAAGGGCGGCCCCCTGCCCTTCGCCTGGGACATCCTGTCCCCCCAGTTCATGTACGGCTCCAAGGCGTACGTGAAGCACCCCGCCGACATCCCCGATTACAAGAAGCTGTCCTTCCCCGAGGGCTTCAAGTGGGAGCGCGTGATGAACTTCGAGGACGGCGGTCTGGTGACCGTGACCCAGGACTCCTCCCTGCAGGACGGCACGCTGATCTACAAGGTGAAGATGCGCGGCACCAACTTCCCCCCCGACGGCCCCGTAATGCAGAAGAAGACCATGGGCTGGGAGGCCTCCACCGAGCGCCTGTACCCCCGCGACGGCGTGCTGAAGGGCGAGATCCACCAGGCCCTGAAGCTGAAGGACGGCGGCCACTACCTGGTGGAGTTCAAGACCATCTACATGGCCAAGAAGCCCGTGCAACTGCCCGGCTACTACTACGTGGACACCAAGCTGGACATCACCTCCCACAACGAGGACTACACCATCGTGGAACAGTACGAGCGCTCCGAGGGCCGCCACCACCTGTTCCTGGGGCATGGCACCGGCAGCACCGGCAGCGGCAGCTCCGGCACCGCCTCCTCCGAGGACAACAACATGGCCGTCATCAAAGAGTTCATGCGCTTCAAGGTGCGCATGGAGGGCTCCATGAACGGCCACGAGTTCGAGATCGAGGGCGAGGGCGAGGGCCGCCCCTACGAGGGCACCCAGACCGCCAAGCTGAAGGTGACCAAGGGCGGCCCCCTGCCCTTCGCCTGGGACATCCTGTCCCCCCAGTTCATGTACGGCTCCAAGGCGTACGTGAAGCACCCCGCCGACATCCCCGATTACAAGAAGCTGTCCTTCCCCGAGGGCTTCAAGTGGGAGCGCGTGATGAACTTCGAGGACGGCGGTCTGGTGACCGTGACCCAGGACTCCTCCCTGCAGGACGGCACGCTGATCTACAAGGTGAAGATGCGCGGCACCAACTTCCCCCCCGACGGCCCCGTAATGCAGAAGAAGACCATGGGCTGGGAGGCCTCCACCGAGCGCCTGTACCCCCGCGACGGCGTGCTGAAGGGCGAGATCCACCAGGCCCTGAAGCTGAAGGACGGCGGCCACTACCTGGTGGAGTTCAAGACCATCTACATGGCCAAGAAGCCCGTGCAACTGCCCGGCTACTACTACGTGGACACCAAGCTGGACATCACCTCCCACAACGAGGACTACACCATCGTGGAACAGTACGAGCGCTCCGAGGGCCGCCACCACCTGTTCCTGTACGGCATGGACGAGCTGTACAAGTAGCTCGAGGTTAACGAATTCTACCGGGTAGGGGAGGCGCTTTTCCCAAGGCAGTCTGGAGCATGCGCTTTAGCAGCCCCGCTGGGCACTTGGCGCTACACAAGTGGCCTCTGGCCTCGCACACATTCCACATCCACCGGTAGGCGCCAACCGGCTCCGTTCTTTGGTGGCCCCTTCGCGCCACCTTCTACTCCTCCCCTAGTCAGGAAGTTCCCCCCCGCCCCGCAGCTCGCGTCGTGCAGGACGTGACAAATGGAAGTAGCACGTCTCACTAGTCTCGTGCAGATGGACAGCACCGCTGAGCAATGGAAGCGGGTAGGCCTTTGGGGCAGCGGCCAATAGCAGCTTTGCTCCTTCGCTTTCTGGGCTCAGAGGCTGGGAAGGGGTGGGTCCGGGGGCGGGCTCAGGGGCGGGCTCAGGGGCGGGGCGGGCGCCCGAAGGTCCTCCGGAGGCCCGGCATTCTGCACGCTTCAAAAGCGCACGTCTGCCGCGCTGTTCTCCTCTTCCTCATCTCCGGGCCTTTCGacctgcatcccgccaccATGGCCTTACCAGTGACCGCCTTGCTCCTGCCGCTGGCCTTGCTGCTCCACGCCGCCAGGCCGAGCCAGTTCCGGGTGTCGCCGCTGGATCGGACCTGGAACCTGGGCGAGACAGTGGAGCTGAAGTGCCAGGTGCTGCTGTCCAACCCGACGTCGGGCTGCTCGTGGCTCTTCCAGCCGCGCGGCGCCGCCGCCAGTCCCACCTTCCTCCTATACCTCTCCCAAAACAAGCCCAAGGCGGCCGAGGGGCTGGACACCCAGCGGTTCTCGGGCAAGAGGTTGGGGGACACCTTCGTCCTCACCCTGAGCGACTTCCGCCGAGAGAACGAGGGCTACTATTTCTGCTCGGCCCTGAGCAACTCCATCATGTACTTCAGCCACTTCGTGCCGGTCTTCCTGCCAGCGAAGCCCACCACGACGCCAGCGCCGCGACCACCAACACCGGCGCCCACCATCGCGTCGCAGCCCCTGTCCCTGCGCCCAGAGGCGTGCCGGCCAGCGGCGGGGGGCGCAGTGCACACGAGGGGGCTGGACTTCGCCTGTGATATCTACATCTGGGCGCCCTTGGCCGGGACTTGTGGGGTCCTTCTCCTGTCACTGGTTATCACCCTTTACTGCAACCACAGGAACCGAAGACGTGTTTGCAAATGTCCCCGGCCTGTGGTCAAATCGGGAGACAAGCCCAGCCTTTCGGCGAGATACGTCGGCTCCGGAGCCACGAACTTCTCTCTGTTAAAGCAAGCAGGAGACGTGGAAGAAAACCCCGGTCCCATGCGGCCGCGGCTGTGGCTCCTCCTGGCCGCGCAGCTGACAGTTCTCCATGGCAACTCAGTCCTCCAGCAGACCCCTGCATACATAAAGGTGCAAACCAACAAGATGGTGATGCTGTCCTGCGAAGCTAAAATCTCCCTCAGTAACATGCGCATCTACTGGCTGAGACAGCGCCAGGCACCGAGCAGTGACAGTCACCACGAGTTCCTGGCCCTCTGGGATTCCGCAAAAGGGACTATCCACGGTGAAGAGGTGGAACAGGAGAAGATAGCTGTGTTTCGGGATGCAAGCCGGTTCATTCTCAATCTCACAAGCGTGAAGCCGGAAGACAGTGGCATCTACTTCTGCATGATCGTCGGGAGCCCCGAGCTGACCTTCGGGAAGGGAACTCAGCTGAGTGTGGTTGATTTCCTTCCCACCACTGCCCAGCCCACCAAGAAGTCCACCCTCAAGAAGAGAGTGTGCCGGTTACCCAGGCCAGAGACCCAGAAGGGCCCACTTTGTAGCCCCATCACCCTTGGCCTGCTGGTGGCTGGCATCCTGGTTCTGCTGGTTTCCCTGGGAGTGGCCATCCACCTGTGCTGCCGGCGGAGGAGAGCCCGGCTTCGTTTCATGAAACAGCCTCAAGGGGAAGGTATATCAGGAACCTTTGTCCCCCAATGCCTGCATGGATACTACAGCAATACTACAACCTCACAGAAGCTGCTTAACCCATGGATCCTGAAAACATAGaagcttcaccagctgaagcctatagagtacgagccatagataaaataaaagattttatttagtctccagaaaaaggggggaatgaaagaccccacctgtaggtttggcaagctagcttaagtaacgccattttgcaaggcatggaaaaatacataactgagaatagagaagttcagatcaaggtcaggaacagatggaacagggtcgaccctagagaaccatcagatgtttccagggtgccccaaggacctgaaatgaccctgtgccttatttgaactaaccaatcagttcgcttctcgcttctgttcgcgcgcttctgctccccgagctcaataaaagagcccacaacccctcactcggggcgccagtcctccgattgactgagtcgcccgggtacccgtgtatccaataaaccctcttgcagttgcatccgacttgtggtctcgctgttccttgggagggtctcctctgagtgattgactacccgtcagcgggggtctttcatttgggggctcgtccgggatcgggagacccctgcccagggaccaccgacccaccaccgggaggtaagctggctgcctcgcgcgtttcggtgatgacggtgaaaacctctgacatgtgagcaaaaggccagcaaaaggccaggaaccgtaaaaaggccgcgttgctggcgtttttccataggctccgcccccctgacgagcatcacaaaaatcgacgctcaagtcagaggtggcgaaacccgacaggactataaagataccaggcgtttccccctggaagctccctcgtgcgctctcctgttccgaccctgccgcttaccggatacctgtccgcctttctcccttcgggaagcgtggcgctttctcatagctcacgctgtaggtatctcagttcggtgtaggtcgttcgctccaagctgggctgtgtgcacgaaccccccgttcagcccgaccgctgcgccttatccggtaactatcgtcttgagtccaacccggtaagacacgacttatcgccactggcagcagccactggtaacaggattagcagagcgaggtatgtaggcggtgctacagagttcttgaagtggtggcctaactacggctacactagaagaacagtatttggtatctgcgctctgctgaagccagttaccttcggaaaaagagttggtagctcttgatccggcaaacaaaccaccgctggtagcggtggtttttttgtttgcaagcagcagattacgcgcagaaaaaaaggatctcaagaagatcctttgatcttttctacggggtctgacgctcagtggaacgaaaactcacgttaagggattttggtcatgagattatcaaaaaggatcttcacctagatccttttaaattaaaaatgaagttttaaatcaatctaaagtatatatgagtaaacttggtctgacagttaccaatgcttaatcagtgaggcacctatctcagcgatctgtctatttcgttcatccatagttgcctgactccccgtcgtgtagataactacgatacgggagggcttaccatctggccccagtgctgcaatgataccgcgagacccacgctcaccggctccagatttatcagcaataaaccagccagccggaagggccgagcgcagaagtggtcctgcaactttatccgcctccatccagtctattaattgttgccgggaagctagagtaagtagttcgccagttaatagtttgcgcaacgttgttgccattgctacaggcatcgtggtgtcacgctcgtcgtttggtatggcttcattcagctccggttcccaacgatcaaggcgagttacatgatcccccatgttgtgcaaaaaagcggttagctccttcggtcctccgatcgttgtcagaagtaagttggccgcagtgttatcactcatggttatggcagcactgcataattctcttactgtcatgccatccgtaagatgcttttctgtgactggtgagtactcaaccaagtcattctgagaatagtgtatgcggcgaccgagttgctcttgcccggcgtcaatacgggataataccgcgccacatagcagaactttaaaagtgctcatcattggaaaacgttcttcggggcgaaaactctcaaggatcttaccgctgttgagatccagttcgatgtaacccactcgtgcacccaactgatcttcagcatcttttactttcaccagcgtttctgggtgagcaaaaacaggaaggcaaaatgccgcaaaaaagggaataagggcgacacggaaatgttgaatactcatactcttcctttttcaatattattgaagcatttatcagggttattgtctcatgagcggatacatatttgaatgtatttagaaaaataaacaaataggggttccgcgcacatttccccgaaaagtgccacctgacgtctaagaaaccattattatcatgacattaacctataaaaataggcgtatcacgaggccctttcgtctcgcgcgtttcggtgatgacggtgaaaacctctgacacatgcagctcccggagacggtcacagcttgtctgtaagcggatgccgggagcagacaagcccgtcagggcgcgtcagcgggtgttggcgggtgtcggggctggcttaactatgcggcatcagagcagattgtactgagagtgcaccatatgcggtgtgaaataccgcacagatgcgtaaggagaaaataccgcatcaggcgccattcgccattcaggctgcgcaactgttgggaagggcgatcggtgcgggcctcttcgctattacgccagctggcgaaagggggatgtgctgcaaggcgattaagttgggtaacgccagggttttcccagtcacgacgttgtaaaacgacggcgcaaggaatggtgcatgcaaggagatggcgcccaacagtcccccggccacggggcctgccaccatacccacgccgaaacaagcgctcatgagcccgaagtggcgagcccgatcttccccatcggtgatgtcggcgatataggcgccagcaaccgcacctgtggcgccggtgatgccggccacgatgcgtccggcgtagaggcgattagtccaatttgttaaagacag

**V79 (8xNFAT-TATA-CR-hCD8)**

Sequence

GATATCCCAACATTACCGCCATGTTGACATTGATTATTGACTAGTTATTAATAGTAATCAATTACGGGGTCATTAGTTCATAGCCCATATATGGAGTTCCGCGTTACATAACTTACGGTAAATGGCCCGCCTGGCTGACCGCCCAACGACCCCCGCCCATTGACGTCAATAATGACGTATGTTCCCATAGTAACGCCAATAGGGACTTTCCATTGACGTCAATGGGTGGAGTATTTACGGTAAACTGCCCACTTGGCAGTACATCAAGTGTATCATATGCCAAGTACGCCCCCTATTGACGTCAATGACGGTAAATGGCCCGCCTGGCATTATGCCCAGTACATGACCTTATGGGACTTTCCTACTTGGCAGTACATCTACGTATTAGTCATCGCTATTACCATGGTGATGCGGTTTTGGCAGTACATCAATGGGCGTGGATAGCGGTTTGACTCACGGGGATTTCCAAGTCTCCACCCCATTGACGTCAATGGGAGTTTGTTTTGGCACCAAAATCAACGGGACTTTCCAAAATGTCGTAACAACTCCGCCCCATTGACGCAAATGGGCGGTAGGCGTGTACGGTGGGAGGTCTATATAAGCAGAGCTCAATAAAAGAGCCCACAACCCCTCACTCGGCGCGCCAGTCTTCCGATAGACTGCGTCGCCCGGGTACCCGTATTCCCAATAAAGCCTCTTGCTGTTTGCATCCGAATCGTGGTCTCGCTGTTCCTTGGGAGGGTCTCCTCTGAGTGATTGACTACCCACGACGGGGGTCTTTCATTTGGGGGCTCGTCCGGGATTTGGAGACCCCTGCCCAGGGACCACCGACCCACCACCGGGAGGTAAGCTGGCCAGCAACTTATCTGTGTCTGTCCGATTGTCTAGTGTCTATGTTTGATGTTATGCGCCTGCGTCTGTACTAGTTAGCTAACTAGCTCTGTATCTGGCGGACCCGTGGTGGAACTGACGAGTTCTGAACACCCGGCCGCAACCCTGGGAGACGTCCCAGGGACTTTGGGGGCCGTTTTTGTGGCCCGACCTGAGGAAGGGAGTCGATGTGGAATCCGACCCCGTCAGGATATGTGGTTCTGGTAGGAGACGAGAACCTAAAACAGTTCCCGCCTCCGTCTGAATTTTTGCTTTCGGTTTGGAACCGAAGCCGCGCGTCTTGTCTGCTGCAGCGCTGCAGCATCGTTCTGTGTTGTCTCTGTCTGACTGTGTTTCTGTATTTGTCTGAAAATTAGGGCCAGACTGTTACCACTCCCTTAAGTTTGACCTTAGGTCACTGGAAAGATGTCGAGCGGATCGCTCACAACCAGTCGGTAGATGTCAAGAAGAGACGTTGGGTTACCTTCTGCTCTGCAGAATGGCCAACCTTTAACGTCGGATGGCCGCGAGACGGCACCTTTAACCGAGACCTCATCACCCAGGTTAAGATCAAGGTCTTTTCACCTGGCCCGCATGGACACCCAGACCAGGTCCCCTACATCGTGACCTGGGAAGCCTTGGCTTTTGACCCCCCTCCCTGGGTCAAGCCCTTTGTACACCCTAAGCCTCCGCCTCCTCTTCCTCCATCCGCCCCGTCTCTCCCCCTTGAACCTCCTCGTTCGACCCCGCCTCGATCCTCCCTTTATCCAGCCCTCACTCCTTCTCTAGGCGCCGGAATTGAAGATCATCATGTCTGGATCCAAGCTGGAGGAAAAACTGTTTCATACAGAAGGCGTGGAGGAAAAACTGTTTCATACAGAAGGCGTGGAGGAAAAACTGTTTCATACAGAAGGCGTGGAGGAAAAACTGTTTCATACAGAAGGCGTGGAGGAAAAACTGTTTCATACAGAAGGCGTGGAGGAAAAACTGTTTCATACAGAAGGCGTGGAGGAAAAACTGTTTCATACAGAAGGCGTGGAGGAAAAACTGTTTCATACAGAAGGCGTCGCGGAGACTCTAGAGGGTATATAATGGAAGCTCGAATTCCAGTCCCCGAGATCTCGCCACCATGGATAGCACTGAGAACGTCATCAAGCCCTTCATGCGCTTCAAGGTGCACATGGAGGGCTCCGTGAACGGCCACGAGTTCGAGATCGAGGGCGTGGGCGAGGGCAAGCCCTACGAGGGCACCCAGACCGCCAAGCTGCAAGTGACCAAGGGCGGCCCCCTGCCCTTCGCCTGGGACATCCTGTCCCCCCAGTTCTTCTACGGCTCCAAGGCGTACATCAAGCACCCCGCCGACATCCCCGACTACCTCAAGCAGTCCTTCCCCGAGGGCTTCAAGTGGGAGCGCGTGATGAACTTCGAGGACGGCGGCGTGGTGACCGTGACCCAGGACTCCTCCCTGCAGGACGGCACCCTCATCTACCACGTGAAGTTCATCGGCGTGAACTTCCCCTCCGACGGCCCCGTAATGCAGAAGAAGACTCTGGGCTGGGAGCCCTCCACTGAGCGCAACTACCCCCGCGACGGCGTGCTGAAGGGCGAGAACCACATGGCGCTGAAGCTGAAGGGCGGCGGCCACTACCTGTGTGAGTTCAAGTCCATCTACATGGCCAAGAAGCCCGTGAAGCTGCCCGGCTACCACTACGTGGACTACAAGCTCGACATCACCTCCCACAACGAGGACTACACCGTGGTGGAGCAGTACGAGCGCGCCGAGGCCCGCCACCACCTGTTCCAGTAGCTCGAGGTTAACGAATTCTACCGGGTAGGGGAGGCGCTTTTCCCAAGGCAGTCTGGAGCATGCGCTTTAGCAGCCCCGCTGGGCACTTGGCGCTACACAAGTGGCCTCTGGCCTCGCACACATTCCACATCCACCGGTAGGCGCCAACCGGCTCCGTTCTTTGGTGGCCCCTTCGCGCCACCTTCTACTCCTCCCCTAGTCAGGAAGTTCCCCCCCGCCCCGCAGCTCGCGTCGTGCAGGACGTGACAAATGGAAGTAGCACGTCTCACTAGTCTCGTGCAGATGGACAGCACCGCTGAGCAATGGAAGCGGGTAGGCCTTTGGGGCAGCGGCCAATAGCAGCTTTGCTCCTTCGCTTTCTGGGCTCAGAGGCTGGGAAGGGGTGGGTCCGGGGGCGGGCTCAGGGGCGGGCTCAGGGGCGGGGCGGGCGCCCGAAGGTCCTCCGGAGGCCCGGCATTCTGCACGCTTCAAAAGCGCACGTCTGCCGCGCTGTTCTCCTCTTCCTCATCTCCGGGCCTTTCGacctgcatcccgccaccATGGCCTTACCAGTGACCGCCTTGCTCCTGCCGCTGGCCTTGCTGCTCCACGCCGCCAGGCCGAGCCAGTTCCGGGTGTCGCCGCTGGATCGGACCTGGAACCTGGGCGAGACAGTGGAGCTGAAGTGCCAGGTGCTGCTGTCCAACCCGACGTCGGGCTGCTCGTGGCTCTTCCAGCCGCGCGGCGCCGCCGCCAGTCCCACCTTCCTCCTATACCTCTCCCAAAACAAGCCCAAGGCGGCCGAGGGGCTGGACACCCAGCGGTTCTCGGGCAAGAGGTTGGGGGACACCTTCGTCCTCACCCTGAGCGACTTCCGCCGAGAGAACGAGGGCTACTATTTCTGCTCGGCCCTGAGCAACTCCATCATGTACTTCAGCCACTTCGTGCCGGTCTTCCTGCCAGCGAAGCCCACCACGACGCCAGCGCCGCGACCACCAACACCGGCGCCCACCATCGCGTCGCAGCCCCTGTCCCTGCGCCCAGAGGCGTGCCGGCCAGCGGCGGGGGGCGCAGTGCACACGAGGGGGCTGGACTTCGCCTGTGATATCTACATCTGGGCGCCCTTGGCCGGGACTTGTGGGGTCCTTCTCCTGTCACTGGTTATCACCCTTTACTGCAACCACAGGAACCGAAGACGTGTTTGCAAATGTCCCCGGCCTGTGGTCAAATCGGGAGACAAGCCCAGCCTTTCGGCGAGATACGTCGGCTCCGGAGCCACGAACTTCTCTCTGTTAAAGCAAGCAGGAGACGTGGAAGAAAACCCCGGTCCCATGCGGCCGCGGCTGTGGCTCCTCCTGGCCGCGCAGCTGACAGTTCTCCATGGCAACTCAGTCCTCCAGCAGACCCCTGCATACATAAAGGTGCAAACCAACAAGATGGTGATGCTGTCCTGCGAAGCTAAAATCTCCCTCAGTAACATGCGCATCTACTGGCTGAGACAGCGCCAGGCACCGAGCAGTGACAGTCACCACGAGTTCCTGGCCCTCTGGGATTCCGCAAAAGGGACTATCCACGGTGAAGAGGTGGAACAGGAGAAGATAGCTGTGTTTCGGGATGCAAGCCGGTTCATTCTCAATCTCACAAGCGTGAAGCCGGAAGACAGTGGCATCTACTTCTGCATGATCGTCGGGAGCCCCGAGCTGACCTTCGGGAAGGGAACTCAGCTGAGTGTGGTTGATTTCCTTCCCACCACTGCCCAGCCCACCAAGAAGTCCACCCTCAAGAAGAGAGTGTGCCGGTTACCCAGGCCAGAGACCCAGAAGGGCCCACTTTGTAGCCCCATCACCCTTGGCCTGCTGGTGGCTGGCATCCTGGTTCTGCTGGTTTCCCTGGGAGTGGCCATCCACCTGTGCTGCCGGCGGAGGAGAGCCCGGCTTCGTTTCATGAAACAGCCTCAAGGGGAAGGTATATCAGGAACCTTTGTCCCCCAATGCCTGCATGGATACTACAGCAATACTACAACCTCACAGAAGCTGCTTAACCCATGGATCCTGAAAACATAGaagcttcaccagctgaagcctatagagtacgagccatagataaaataaaagattttatttagtctccagaaaaaggggggaatgaaagaccccacctgtaggtttggcaagctagcttaagtaacgccattttgcaaggcatggaaaaatacataactgagaatagagaagttcagatcaaggtcaggaacagatggaacagggtcgaccctagagaaccatcagatgtttccagggtgccccaaggacctgaaatgaccctgtgccttatttgaactaaccaatcagttcgcttctcgcttctgttcgcgcgcttctgctccccgagctcaataaaagagcccacaacccctcactcggggcgccagtcctccgattgactgagtcgcccgggtacccgtgtatccaataaaccctcttgcagttgcatccgacttgtggtctcgctgttccttgggagggtctcctctgagtgattgactacccgtcagcgggggtctttcatttgggggctcgtccgggatcgggagacccctgcccagggaccaccgacccaccaccgggaggtaagctggctgcctcgcgcgtttcggtgatgacggtgaaaacctctgacatgtgagcaaaaggccagcaaaaggccaggaaccgtaaaaaggccgcgttgctggcgtttttccataggctccgcccccctgacgagcatcacaaaaatcgacgctcaagtcagaggtggcgaaacccgacaggactataaagataccaggcgtttccccctggaagctccctcgtgcgctctcctgttccgaccctgccgcttaccggatacctgtccgcctttctcccttcgggaagcgtggcgctttctcatagctcacgctgtaggtatctcagttcggtgtaggtcgttcgctccaagctgggctgtgtgcacgaaccccccgttcagcccgaccgctgcgccttatccggtaactatcgtcttgagtccaacccggtaagacacgacttatcgccactggcagcagccactggtaacaggattagcagagcgaggtatgtaggcggtgctacagagttcttgaagtggtggcctaactacggctacactagaagaacagtatttggtatctgcgctctgctgaagccagttaccttcggaaaaagagttggtagctcttgatccggcaaacaaaccaccgctggtagcggtggtttttttgtttgcaagcagcagattacgcgcagaaaaaaaggatctcaagaagatcctttgatcttttctacggggtctgacgctcagtggaacgaaaactcacgttaagggattttggtcatgagattatcaaaaaggatcttcacctagatccttttaaattaaaaatgaagttttaaatcaatctaaagtatatatgagtaaacttggtctgacagttaccaatgcttaatcagtgaggcacctatctcagcgatctgtctatttcgttcatccatagttgcctgactccccgtcgtgtagataactacgatacgggagggcttaccatctggccccagtgctgcaatgataccgcgagacccacgctcaccggctccagatttatcagcaataaaccagccagccggaagggccgagcgcagaagtggtcctgcaactttatccgcctccatccagtctattaattgttgccgggaagctagagtaagtagttcgccagttaatagtttgcgcaacgttgttgccattgctacaggcatcgtggtgtcacgctcgtcgtttggtatggcttcattcagctccggttcccaacgatcaaggcgagttacatgatcccccatgttgtgcaaaaaagcggttagctccttcggtcctccgatcgttgtcagaagtaagttggccgcagtgttatcactcatggttatggcagcactgcataattctcttactgtcatgccatccgtaagatgcttttctgtgactggtgagtactcaaccaagtcattctgagaatagtgtatgcggcgaccgagttgctcttgcccggcgtcaatacgggataataccgcgccacatagcagaactttaaaagtgctcatcattggaaaacgttcttcggggcgaaaactctcaaggatcttaccgctgttgagatccagttcgatgtaacccactcgtgcacccaactgatcttcagcatcttttactttcaccagcgtttctgggtgagcaaaaacaggaaggcaaaatgccgcaaaaaagggaataagggcgacacggaaatgttgaatactcatactcttcctttttcaatattattgaagcatttatcagggttattgtctcatgagcggatacatatttgaatgtatttagaaaaataaacaaataggggttccgcgcacatttccccgaaaagtgccacctgacgtctaagaaaccattattatcatgacattaacctataaaaataggcgtatcacgaggccctttcgtctcgcgcgtttcggtgatgacggtgaaaacctctgacacatgcagctcccggagacggtcacagcttgtctgtaagcggatgccgggagcagacaagcccgtcagggcgcgtcagcgggtgttggcgggtgtcggggctggcttaactatgcggcatcagagcagattgtactgagagtgcaccatatgcggtgtgaaataccgcacagatgcgtaaggagaaaataccgcatcaggcgccattcgccattcaggctgcgcaactgttgggaagggcgatcggtgcgggcctcttcgctattacgccagctggcgaaagggggatgtgctgcaaggcgattaagttgggtaacgccagggttttcccagtcacgacgttgtaaaacgacggcgcaaggaatggtgcatgcaaggagatggcgcccaacagtcccccggccacggggcctgccaccatacccacgccgaaacaagcgctcatgagcccgaagtggcgagcccgatcttccccatcggtgatgtcggcgatataggcgccagcaaccgcacctgtggcgccggtgatgccggccacgatgcgtccggcgtagaggcgattagtccaatttgttaaagacag

**V81 (8xNFAT-TATA-BFP-hCD8)**

Sequence

GATATCCCAACATTACCGCCATGTTGACATTGATTATTGACTAGTTATTAATAGTAATCAATTACGGGGTCATTAGTTCATAGCCCATATATGGAGTTCCGCGTTACATAACTTACGGTAAATGGCCCGCCTGGCTGACCGCCCAACGACCCCCGCCCATTGACGTCAATAATGACGTATGTTCCCATAGTAACGCCAATAGGGACTTTCCATTGACGTCAATGGGTGGAGTATTTACGGTAAACTGCCCACTTGGCAGTACATCAAGTGTATCATATGCCAAGTACGCCCCCTATTGACGTCAATGACGGTAAATGGCCCGCCTGGCATTATGCCCAGTACATGACCTTATGGGACTTTCCTACTTGGCAGTACATCTACGTATTAGTCATCGCTATTACCATGGTGATGCGGTTTTGGCAGTACATCAATGGGCGTGGATAGCGGTTTGACTCACGGGGATTTCCAAGTCTCCACCCCATTGACGTCAATGGGAGTTTGTTTTGGCACCAAAATCAACGGGACTTTCCAAAATGTCGTAACAACTCCGCCCCATTGACGCAAATGGGCGGTAGGCGTGTACGGTGGGAGGTCTATATAAGCAGAGCTCAATAAAAGAGCCCACAACCCCTCACTCGGCGCGCCAGTCTTCCGATAGACTGCGTCGCCCGGGTACCCGTATTCCCAATAAAGCCTCTTGCTGTTTGCATCCGAATCGTGGTCTCGCTGTTCCTTGGGAGGGTCTCCTCTGAGTGATTGACTACCCACGACGGGGGTCTTTCATTTGGGGGCTCGTCCGGGATTTGGAGACCCCTGCCCAGGGACCACCGACCCACCACCGGGAGGTAAGCTGGCCAGCAACTTATCTGTGTCTGTCCGATTGTCTAGTGTCTATGTTTGATGTTATGCGCCTGCGTCTGTACTAGTTAGCTAACTAGCTCTGTATCTGGCGGACCCGTGGTGGAACTGACGAGTTCTGAACACCCGGCCGCAACCCTGGGAGACGTCCCAGGGACTTTGGGGGCCGTTTTTGTGGCCCGACCTGAGGAAGGGAGTCGATGTGGAATCCGACCCCGTCAGGATATGTGGTTCTGGTAGGAGACGAGAACCTAAAACAGTTCCCGCCTCCGTCTGAATTTTTGCTTTCGGTTTGGAACCGAAGCCGCGCGTCTTGTCTGCTGCAGCGCTGCAGCATCGTTCTGTGTTGTCTCTGTCTGACTGTGTTTCTGTATTTGTCTGAAAATTAGGGCCAGACTGTTACCACTCCCTTAAGTTTGACCTTAGGTCACTGGAAAGATGTCGAGCGGATCGCTCACAACCAGTCGGTAGATGTCAAGAAGAGACGTTGGGTTACCTTCTGCTCTGCAGAATGGCCAACCTTTAACGTCGGATGGCCGCGAGACGGCACCTTTAACCGAGACCTCATCACCCAGGTTAAGATCAAGGTCTTTTCACCTGGCCCGCATGGACACCCAGACCAGGTCCCCTACATCGTGACCTGGGAAGCCTTGGCTTTTGACCCCCCTCCCTGGGTCAAGCCCTTTGTACACCCTAAGCCTCCGCCTCCTCTTCCTCCATCCGCCCCGTCTCTCCCCCTTGAACCTCCTCGTTCGACCCCGCCTCGATCCTCCCTTTATCCAGCCCTCACTCCTTCTCTAGGCGCCGGAATTGAAGATCATCATGTCTGGATCCAAGCTGGAGGAAAAACTGTTTCATACAGAAGGCGTGGAGGAAAAACTGTTTCATACAGAAGGCGTGGAGGAAAAACTGTTTCATACAGAAGGCGTGGAGGAAAAACTGTTTCATACAGAAGGCGTGGAGGAAAAACTGTTTCATACAGAAGGCGTGGAGGAAAAACTGTTTCATACAGAAGGCGTGGAGGAAAAACTGTTTCATACAGAAGGCGTGGAGGAAAAACTGTTTCATACAGAAGGCGTCGCGGAGACTCTAGAGGGTATATAATGGAAGCTCGAATTCCAGTCCCCGAGATCTAGCCACCATGGTGTCTAAGGGCGAAGAGCTGATTAAGGAGAACATGCACATGAAGCTGTACATGGAGGGCACCGTGGACAACCATCACTTCAAGTGCACATCCGAGGGCGAAGGCAAGCCCTACGAGGGCACCCAGACCATGAGAATCAAGGTGGTCGAGGGCGGCCCTCTCCCCTTCGCCTTCGACATCCTGGCTACTAGCTTCCTCTACGGCAGCAAGACCTTCATCAACCACACCCAGGGCATCCCCGACTTCTTCAAGCAGTCCTTCCCTGAGGGCTTCACATGGGAGAGAGTCACCACATACGAAGACGGGGGCGTGCTGACCGCTACCCAGGACACCAGCCTCCAGGACGGCTGCCTCATCTACAACGTCAAGATCAGAGGGGTGAACTTCACATCCAACGGCCCTGTGATGCAGAAGAAAACACTCGGCTGGGAGGCCTTCACCGAGACGCTGTACCCCGCTGACGGCGGCCTGGAAGGCAGAAACGACATGGCCCTGAAGCTCGTGGGCGGGAGCCATCTGATCGCAAACGCCAAGACCACATATAGATCCAAGAAACCCGCTAAGAACCTCAAGATGCCTGGCGTCTACTATGTGGACTACAGACTGGAAAGAATCAAGGAGGCCAACAACGAGACCTACGTCGAGCAGCACGAGGTGGCAGTGGCCAGATACTGCGACCTCCCTAGCAAACTGGGGCACAAGCTTAATTGACTCGAGGTTAACGAATTCTACCGGGTAGGGGAGGCGCTTTTCCCAAGGCAGTCTGGAGCATGCGCTTTAGCAGCCCCGCTGGGCACTTGGCGCTACACAAGTGGCCTCTGGCCTCGCACACATTCCACATCCACCGGTAGGCGCCAACCGGCTCCGTTCTTTGGTGGCCCCTTCGCGCCACCTTCTACTCCTCCCCTAGTCAGGAAGTTCCCCCCCGCCCCGCAGCTCGCGTCGTGCAGGACGTGACAAATGGAAGTAGCACGTCTCACTAGTCTCGTGCAGATGGACAGCACCGCTGAGCAATGGAAGCGGGTAGGCCTTTGGGGCAGCGGCCAATAGCAGCTTTGCTCCTTCGCTTTCTGGGCTCAGAGGCTGGGAAGGGGTGGGTCCGGGGGCGGGCTCAGGGGCGGGCTCAGGGGCGGGGCGGGCGCCCGAAGGTCCTCCGGAGGCCCGGCATTCTGCACGCTTCAAAAGCGCACGTCTGCCGCGCTGTTCTCCTCTTCCTCATCTCCGGGCCTTTCGacctgcatcccgccaccATGGCCTTACCAGTGACCGCCTTGCTCCTGCCGCTGGCCTTGCTGCTCCACGCCGCCAGGCCGAGCCAGTTCCGGGTGTCGCCGCTGGATCGGACCTGGAACCTGGGCGAGACAGTGGAGCTGAAGTGCCAGGTGCTGCTGTCCAACCCGACGTCGGGCTGCTCGTGGCTCTTCCAGCCGCGCGGCGCCGCCGCCAGTCCCACCTTCCTCCTATACCTCTCCCAAAACAAGCCCAAGGCGGCCGAGGGGCTGGACACCCAGCGGTTCTCGGGCAAGAGGTTGGGGGACACCTTCGTCCTCACCCTGAGCGACTTCCGCCGAGAGAACGAGGGCTACTATTTCTGCTCGGCCCTGAGCAACTCCATCATGTACTTCAGCCACTTCGTGCCGGTCTTCCTGCCAGCGAAGCCCACCACGACGCCAGCGCCGCGACCACCAACACCGGCGCCCACCATCGCGTCGCAGCCCCTGTCCCTGCGCCCAGAGGCGTGCCGGCCAGCGGCGGGGGGCGCAGTGCACACGAGGGGGCTGGACTTCGCCTGTGATATCTACATCTGGGCGCCCTTGGCCGGGACTTGTGGGGTCCTTCTCCTGTCACTGGTTATCACCCTTTACTGCAACCACAGGAACCGAAGACGTGTTTGCAAATGTCCCCGGCCTGTGGTCAAATCGGGAGACAAGCCCAGCCTTTCGGCGAGATACGTCGGCTCCGGAGCCACGAACTTCTCTCTGTTAAAGCAAGCAGGAGACGTGGAAGAAAACCCCGGTCCCATGCGGCCGCGGCTGTGGCTCCTCCTGGCCGCGCAGCTGACAGTTCTCCATGGCAACTCAGTCCTCCAGCAGACCCCTGCATACATAAAGGTGCAAACCAACAAGATGGTGATGCTGTCCTGCGAAGCTAAAATCTCCCTCAGTAACATGCGCATCTACTGGCTGAGACAGCGCCAGGCACCGAGCAGTGACAGTCACCACGAGTTCCTGGCCCTCTGGGATTCCGCAAAAGGGACTATCCACGGTGAAGAGGTGGAACAGGAGAAGATAGCTGTGTTTCGGGATGCAAGCCGGTTCATTCTCAATCTCACAAGCGTGAAGCCGGAAGACAGTGGCATCTACTTCTGCATGATCGTCGGGAGCCCCGAGCTGACCTTCGGGAAGGGAACTCAGCTGAGTGTGGTTGATTTCCTTCCCACCACTGCCCAGCCCACCAAGAAGTCCACCCTCAAGAAGAGAGTGTGCCGGTTACCCAGGCCAGAGACCCAGAAGGGCCCACTTTGTAGCCCCATCACCCTTGGCCTGCTGGTGGCTGGCATCCTGGTTCTGCTGGTTTCCCTGGGAGTGGCCATCCACCTGTGCTGCCGGCGGAGGAGAGCCCGGCTTCGTTTCATGAAACAGCCTCAAGGGGAAGGTATATCAGGAACCTTTGTCCCCCAATGCCTGCATGGATACTACAGCAATACTACAACCTCACAGAAGCTGCTTAACCCATGGATCCTGAAAACATAGaagcttcaccagctgaagcctatagagtacgagccatagataaaataaaagattttatttagtctccagaaaaaggggggaatgaaagaccccacctgtaggtttggcaagctagcttaagtaacgccattttgcaaggcatggaaaaatacataactgagaatagagaagttcagatcaaggtcaggaacagatggaacagggtcgaccctagagaaccatcagatgtttccagggtgccccaaggacctgaaatgaccctgtgccttatttgaactaaccaatcagttcgcttctcgcttctgttcgcgcgcttctgctccccgagctcaataaaagagcccacaacccctcactcggggcgccagtcctccgattgactgagtcgcccgggtacccgtgtatccaataaaccctcttgcagttgcatccgacttgtggtctcgctgttccttgggagggtctcctctgagtgattgactacccgtcagcgggggtctttcatttgggggctcgtccgggatcgggagacccctgcccagggaccaccgacccaccaccgggaggtaagctggctgcctcgcgcgtttcggtgatgacggtgaaaacctctgacatgtgagcaaaaggccagcaaaaggccaggaaccgtaaaaaggccgcgttgctggcgtttttccataggctccgcccccctgacgagcatcacaaaaatcgacgctcaagtcagaggtggcgaaacccgacaggactataaagataccaggcgtttccccctggaagctccctcgtgcgctctcctgttccgaccctgccgcttaccggatacctgtccgcctttctcccttcgggaagcgtggcgctttctcatagctcacgctgtaggtatctcagttcggtgtaggtcgttcgctccaagctgggctgtgtgcacgaaccccccgttcagcccgaccgctgcgccttatccggtaactatcgtcttgagtccaacccggtaagacacgacttatcgccactggcagcagccactggtaacaggattagcagagcgaggtatgtaggcggtgctacagagttcttgaagtggtggcctaactacggctacactagaagaacagtatttggtatctgcgctctgctgaagccagttaccttcggaaaaagagttggtagctcttgatccggcaaacaaaccaccgctggtagcggtggtttttttgtttgcaagcagcagattacgcgcagaaaaaaaggatctcaagaagatcctttgatcttttctacggggtctgacgctcagtggaacgaaaactcacgttaagggattttggtcatgagattatcaaaaaggatcttcacctagatccttttaaattaaaaatgaagttttaaatcaatctaaagtatatatgagtaaacttggtctgacagttaccaatgcttaatcagtgaggcacctatctcagcgatctgtctatttcgttcatccatagttgcctgactccccgtcgtgtagataactacgatacgggagggcttaccatctggccccagtgctgcaatgataccgcgagacccacgctcaccggctccagatttatcagcaataaaccagccagccggaagggccgagcgcagaagtggtcctgcaactttatccgcctccatccagtctattaattgttgccgggaagctagagtaagtagttcgccagttaatagtttgcgcaacgttgttgccattgctacaggcatcgtggtgtcacgctcgtcgtttggtatggcttcattcagctccggttcccaacgatcaaggcgagttacatgatcccccatgttgtgcaaaaaagcggttagctccttcggtcctccgatcgttgtcagaagtaagttggccgcagtgttatcactcatggttatggcagcactgcataattctcttactgtcatgccatccgtaagatgcttttctgtgactggtgagtactcaaccaagtcattctgagaatagtgtatgcggcgaccgagttgctcttgcccggcgtcaatacgggataataccgcgccacatagcagaactttaaaagtgctcatcattggaaaacgttcttcggggcgaaaactctcaaggatcttaccgctgttgagatccagttcgatgtaacccactcgtgcacccaactgatcttcagcatcttttactttcaccagcgtttctgggtgagcaaaaacaggaaggcaaaatgccgcaaaaaagggaataagggcgacacggaaatgttgaatactcatactcttcctttttcaatattattgaagcatttatcagggttattgtctcatgagcggatacatatttgaatgtatttagaaaaataaacaaataggggttccgcgcacatttccccgaaaagtgccacctgacgtctaagaaaccattattatcatgacattaacctataaaaataggcgtatcacgaggccctttcgtctcgcgcgtttcggtgatgacggtgaaaacctctgacacatgcagctcccggagacggtcacagcttgtctgtaagcggatgccgggagcagacaagcccgtcagggcgcgtcagcgggtgttggcgggtgtcggggctggcttaactatgcggcatcagagcagattgtactgagagtgcaccatatgcggtgtgaaataccgcacagatgcgtaaggagaaaataccgcatcaggcgccattcgccattcaggctgcgcaactgttgggaagggcgatcggtgcgggcctcttcgctattacgccagctggcgaaagggggatgtgctgcaaggcgattaagttgggtaacgccagggttttcccagtcacgacgttgtaaaacgacggcgcaaggaatggtgcatgcaaggagatggcgcccaacagtcccccggccacggggcctgccaccatacccacgccgaaacaagcgctcatgagcccgaagtggcgagcccgatcttccccatcggtgatgtcggcgatataggcgccagcaaccgcacctgtggcgccggtgatgccggccacgatgcgtccggcgtagaggcgattagtccaatttgttaaagacag
