# Supplementary material for: Multiplex T Cell Stimulation Assay Utilizing a T Cell Activation Reporter-Based Detection System
Source: Front Immunol. 2020 Apr 9;11:633. doi: 10.3389/fimmu.2020.00633 (PMC7160884; doi:10.3389/fimmu.2020.00633)
Supplement: Supplementary file 1 [file Data_Sheet_1.zip › Supplementary Figures.DOCX]

**Supplementary Figure 1**


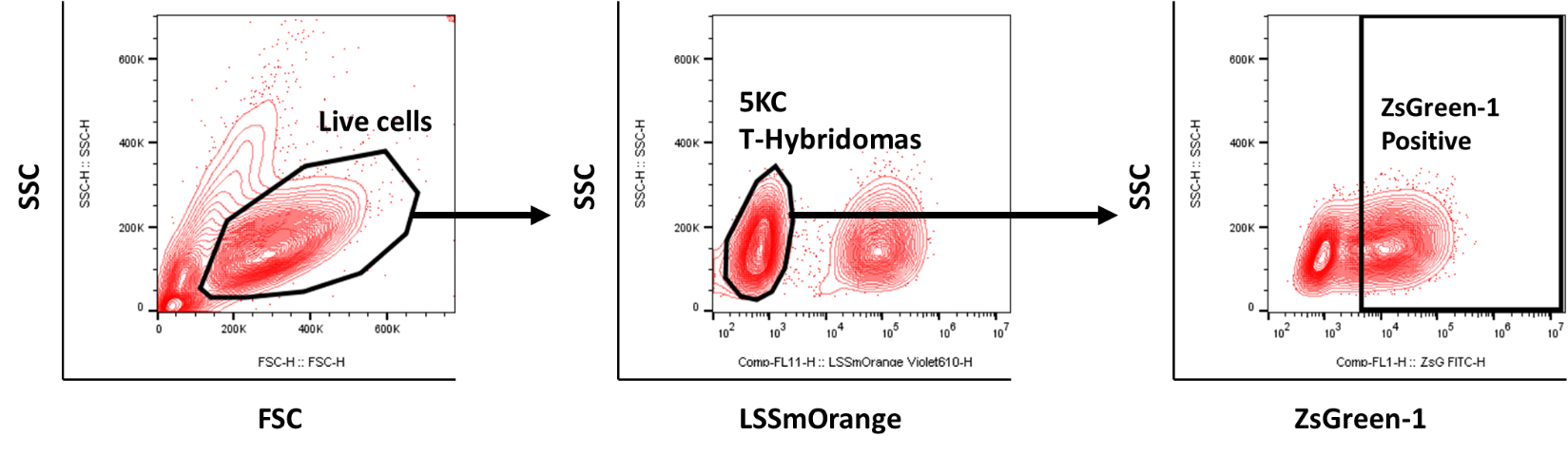


**Supplementary Figure 1:** Flow cytometry gating strategy used in experiments shown in figures 1, 2, 3, and 4. 5KC T-hybridoma cells were cultured with or without anti-CD3 antibody or peptides in the presence of K562 antigen presenting cells expressing cognate HLA molecules. K562 cells constitutively express LSSmOramge to distinguish from 5KC T-hybridoma cells. Cells from each culture condition were harvested and assessed on a flow-cytometer with 405 nm, 488 nm, and 633 nm lasers. Total 30,000 cells were acquired, live cells were selected based on forward and side scatter plots, and cells negative for LSSmOrange expression was selected as 5KC T-hybridomas. 5KC T-hybridoma cells without an NFAT reporter construct are included to determine the baseline fluorescent intensity that was used to establish gate placement for ZsGreen-1 positive cells.

**Supplementary Figure 2**


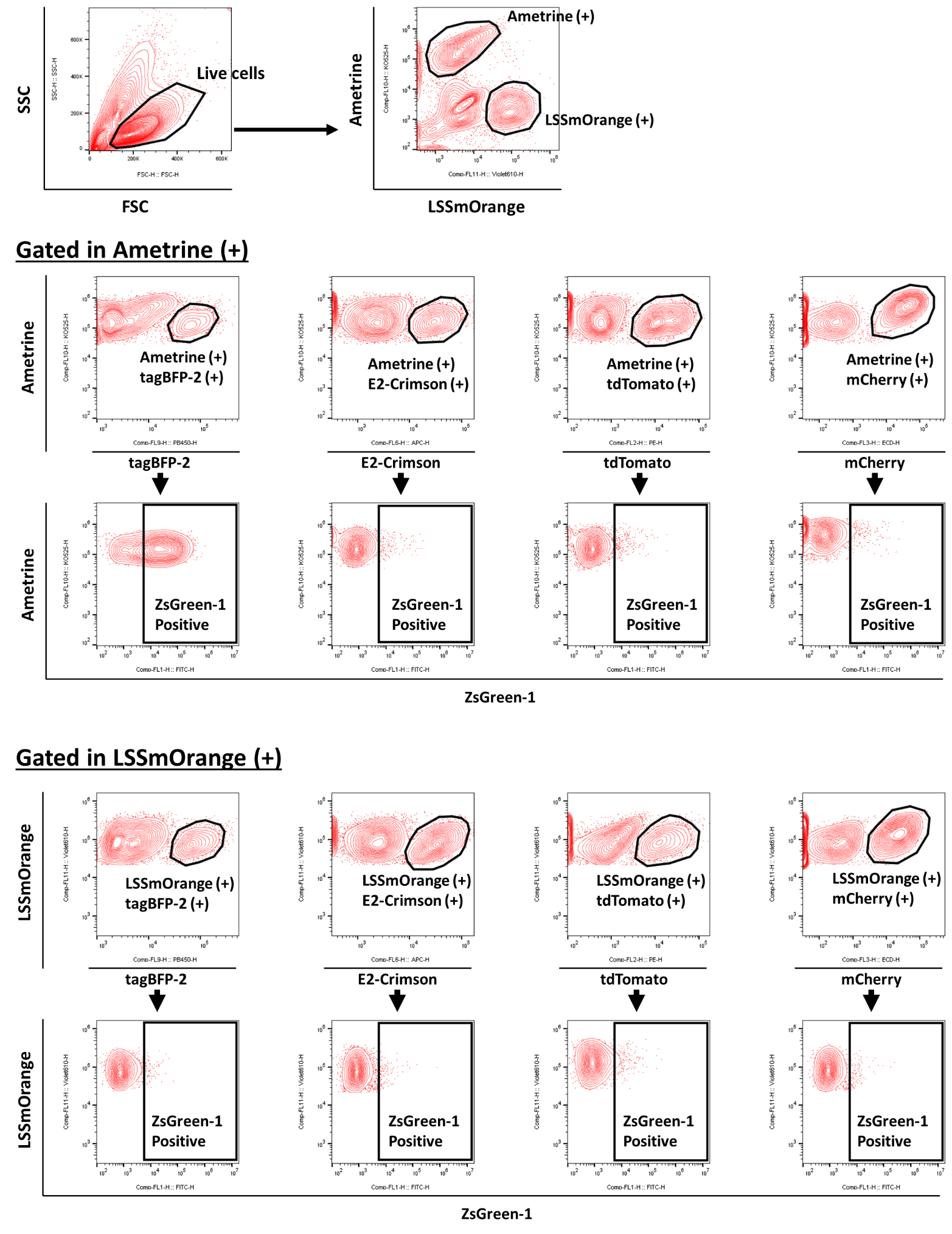


**Supplementary Figure 2:** Flow cytometry gating strategy used to distinguish between multiplexed 5KC T-hybridoma cells in experiments shown in Figures 5-7. T-hybridoma cells were cultured with or without peptides in the presence of K562 antigen presenting cells expressing cognate HLA molecules. Cells from each culture condition were harvested and assessed on a flow-cytometer with 405 nm, 488 nm, and 633 nm lasers. A total of 75,000 cells were acquired, live cells were selected based on forward and side scatter plots, and cells positive for LSSmOrange or Ametrine expression were selected as 5KC T-hybridomas. Ametrine+ and LSSmOrange+ cells were then gated for tagBFP-2, E2-Crimson, tdTomato, or mCherry expression to distinguish between cells expressing different TCRs, and finally ZsGreen-1 positivity was assessed. Each experiment also included wells without peptide to determine the baseline fluorescent intensity that was used to establish gate placement for ZsGreen-1 positive cells.
