# Supplementary material for: Multiplex T Cell Stimulation Assay Utilizing a T Cell Activation Reporter-Based Detection System
Source: Front Immunol. 2020 Apr 9;11:633. doi: 10.3389/fimmu.2020.00633 (PMC7160884; doi:10.3389/fimmu.2020.00633)
Supplement: Supplementary file 1 [file Data_Sheet_1.zip › Supplementary Tables.DOCX]

**Supplementary Table 1: NFAT-reporter retroviral vectors**

| **Vector ID** | **Vector Name** | **Insert** | **Backbone** | **Figures** |
| --- | --- | --- | --- | --- |
| V51-mu4 | pSIREN-muhCD4 | - | - | - |
| V51-8 | pSIREN-hCD8 | - | - | - |
| V64 | 6xNFAT-miniCMV-ZsG-muhCD4 | 6xNFAT_miniCMV_ZsGreen-1 | pSIREN-muhCD4 | Figure 1 |
| V65 | 6xNFAT-TATA-ZsG-muhCD4 | 6xNFAT_TATA-box_ZsGreen-1 | pSIREN-muhCD4 | Figure 1 |
| V152 | 4xNFAT-TATA-ZsG-muhCD4 | 4xNFAT_TATA-box_ZsGreen-1 | pSIREN-muhCD4 | Figure 2 |
| V74 | 8xNFAT-TATA-ZsG-muhCD4 | 8xNFAT_TATA-box_ZsGreen-1 | pSIREN-muhCD4 | Figure 2, 4 |
| V75 | 8xNFAT-TATA-ZsG-hCD8 | 8xNFAT_TATA-box_ZsGreen-1 | pSIREN-hCD8 | Figure 3, 4, 5, 6, 7 |
| V77 | 8xNFAT-TATA-Tom-hCD8 | 8xNFAT_TATA-box_tdTomato | pSIREN-hCD8 | Figure 3 |
| V79 | 8xNFAT-TATA-CR-hCD8 | 8xNFAT_TATA-box_E2-Crimson | pSIREN-hCD8 | Figure 3 |
| V81 | 8xNFAT-TATA-BFP-hCD8 | 8xNFAT_TATA-box_tagBFP2 | pSIREN-hCD8 | Figure 3 |

**Supplementary Table 2: Cell lines used in experiments**

**Supplementary Table 3: Amino acid sequences of preproinsulin truncated peptide pools**

**
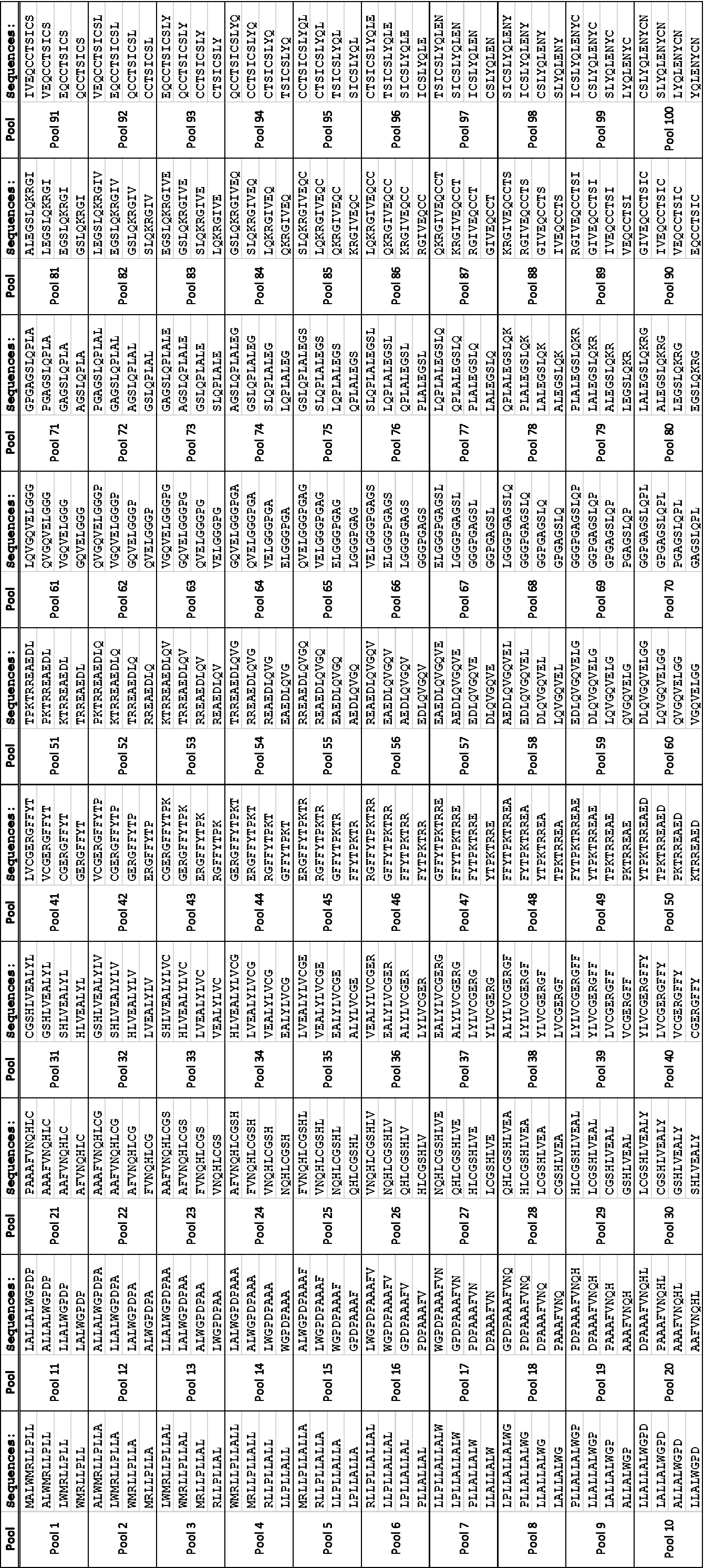
**

**Supplementary Table 4: Design of a decamer combinatorial peptide library**

**
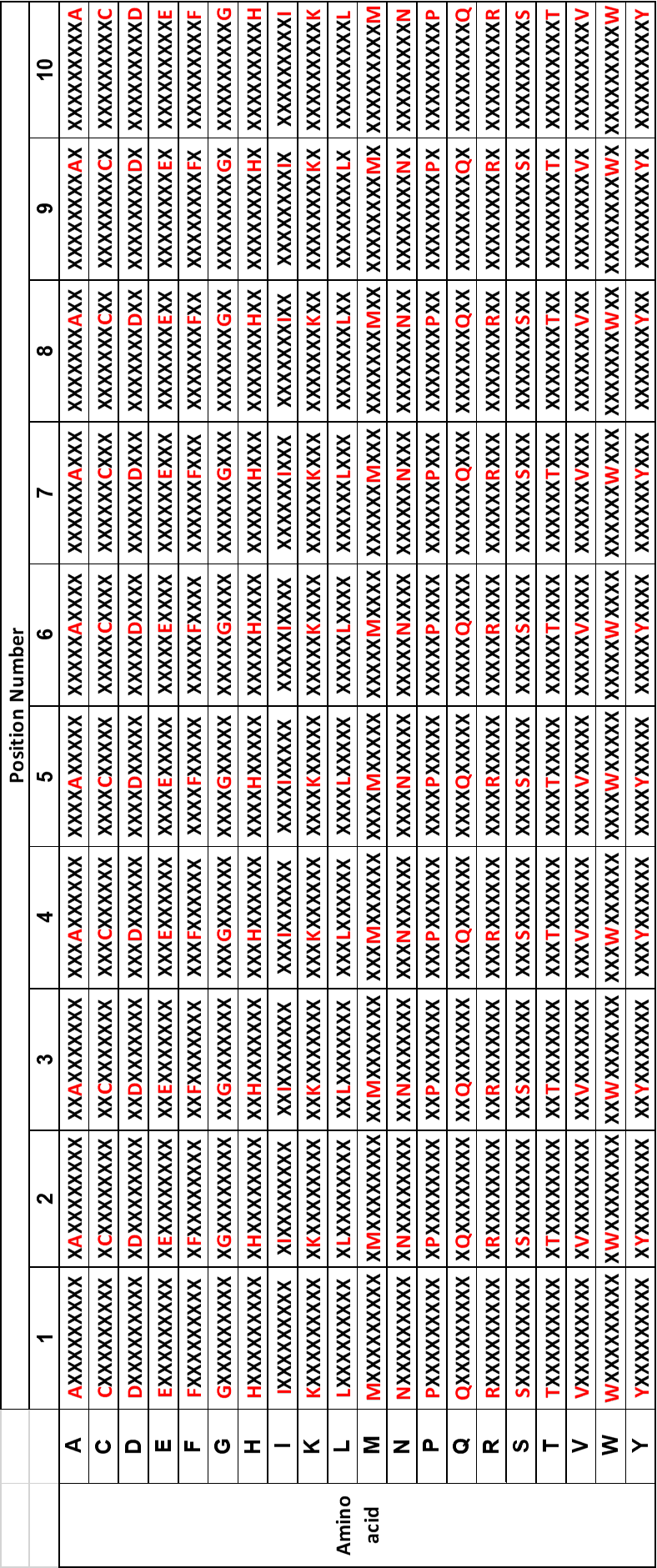
**
